# The post-fertilization archegonium gene network of seedless plants contributed to the origin of seeds

**DOI:** 10.1038/s41467-026-74577-w

**Published:** 2026-06-19

**Authors:** Andrew R. G. Plackett, Marco Catoni

**Affiliations:** School of Biosciences, University of Birmingham, Birmingham, UK

**Keywords:** Plant evolution, Seed development, Flowering, Fertilization

## Abstract

The evolution of seeds was a critical transition in plant evolution, but how ancestral seedless development was modified to form the first seed is only partly-answered through comparative morphology or the fossil record. We investigate seed origins by quantifying gene network conservation between seeds and seedless plant reproductive organs. Characterizing reproduction in the homosporous fern *Ceratopteris richardii* as a proxy for ancestral seedless reproduction, we create a gene expression atlas of fern diploid and haploid reproductive organ development and test for enrichment of these genes in *Arabidopsis thaliana*. Here we show ovule gene networks are enriched in genes regulating post-fertilization development of the fern haploid egg chamber (archegonium), a subset of which also show enrichment in sporophyll primordia, suggesting a mechanistic model to explain the origin of the ovule from a sporangium-bearing axis whereby gene networks in the ancestral post-fertilization archegonium became heterotopically expressed during development of the sporangium-bearing axis.

## Abstract (web summary)

The authors identify conserved gene networks across fern and angiosperm reproductive development, including between the sporophyte ovule and the fern gametophyte egg-chamber. Repurposing of this network could partly explain the ovule’s evolutionary origins.

## Introduction

Plants have not always made seeds, and the evolution of seed-based reproduction had a transformative impact on the Earth’s terrestrial biosphere. But how plants first evolved seeds has been a longstanding and intractable question. All living seed plants (gymnosperms and angiosperms) and seeds are inferred to have a single common ancestor^1,2^ arising from within the seedless vascular plants, with monilophytes (horsetails and ferns, hereafter “ferns”) the closest surviving outgroup^2,3^. The evolution of seed-based reproduction involved complex developmental modifications that resulted in a free-living stage of the plant lifecycle (the haploid gametophyte) becoming entirely dependent on another (the diploid sporophyte), in a reversal of the ancestral bryophyte lifecycle^4^, but how these occurred remains unclear.

Ovule patterning (the pre-fertilization stage of seed development) is ancient and conserved across extant and extinct seed plant lineages, recognisable from Palaeozoic seed ferns onwards: a diploid nucellus apically specialised for pollen reception, containing a multicellular haploid egg-producing structure, with a subtending chalaza from which one or more integuments arise^5^. After fertilization, this “body plan” undergoes further developmental changes to form the seed coat, provide nutrients to the embryo from storage tissues (the nucellus in some gymnosperms, the endosperm in angiosperms)^6–8^ and impose dormancy on the developing embryo^9^. Developmental changes involved in the ovule/seed’s origin have been deduced primarily through comparisons to ancestral vascular plant reproduction still found in extant ferns, where separate, free-living diploid and haploid “generations” both form reproductive organs^10,11^. The diploid sporophyte forms sporangia (generating single-celled haploid spores by meiosis) borne on fern lateral organs (fronds). Evidence increasingly supports the independent evolution of fern fronds and seed plant lateral organs (leaves)^12,13^, but this does not preclude their homology with ancestral shootlike structures (“axes”). Notably, fronds are more shoot-like than leaves, developing through a hierarchy of apical cells^14^, resulting in the characteristic fern “fiddlehead”. Fern spores germinate into thalloid gametophytes that produce gametangia by mitosis: antheridia (containing free-swimming sperm) and archegonia (egg-bearing chambers).

From such comparisons, it is inferred that early ovule evolution involved the invention of heterospory, i.e., “male” and “female” sporangia (anther and ovule nucellus) producing a genetically-predetermined male gametophyte (pollen) and female gametophyte (FG), from an ancestral homosporous sporangium^15^. Many extant ferns remain homosporous, including the genetic model *Ceratopteris richardii*^16^. Although gametophyte morphologies and reproductive strategies are diverse in extant ferns^17^, *Ceratopteris* default gametophyte development is hermaphroditic^18^. “Male” (micro)sporogenesis in seed plants seemingly resembles ancestral sporogenesis, whereas “female” (mega)sporogenesis has apparently undergone significant modification^19^, now typically producing a single viable megaspore (monospory)^20^. A second crucial early innovation was gametophyte encapsulation inside a sporangium (endospory)^21^ and developmental reduction, so extreme in angiosperms that archegonia are considered absent or potentially equivalent to the entire FG^22^. However, the ovule is more than the sum of its parts: ovule-specific developmental processes have evolved since its origin that do not have clear parallels, including apical specialisation for pollen capture and the endosperm as a nutrient storage tissue^5^. The origin of seeds thus entailed a complex suite of changes to ancestral seedless vascular plant development, but how development became reprogrammed to achieve this, and how novel developmental processes arose from existing gene networks, remains unclear.

Comparative fossil studies have proposed that the ovule evolved through modification of a sporangium-bearing shoot and progressive sterilization and reduction of branches to form the integument^21^. However, genetic analyses of extant ovule development, highlighting strong similarity between early ovule and meristematic development together with the modularity and lability of gene networks, challenge this view^23^, raising the possibility that integuments arose de novo and thus the basis for morphological comparisons may not be robust. Disparities between morphology-based and genetic-based analyses underly similar ambiguity over the evolutionary relationships between gymnosperms and angiosperms^24^. Even if correct, this model does not explain in detail how underlying gene networks were altered to change developmental patterning and other possibilities remain to be explored: like seeds, fern archegonia demonstrate fertilization-dependent developmental modifications to support embryo development, including post-fertilization growth of the envelope (calyptra) and the transfer of nutrients to the embryo via a placenta^25^. Fern sporophyte and gametophyte reproductive organs thus both possess seed-like characteristics, but archegonia were present within early Palaeozoic seeds and remain in most gymnosperm seeds^5^, seemingly precluding their incorporation into modifications leading to the ovule body-plan. The genetic changes underlying these evolutionary transitions remain unclear, but could be crucial to independently testing these hypotheses.

A complete molecular explanation for the origin of the ovule has not been proposed previously. A role for the MADS-box gene family, which specifies angiosperm ovule identity^26,27^ has long been suggested from indirect evidence^28,29^. Similarly, a seed maturation module centred on the gene *LEAFY COTYLEDON1* (*LEC1*) has been proposed to have originated in ferns^30^. Other studies of individual fern genes suggest at least partial conservation of reproductive gene networks between seedless and seed-bearing vascular plants^31,32^ but to-date the extent of this conservation has not been quantified. While fern genomic information is increasingly available^33–37^ as is transcriptome data at either whole-lifecycle scale^38–40^ or for single reproductive organs^34,41^, a detailed characterisation of gene networks across fern reproductive development to address this question is still lacking. We thus undertook mRNA-seq analysis to identify genes associated with reproductive processes in both the *Ceratopteris* sporophyte and gametophyte and compared these with *Arabidopsis thaliana* as the most detailed model available of seed development.

In this work, we find significant conservation between *Ceratopteris* and *Arabidopsis* reproductive gene networks, identifying a conserved role for the phytohormone gibberellin in shoot reproductive transitions and conserved genes underlying microsporogenesis and fern homosporous sporogenesis. We further identify significant gene conservation between the ovule and the fern post-fertilization archegonium that cannot be attributed to the FG within the ovule, suggesting that co-option of this gametophytic organ’s gene networks may have contributed to the ovule’s evolutionary origin.

## Results

### Identifying *Ceratopteris* sporophyte reproductive genes

**Fig. 1:**
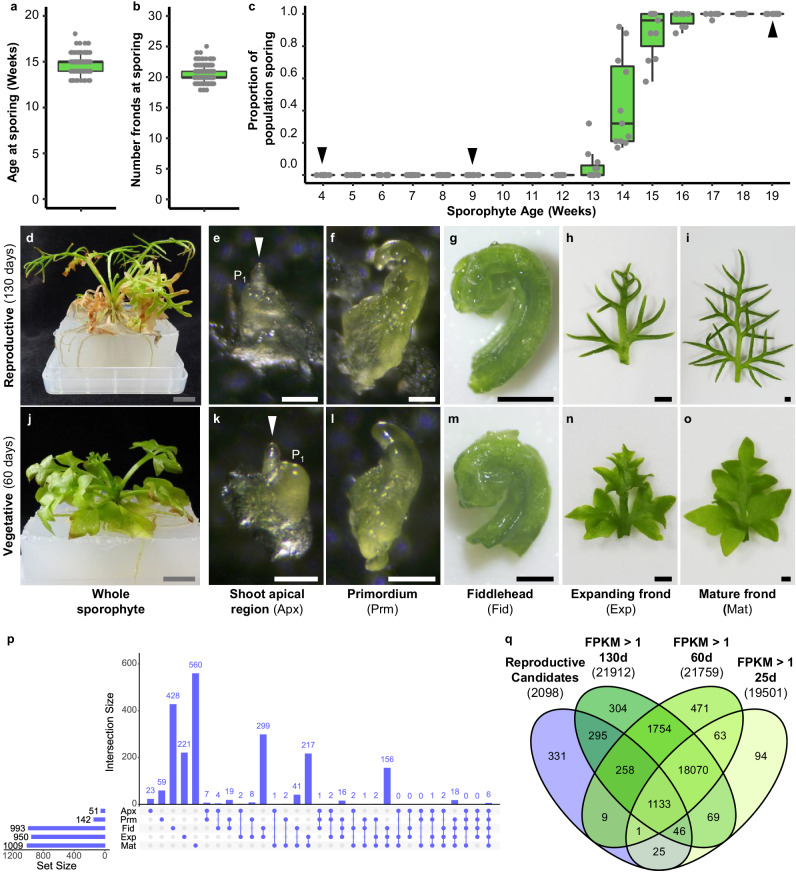
Reproduction-associated genes identified during *Ceratopteris* sporophyll development. **a–c** The timing and consistency of sporing of the *Ceratopteris* sporophyte. Plots show the mean sporing time by chronological age (**a**) and shoot position (**b**), and the proportion of sporophytes sporing over time per population (**c**). Black arrowheads denote informative shoot ages used in mRNA-seq experiments. Boxplots denote the data median and interquartile range (box) ±1.5× the interquartile range (whiskers). *n* = 271 (**a**, **b**) and 11 (**c**). **d–o** The reproductive-phase *Ceratopteris* shoot (**d**) was dissected for sequential samples across frond development: the shoot apical region (Apx) (**e**) comprising the shoot apex (white arrowhead) and early primordia (P_1_); the next-most recent primordium (Prm) (**f**) undergoing morphogenesis; the frond fiddlehead (Fid) (**g**) emerged from the shoot apical region; expanding fronds (Exp) (**h**) after the fiddlehead had terminated, from unfurling to approximately 75% of maturity; and mature non-senescent fronds (Mat) (**i**) where growth had ceased compared to prior fronds. The vegetative phase immediately preceding the sporing transition (**j**) was dissected for corresponding frond developmental stages (**k–o**). Candidate reproductive genes were identified through significantly increased expression (*P*_adj_ < 0.05, log2fold expression change > 1) in the sporophyll vs vegetative frond within each frond developmental stage. Scale bars = 10 mm (grey), 200 µm (white) and 1 mm (black). **p**, **q** Comparing reproductive candidate reproductive genes between frond developmental stages identified 2098 candidates (**p**). These were categorised based on transcript abundance (**q**): candidates at FPKM > 1 at 130 d but not in vegetative phases “sporophyll-specific”, at FPKM > 1 in both 130 d and at least one vegetative phase “sporophyll-enriched”, and at FPKM < 1 at 130 d as “sporophyll-FPKM < 1”. Candidate gene identities and corresponding expression categories are provided in Supplementary Data 2. Source data are provided as a **Source Data** file.

*Ceratopteris* sporophyte development was first characterised under tissue culture conditions (Supplementary Fig. 1). As under soil-grown conditions, successive fronds became increasingly dissected in a heteroblastic sequence until a transition to reproductive development (“sporing”) occurred. The *Ceratopteris* shoot was found to spore consistently and reproducibly measured by time (100% by 18 weeks) and development (20.6 ± 0.07 fronds) (Fig. 1a–c, Supplementary Fig. 2a, b, and Supplementary Data 1). To identify transcriptional changes specifically associated with reproductive development, using mRNA-seq we compared gene expression across frond development between sporophytes in the sporing phase (130 days old) (Fig. 1d–i) and the vegetative phase immediately prior to sporing (60 days old) (Fig. 1j–o and Supplementary Fig. 2c–e), with 25-day-old apex and primordia samples included as an early vegetative control (Supplementary Fig. 3a–e). Frond development was divided into sequential stages common to vegetative fronds and sporophylls: the shoot apical region (“Apx”) (Fig. 1e, k); frond primordium (“Prm”) (Fig. 1f, l and Supplementary Fig. 3f–q); fiddlehead (“Fid”) (Fig. 1g, m); expanding frond (“Exp”) (Fig. 1h, n) and mature frond (“Mat”) (Fig. 1i, o). Sporangia were already visible by the start of fiddlehead unfurling (Supplementary Fig. 4a–l), and sporangium development could not be classified into distinct stages based on frond size (Supplementary Fig. 4m, n) except that post-meiotic sporangia were only observed in full-expanded (Mat) samples (Supplementary Fig. 4e–h).

Sample clustering by gene expression using Principal Component Analysis (PCA) (Supplementary Fig. 5a) and correlation of mRNA-seq data (Supplementary Fig. 6) confirmed that clustering reflected frond developmental and reproductive changes. Frond stages separated progressively along PC1, with Apx and Prm samples closely overlapping except for 25 d Prm, which clustered more closely with Fid samples. The similarity of Apx and Prm samples is consistent with the common presence of primordia. Within each frond developmental stage, vegetative and reproductive phases separated across PC2. Differential expression (DE) analysis identified 2098 transcripts with increased abundance (*P*_adj_ < 0.05, log2foldchange > 1) between sporophylls and vegetative samples within at least one frond stage (Fig. 1p and Supplementary Data 2), 38.5% identified in more than one stage (35.3% from consecutive stages). Among these reproductive candidate genes we categorised 295 present (at a threshold of FPKM > 1) only in the sporophyll (“sporophyll-specific”), 1437 present in the sporophyll and at least one vegetative stage (“sporophyll-enriched”) and 366 candidates expressed at FPKM < 1 in all sporophyll samples (Fig. 1q). These expression categories represent potential direct regulators of reproductive processes, genes either with dual vegetative and reproductive function or frond genes whose expression is modified in sporophylls, and genes with either low or tissue-specific expression, respectively. We compared these candidates to genes with increasing abundance across frond development (*P*_adj_ < 0.05, log2foldchange > 1 between any two frond stages, comparing from earlier to later development), identifying differential regulation specifically in 130 d fronds (candidate sporophyll regulators) and co-regulation between 60 and 130 d frond stages (frond developmental regulators) (Supplementary Data 3). 62.8% of our reproductive candidates were represented in one or both of these classes (Supplementary Fig. 7), with sporophyll-specific and sporophyll-FPKM < 1 candidates showing significant enrichment (*P*_adj_ < 0.05) in sporophyll developmental regulators compared to the full dataset, whereas sporophyll-enriched candidates showed significant enrichment in frond developmental regulators. Our identification of candidate reproductive genes and the three expression categories were thus supported as robust.

### Gibberellin promotes the *Ceratopteris* sporing transition

**Fig. 2:**
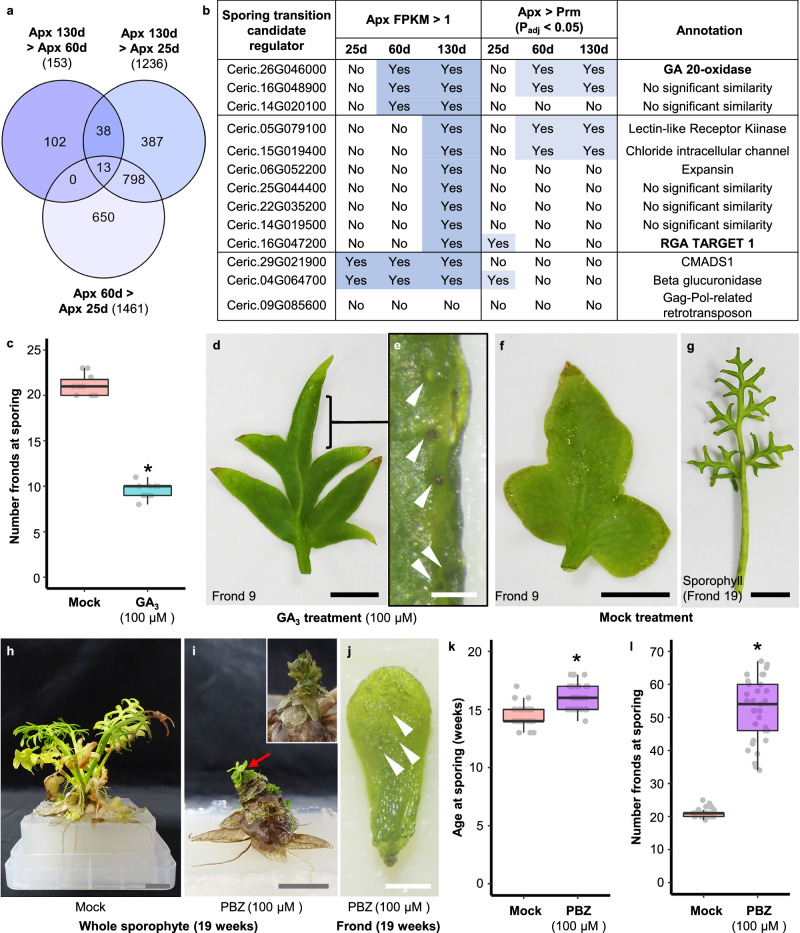
Gibberellin promotes the *Ceratopteris* sporing transition independently of frond heteroblasty. **a**, **b** Differential gene expression analysis identified 51 candidates with significantly greater expression in the 130 d Apx vs 60 and 25 d, with 13 showing a progressive increase immediately prior to the sporing transition (**a**). These were ranked according to the predicted expression characteristics of a shoot apex sporing regulator (**b**). GA-related annotations are highlighted in bold. **c** Comparison of the number of fronds at sporing under mock and exogenous GA treatment (100 µM GA_3_). Pairwise comparisons were performed on square root-transformed data using a two-tailed Mann–Whitney test. Asterisk denotes a significant difference (*P* = 0.0000928). Boxplots denote the data median and interquartile range (box) ±1.5× the interquartile range (whiskers). *n* = 11. **d–g** Under exogenous GA treatment sporangia typically first became visible on frond 9 (**d**, **e**) with margin rolling and sporangium production compared to mock-treated fronds at the same position (**f**), but otherwise are similar without increased dissection seen in mock-treated sporophylls at later frond positions (**g**). White arrowheads denote visible sporangia within the rolled frond margin. **h–j** Under exogenous PBZ treatment (100 µM), frond morphology became strongly simplified compared to mock-treated sporophytes (**h**, **i**). A phase-change nevertheless emerged as shoot development progressed (red arrow) (**i**, inset shows prolonged shoot development post phase-change), with fronds demonstrating increased hyponasty and pinna elongation (**j**). White arrowheads denote apparent immature sporangia on the abaxial surface. **k**, **l** Comparison of phase-change timing under PBZ treatment compared to sporing under mock treatment, measured chronologically (**k**) and in number of fronds produced per shoot (**l**). Pairwise comparisons were performed using a two-tailed Mann–Whitney test (chronological age, untransformed data; number of fronds, log10-transformed data). Asterisk denotes a significant difference (*P* = 0.00000004382 and 0.000000000000448, respectively). Boxplots denote the data median and interquartile range (box) ±1.5× the interquartile range (whiskers). *n* = 36 (mock), 33 (PBZ). Scale bars = 10 mm (black, grey), 500 µm (white). Statistical analysis are provided in Supplementary Data 1. Source data are provided as a **Source Data** file.

Genes involved in apex function were identified by comparing expression between Apx (shoot apex and primordia) and Prm (primordia only) samples, comparing between different shoot ages to identify genes associated with the shoot sporing transition. DE analysis identified 51 sporing transition candidates with significantly greater abundance (*P*_adj_ < 0.05) at 130 d compared to 60 and 25 d (Fig. 2a). Genes promoting a reproductive transition similar to angiosperm shoots were further predicted to increase expression immediately prior to the transition, and to be preferentially expressed in the shoot apex vs primordia. Thirteen sporing candidates showed progressive significant increases in abundance (*P*_adj_ < 0.05, log2foldchange > 1) between 25, 60, and 130 d Apx samples, including the previously-predicted MADS-box reproductive gene *CMADS1*^28^, but this was present at FPKM > 1 in all ages and not significantly more abundant in Apx vs Prm samples (*P*_adj_ > 0.05) (Fig. 2b), while two other MADS-box homologs identified as sporing candidates showed increased abundance at 130 d only (Supplementary Data 2).

Of two sporing candidates whose expression profile closely matched that predicted for sporing regulators, one was annotated as a *GA 20-OXIDASE*^42^ (Fig. 2b), supported by its phylogenetic position in a fern outgroup to seed plant *GA20ox* orthologs (Supplementary Fig. 8). Sporing candidates further included a putative homolog of *RGA TARGET 1* (*RGAT1*), a direct target of GA signalling in *Arabidopsis* pollen development^43^, whereas a putative *GA 3-OXIDASE* biosynthesis gene significantly decreases in abundance (*P*_adj_ < 0.05) at 130 d (Supplementary Data 2), a known homeostatic response to GA signalling^44^. These results suggested a role for GA in promoting *Ceratopteris* sporing. We tested this experimentally through exogenous application of GA_3_, which significantly accelerated the sporing transition (*P* < 0.05) (Fig. 2c). These did not phenocopy the frond dissection of natural sporophylls (Fig. 2d–g), suggesting that the sporing transition network is separable from frond heteroblasty. In a reciprocal test, we applied the canonical GA biosynthesis inhibitor paclobutrazol (PBZ)^45^ to sporophytes from 4 weeks onwards. In contrast to mock-treated controls, frond morphology reverted to simple spade-shaped fronds (Fig. 2h, i), but a morphological phase transition was still observed whereby new fronds were elongated, reminiscent of sporophyll pinnae (Fig. 2j) and bore apparent immature sporangia in 7/20 sporophytes examined. This phase transition was significantly delayed compared to sporing under mock treatment both chronologically and developmentally (Fig. 2k, l). These results are consistent with a conserved function for GA promoting the shoot reproductive transition between ferns and angiosperms, separably from a potential role in frond morphogenesis.

### Sporophyll reproductive genes include sporogenesis orthologs

**Fig. 3:**
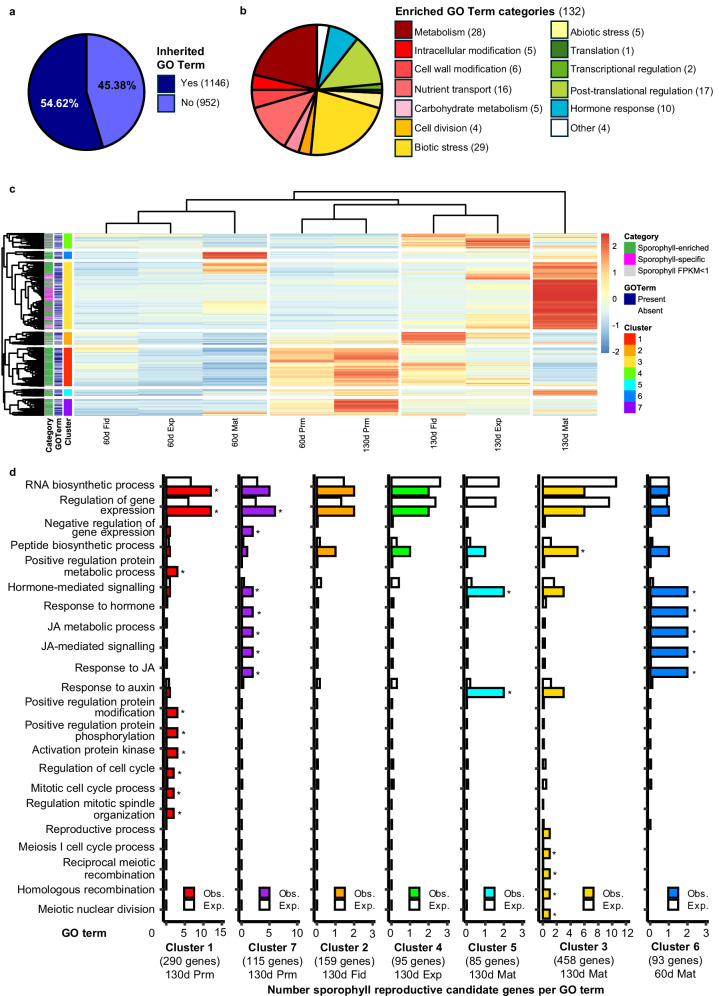
*Ceratopteris* sporophyll candidate reproductive genes include conserved meiosis and stamen developmental regulators. **a** The proportion of sporophyll reproductive candidate genes with GO terms annotated from orthologous genes. Numbers in brackets correspond to the frequency of genes within each category. **b** The proportion of GO term categories significantly enriched (*P* < 0.05) within annotated sporophyll reproductive candidates. Numbers in brackets correspond to the frequency of GO terms within each category. **c** Clustering of sporophyll reproductive candidate genes based on normalised expression identified seven discrete clusters of genes with maximum expression in specific stages of sporophyll development. The distribution of genes annotated with GO terms and of genes in sporophyll-specific, sporophyll-enriched and sporophyll-FPKM < 1 expression categories are shown within each cluster. **d** The frequency of sporophyll reproductive candidate genes with enriched reproductive GO terms within each cluster identified in (**c**) against the frequency expected based on the whole genome. Clusters are shown in order of their peak in abundance during sporophyll development. Obs. observed frequency, Exp. expected frequency. Enrichment testing employed Fisher's Exact Test (one-sided, greater than). Asterisks denote a significant increase (*P* < 0.05) in the frequency of genes with that GO term compared to the expected frequency. *P*-values for GO term enrichment testing are given in Supplementary Data 5. Source data are provided as a **Source Data** file.

54.6% of candidate genes possessed GO term annotations (Fig. 3a) and were significantly enriched in these (*P*_adj_ < 0.05) compared to the whole genome (Supplementary Data 5). GO term analysis identified significant enrichment (*P* < 0.05) predominantly of processes including metabolism, nutrition and stress response (Fig. 3b). Reproductive GO terms were not enriched in all candidates but were so in the sporophyll-specific expression category, relating to meiosis (Supplementary Fig. 9). Hierarchical clustering analysis of reproductive candidates by normalised expression identified seven expression clusters exhibiting maximum abundance in distinct stages across sporophyll development (Fig. 3c and Supplementary Data 4). Meiotic GO terms were significantly enriched (*P* < 0.05) in mature sporophylls (cluster 3) (Fig. 3d), consistent with our developmental characterization (Supplementary Fig. 4) and the only cluster significantly enriched in sporophyll-specific candidates (*P*_adj_ < 0.05; Supplementary Data 4). Meiotic GO terms resolved to a single gene annotated as an ortholog of *DISRUPTED MEIOTIC CDNA1* (*DMC1*) (Supplementary Data 5), a eukaryotic-conserved regulator of meiosis^46^. However, reproductive candidates included further annotated orthologs of regulators of meiotic recombination (*MSH4*, *ZIP4*)^47,48^, sporogenous cell fate (*EXS/EMS*, *TPD1*) and post-meiotic pollen development (*AMS1*, *MS1* and *MS2*)^49^. These suggest that our candidate dataset has successfully captured the sporogenesis pathway in *Ceratopteris*.

Beyond sporogenesis, GO term enrichment analysis identified potential mechanisms regulating sporophyll development. GO terms relating to hormone response were significantly enriched (*P* < 0.05) in sporophyll candidates (Fig. 3a), with auxin-related terms significantly enriched in mature sporophylls (cluster 5) and jasmonate (JA)-related terms enriched in sporophyll primordia (cluster 7) amongst others (cluster 6) (Fig. 3d). Sporophyll primordia were specifically enriched in GO terms relating to protein phosphorylation (cluster 1) and gene regulation (clusters 1 and 7), including two MADS-box homologs (Ceric.05G029400, Ceric.34G033600; Supplementary Data 5). Comparing GO term enrichment between sporophyll differentially-regulated genes and frond co-regulated genes found that hormone GO terms were enriched specifically during sporophyll development whereas phosphorylation was enriched in both (Supplementary Fig. 10). Remarkably, clusters with maximum expression during the fiddlehead (cluster 2) and expanding frond stages (cluster 4) were not enriched in recognisable regulatory pathways, enriched GO terms instead relating to metabolism (Supplementary Data 5). This was not due to poor annotation because these clusters demonstrated a similar proportion of annotated genes as the whole genome (*P*_adj_ > 0.05), as did clusters 5–7 (Supplementary Data 5). Collectively, these findings suggest that sporophyll-specific development is regulated at least in part through activating reproduction-specific regulatory mechanisms to modify frond regulatory pathways, primarily during early organ development and at the onset of sporogenesis.

### Identifying *Ceratopteris* gametophyte reproductive genes

**Fig. 4:**
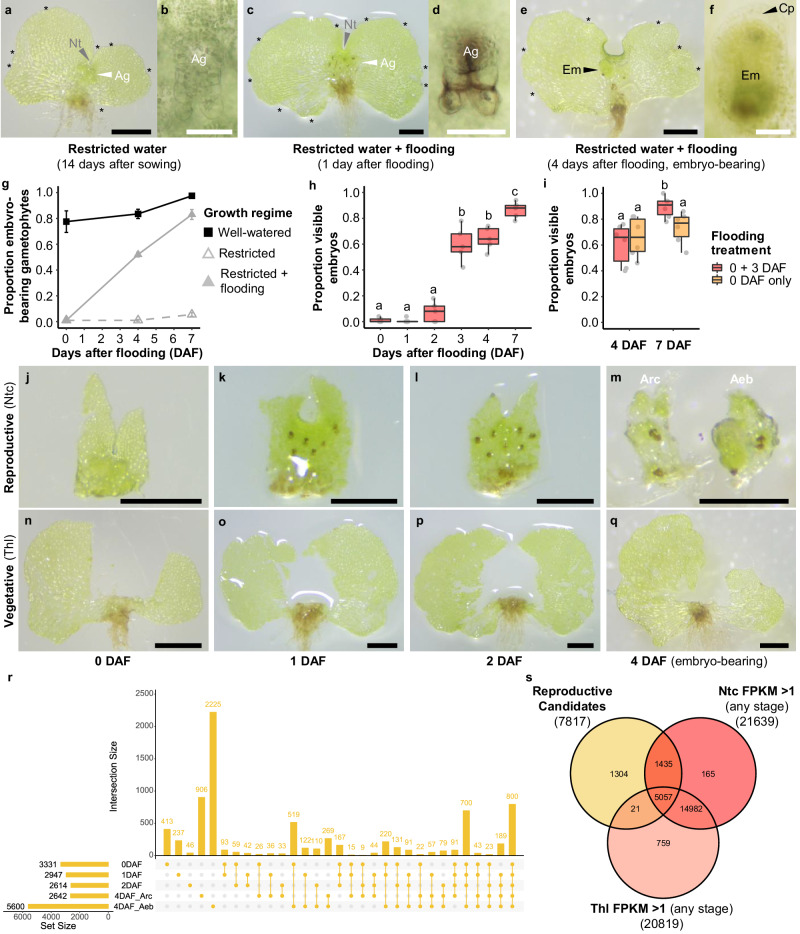
Identification of reproduction-associated genes in the *Ceratopteris* gametophyte. **a–f** *Ceratopteris* gametophyte and archegonium morphology before flooding (**a**, **b**), 1 day after flooding (DAF) (**c**, **d**) and 4 DAF after successful fertilization, as shown by a visible embryo (**e**, **f**). Ag archegonium, asterisk antheridia, Cp calyptra, Em embryo, Nt notch meristem. **g** The proportion of embryo-bearing gametophytes before and after flooding when grown to sexual maturity (14 days) under well-watered or restricted water conditions. Lineplot shows the mean ± SEM. *n* = 4. **h**, **i** The proportion of flooded gametophytes with visible embryos per DAF under repeated flooding (0 + 3 DAF) (**h**) or when flooded once (0 DAF only) (**i**). Boxplots show the median and interquartile range (box) ±1.5× interquartile range (whiskers). *n* = 5. Different letters denote a significant difference (*P*_adj_ < 0.05) between groups. Pairwise comparisons were performed using two-tailed Mann–Whitney tests on logit-transformed data (**h, i**) and corrected for multiple comparisons. *P-*values are given in Supplementary Data 6. **j–q** Gametophytes were dissected into the notch region (Ntc) containing the notch meristem and developing archegonia (**j**–**m**) and surrounding thallus tissue (Thl) (**n**–**q**) before fertilization (0 DAF; **j**, **n**); recently after flooding (1 DAF and 2 DAF; **k**, **l**, **o**, **p**) and once an embryo was visible (4 DAF; **m**, **q**). 4 DAF Ntc samples were subdivided into the archegonium bearing an embryo (Aeb) and embryo-less archegonia (Arc). Candidate reproductive genes were identified through significantly increased expression (*P*_adj_ < 0.05, log2fold expression change > 1) in the notch vs thallus within each stage. **r**, **s** Comparing reproductive candidate reproductive genes between gametophyte stages identified 7187 candidates (**r**). These were categorised based on transcript abundance (**s**): candidates expressed at FPKM > 1 in at least one Ntc sample but not in any Thl sample were categorised as “notch-specific”, those expressed at FPKM > 1 in both Ntc and Thl as “notch-enriched”, and those with FPKM < 1 in all Ntc samples as “notch-FPKM < 1”. Candidate gene identities and corresponding expression categories are provided in Supplementary Data 7.Scale bars = 500 µm (black), 50 µm (white). Source data are provided as a **Source Data** file.

The *Ceratopteris* hermaphrodite gametophyte (from hereon “gametophyte”) develops multiple archegonia from a “notch” meristem while antheridia develop at the thallus margin (Fig. 4a). Archegonia are fertilized by free-swimming sperm^25^ released in the presence of water. Green when unfertilized (Fig. 4b), all archegonia turn brown within one day of being flooded (Fig. 4c, d) but typically only a single embryo develops (Fig. 4e, f). Fertilization and embryo development were experimentally manipulated by restricting water availability: by 14 days after sowing (0 DAF) 77.5 ± 12.5% of well-watered controls bore embryos at various developmental stages (Supplementary Fig. 11) compared to 1.0 ± 1.0% under restricted water (Fig. 4g). Flooding of restricted-water populations successfully triggered fertilization, with 83.0 ± 5.74% bearing visible embryos by 7 DAF compared to 5.5 ± 2.2% without flooding. Under this system, most embryos became visible 3–4 DAF (Fig. 4h) and showed closer developmental synchronization (Supplementary Fig. 11), which was further improved by restricting water after initial flooding (Fig. 4i). The notch meristem also exhibited fertilization-dependent shut down of archegonium production by 2 DAF (Supplementary Fig. 12 and Supplementary Data 6).

To identify genes relating to archegonium formation, we dissected out the notch region (“Ntc”) from the gametophyte (Fig. 4j–m) and used the surrounding thallus (“Thl”) as a vegetative control (Fig. 4n–q). To further capture genes involved in fertilization and post-fertilization development, these samples were compared between unflooded gametophytes at 14 DAS (Fig. 4j, n) and at 1 (Fig. 4k, o), 2 (Fig. 4l, p) and 4 DAF (Fig. 4m, q). The 4 DAF notch was subdivided into embryo-bearing archegonia (“Aeb”) and embryo-less archegonia (“Arc”) (Fig. 4m). To discriminate embryo from archegonium gene networks, we isolated 4 DAF embryos as an additional control (“Dem”) (Supplementary Fig. 3r–w). PCA analysis (Supplementary Fig. 5b) and sample correlation (Supplementary Fig. 13) found that biological replicates clustered more closely within each sample than between different developmental stages except for 1 DAF and 2 DAF (which overlapped within the Ntc and Thl tissue types). PC1 separated Ntc from Thl and DAF separated progressively along PC2 (except for Dem). A total of 7817 reproductive candidate genes were identified with increased abundance in Ntc compared to Thl within at least one developmental stage (Fig. 4r and Supplementary Data 7). Similar to the sporophyll, candidate genes were categorised as “notch-specific” (1435), “notch-enriched” (5057) and “notch FPKM < 1” (1325) (Fig. 4s), representing potential direct reproductive regulators, downstream developmental gene targets and low or tissue-specific expression, respectively. Comparing these to genes demonstrating differential upregulation specific to the notch during development (Supplementary Fig. 14), 35.95% of notch reproductive candidates were represented within this dataset, with the notch-specific and notch-FPKM1 expression categories significantly enriched (*P*_adj_ < 0.05) compared to all candidates, whereas the notch-enriched category was significantly enriched in genes showing co-regulation between notch and thallus development (Supplementary Data 3). The successful detection of robust candidate notch reproductive genes was thus supported.

### Notch reproductive genes include seed development orthologs

**Fig. 5:**
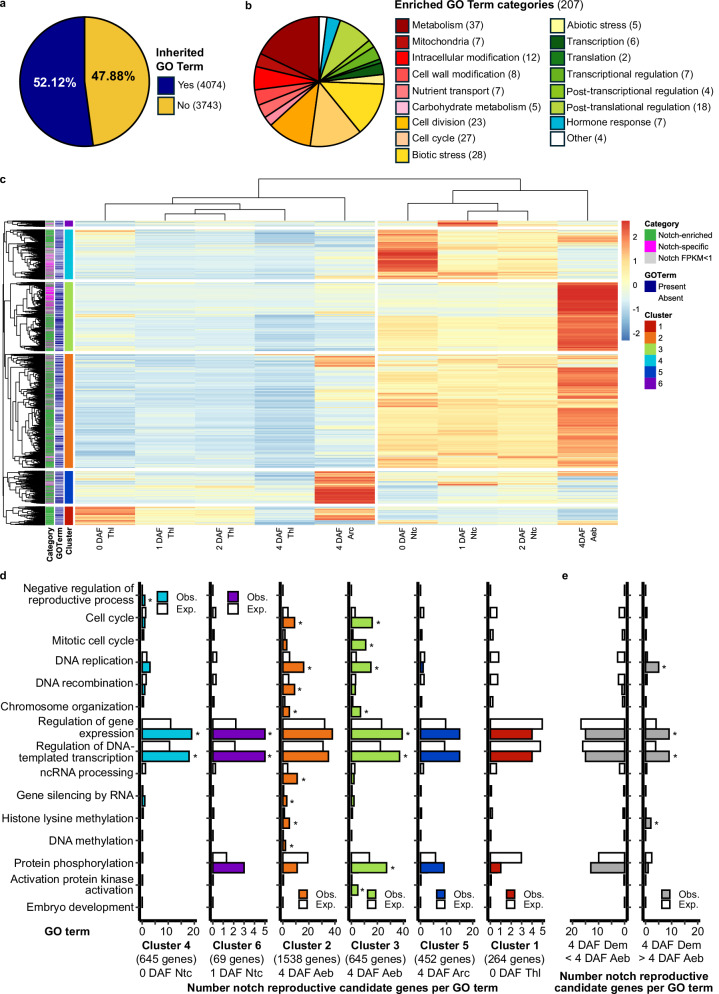
The *Ceratopteris* embryo-bearing archegonium is enriched in seed-related genes. **a**, **b** The proportion of *Ceratopteris* notch candidate genes with GO terms inherited from orthologous genes (**a**) and of different categories of significantly-enriched GO terms (*P* < 0.05) within all notch reproductive candidates (**b**). Numbers in brackets within the key correspond to the numbers of genes (**a**) or GO terms (**b**) within each category. **c** Clustering of notch reproductive candidate genes based on normalised expression identified six discrete clusters of genes with maximum expression in specific tissues during gametophyte reproductive development. **d**, **e** The frequency of observed notch reproductive candidate genes with individual reproductive GO terms against the frequency expected based on the whole genome, analysed within each cluster identified in (**c**), ordered by notch developmental stage (**d**), and within genes differentially-expressed (*P*_adj_ < 0.05, log2foldchange > 1) between Aeb and Dem samples at 4DAF (**e**). Obs. observed frequency, Exp. expected frequency. Enrichment testing employed Fisher's Exact Test (one-sided, greater than). Asterisks denote a significant increase (*P* < 0.05) in the frequency of genes with that GO term compared to the expected frequency. *P*-values for GO term enrichment testing are given in Supplementary Data 5. Source data are provided as a **Source Data** file.

52.1% of notch candidates possessed GO term annotation (Fig. 5a), significantly enriched (*P*_adj_ < 0.05) compared to the whole genome (Supplementary Data 5). 24.2% of significantly-enriched GO terms (*P* < 0.05) related to the cell cycle or cell division (Fig. 5b), in contrast to sporophyll candidates (3.0%). 17.9% of notch candidate enriched GO terms related to gene regulation, including post-transcriptional and post-translational categories. Our candidates thus reflect the notch’s status as an actively-dividing tissue and suggest multiple regulatory mechanisms are involved in its development.

Hierarchical clustering of notch candidates by normalised expression successfully identified six clusters with greatest transcript abundance in distinct notch developmental stages (Fig. 5c). Cluster 4, with expression greatest in the pre-fertilization notch, was significantly enriched in notch-specific candidates (*P*_adj_ < 0.05; Supplementary Data 4) and GO terms relating to the regulation of gene expression and negative regulation of reproduction (Fig. 5d). This revealed putative orthologs of the *TFL1-like* PEBP subfamily (Ceric.19G00340) and the *SIN3* family of transcription factors (Ceric.02G103600, Ceric.02G103700; Supplementary Data 5) which regulate the timing of *Arabidopsis* flowering^50,51^. Cluster 6, where expression peaks immediately after fertilization and during notch meristem shutdown, was also significantly enriched for GO terms regulating gene expression, revealing a putative ortholog of the HB21/40/53 transcription factors (Ceric.07G084400), which in *Arabidopsis* trigger inflorescence meristem arrest^52^. These similarities suggest that fern notch meristem gene networks could be conserved with seed plant reproductive meristem gene networks.

GO terms for the regulation of cell division and gene expression via post-transcriptional and post-translational regulatory mechanisms (noncoding RNA, protein phosphorylation, histone and DNA methylation) were enriched specifically in clusters with expression greatest in the Aeb (clusters 2 and 3) (Fig. 5d), with cluster 3 also significantly enriched in notch-specific candidates and genes differentially-upregulated specifically during notch development (Supplementary Data 4). The genes associated with these GO terms include multiple putative orthologs of *Arabidopsis* genes with functions across ovule and seed development (Supplementary Table 1), including integument development, megaspore mother cell (MMC) specification, FG development, embryogenesis and endosperm development. Surprisingly, many were annotated as orthologs of genes with functions in *Arabidopsis* meiosis (*MSH2*, *MSH7*, *SAE2*, *RAD51C*, *TOP43A*, *RFC1*)^53–58^. A proportion of these enriched GO terms could be ascribed to transcripts with significantly greater abundance (*P*_adj_ < 0.05, log2fold > 1) in Dem vs Aeb samples (Fig. 5e). Two further candidate genes in cluster 3 (Ceric.31G036600, Ceric.33G031700) were resolved by sequence similarity as previously-published *Ceratopteris* embryo genes *CrANT*^59^ and *CrLFY1*^60^ (Supplementary Fig. 15), supporting the accuracy of this cluster. Our findings thus suggest significant conservation between gene networks of the ovule/seed and post-fertilization archegonium development.

**Fig. 6:**
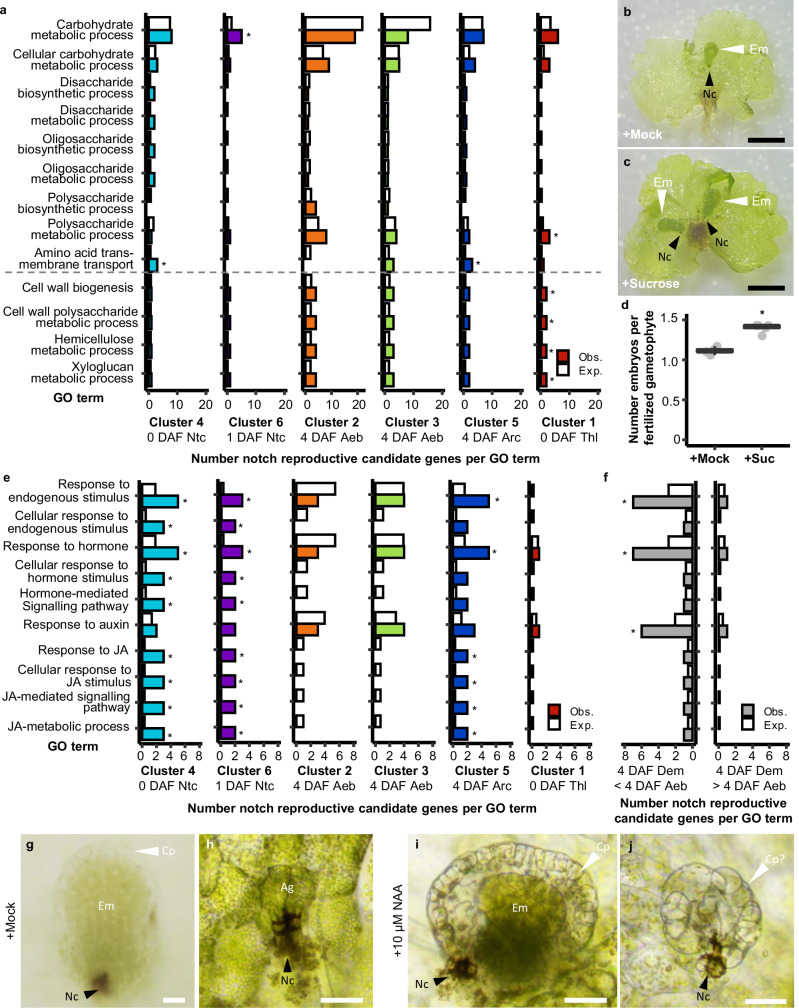
Mechanisms regulating seed-set also regulate *Ceratopteris* fertilization-dependent archegonium development. **a** Enrichment of GO terms relating to nutrients (carbohydrate metabolism and amino acid transport) vs cell wall polysaccharides within each notch expression cluster following the stages of notch development, showing the observed (“Obs.”) vs expected (“Exp.”) gene frequencies per GO term compared to the entire genome. Enrichment was tested using Fisher's Exact Test (one-sided, greater than). Asterisks denote a significant increase (*P* < 0.05) in genes with that GO term compared to that expected. **b**, **c** Gametophyte phenotypes 6 days after flooding with either mock treatment (**b**) or 2% sucrose (**c**). Mock-treated gametophytes typically carry one visible embryo but those flooded with 2% frequently carried two. **d** The mean number of visible embryos per replicate population (30 embryo-bearing gametophytes) was significantly different (*P* = 0.01) between mock and sucrose treatment. Boxplot denotes the data median and interquartile range (box) ±1.5× the interquartile range (whiskers), *n* = 5. Pairwise comparison was performed using a two-tailed Mann–Whitney test on untransformed data (Supplementary Data 6). **e**, **f** Enrichment of GO terms relating to development or hormone response within each notch expression cluster following the stages of notch development (**e**) and within genes differentially-expressed (*P*_adj_ < 0.05, log2foldchange > 1) between Aeb and Dem samples at 4DAF (**f**), showing the observed (“Obs.”) vs expected (“Exp.”) gene frequencies per GO term compared to the entire genome. Enrichment testing employed Fisher's Exact Test (one-sided, greater than). Asterisks denote a significant increase (*P* < 0.05) in genes with that GO term compared to that expected. *P*-values for GO term enrichment testing are given in Supplementary Data 5. **g–j** Brightfield microscopy of archegonia from embryo-bearing gametophytes after flooding with mock solution (**g**, **h**) or 10 µM NAA (**i**, **j**), comparing embryo-containing (**g**, **i**) and empty archegonia phenotypes (**h**, **j**). At 4 DAF under NAA treatment calyptra and embryo growth was visibly decoupled (**i**) while embryo-less archegonia of the same gametophytes exhibited ectopic cell division (**j**). Experiment repeated 3 times. Scale bars = 1 mm (black), 100 µm (white). Ag archegonium, Cp calyptra, Em embryo, Nc neck canal. Source data are provided as a **Source Data** file.

In further support of this hypothesis, our RNA-seq analysis successfully predicted two regulatory mechanisms in *Ceratopteris* archegonium development similar to seeds. During angiosperm seed development, sugar signalling via trehalose 6-phosphate (T6P) promotes seed filling and inhibits post-fertilization abortion^61–63^. In *Ceratopteris* we found significant enrichment (*P* < 0.05) of carbohydrate metabolism during notch development immediately after fertilization (cluster 6) (Fig. 6a). Disaccharide biosynthesis and metabolism GO terms were also significantly enriched (*P* < 0.05) in notch-specific candidates and genes showing differential upregulation during notch development (Supplementary Fig. 16), with the associated genes annotated as T6P synthase and T6P phosphatase orthologs (Supplementary Dataset 5). In contrast, cell wall-related carbohydrate metabolism showed more general enrichment. To test the effect of carbohydrate status on *Ceratopteris* post-fertilization development, gametophytes were flooded with a 2% sucrose solution and the effect on fertilization success compared to mock treatment (Fig. 6b, c). The proportion of the gametophyte population successfully bearing embryos was not significantly affected by sucrose (*P* = 0.5393; Supplementary Data 6) but the mean number of embryos per fertilized gametophyte was significantly increased (*p* < 0.0001; Fig. 6d). Sucrose has previously been shown to promote apogamy in *Ceratopteris* gametophytes^64^ but the additional embryos that developed here were confirmed as developing inside archegonia (Fig. 6c). Our RNA-seq analysis thus successfully predicted a role for carbohydrate status in *Ceratopteris* archegonium fertilization, which may be conserved with sugar signalling mechanisms during seed development.

Secondly, hormone response GO terms were significantly enriched prior to fertilization (cluster 4), during fertilization (cluster 6), in the 4 DAF embryoless notch (cluster 5) (Fig. 6e), and in transcripts with greater expression in Aeb tissues than the dissected embryo (Fig. 6f). JA-related GO terms were enriched in the first three of these. To test the function of JA in *Ceratopteris* reproduction, we applied exogenous methyl jasmonate (MeJA) solution at 0 DAF but found no significant effect of MeJA (*P* > 0.05) on fertilization success or embryo number (Supplementary Fig. 17). JA GO term enrichment in clusters 4 and 6 was also consistent with a role in notch meristem activity. To test if this could explain the observed enrichment, we applied MeJA to 7 day-old gametophytes (at which age the notch meristem initiates) and found that by 14 days old, MeJA-treated gametophytes had significantly fewer archegonia (*P* < 0.05; Supplementary Fig. 17) and visibly-reduced thallus size, suggesting a role for JA signalling in more general notch meristem activity. In contrast to JA, an auxin-related GO term was enriched in 4 DAF transcripts with greater expression in Aeb tissues than the dissected embryo (Fig. 6f). Auxin is a critical regulator of fertilization-dependent endosperm and seed coat development^65,66^, sufficiently so to drive parthenocarpic *Arabidopsis* seed development in the absence of fertilization^67^. To test for a role of auxin in *Ceratopteris* archegonium post-fertilization development, exogenous auxin solution was applied to gametophytes at fertilization. At 4 DAF under mock treatment growth of the embryo and archegonium calyptra (envelope) was tightly co-ordinated (Fig. 6g), whereas embryo-less archegonia showed no further development compared to pre-fertilization (Fig. 6h). Under auxin treatment embryo and calyptra growth was visibly decoupled (Fig. 6i), and embryo-less archegonia exhibited apparent ectoptic cell divisions resembling post-fertilization calyptra development (Fig. 6j). Our results thus support a function for auxin in calyptra development which may be conserved with auxin seed mechanisms.

### The ovule is enriched in *Ceratopteris* notch reproductive orthologs

**Fig. 7:**
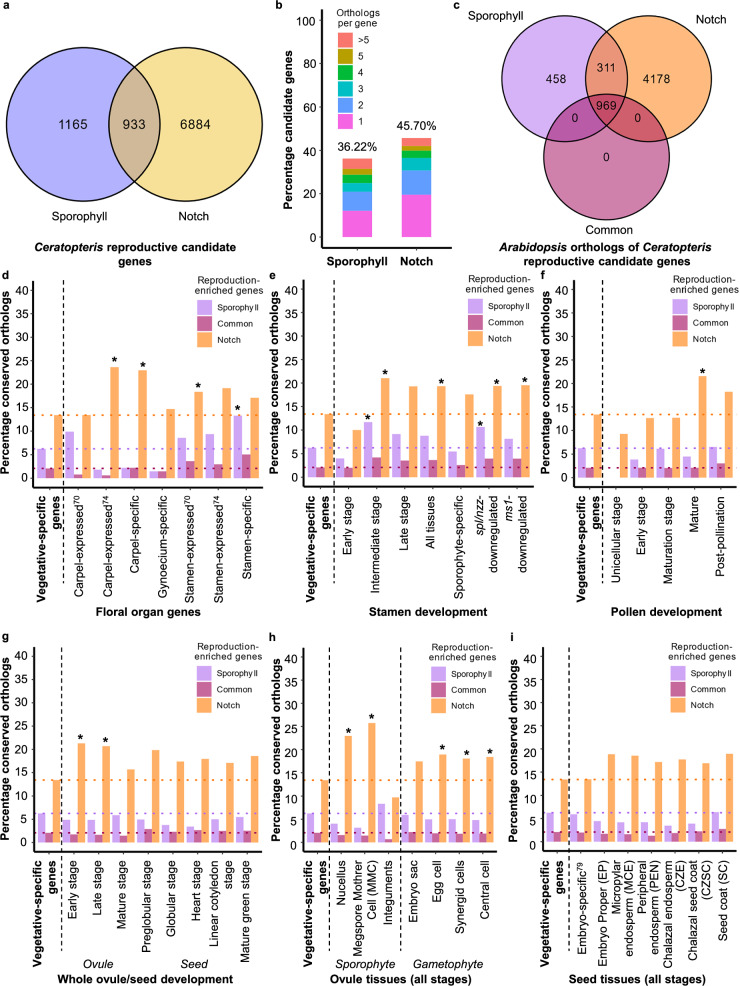
*Ceratopteris* reproductive organ gene networks are enriched within the *Arabidopsis* stamen, pollen and ovule. **a–c** Sporophyll and notch reproductive candidates are partially overlapping (**a**). All *Arabidopsis* orthologs were identified separately for candidate genes with direct orthology (**b**), of which 87.0 and 92.0%, respectively, possessed 5 *Arabidopsis* orthologs or less. Populations of nonredundant *Arabidopsis* orthologs for sporophyll, notch and common candidate genes were thus established (**c**) to enable enrichment testing against *Arabidopsis* reproductive gene datasets. **d–i** Enrichment testing of *Arabidopsis* gene orthologs for the *Ceratopteris* sporophyll-enriched, notch-enriched and common-enriched orthologs within published datasets of *Arabidopsis* reproductive genes expressed in floral organs^70,74^ (**d**), stamen development^72^ (**e**), pollen development^68,69^ (**f**), whole ovule/seed development^71,76,80^ (**g**), ovule tissues^73,75,77,78^ (**h**), and seed tissues^79,80^ (**i**) versus published vegetative-specific genes^76^. Barplots show the overlap of candidate orthologs against each target as a proportion of that dataset. Enrichment tested employed Fisher's Exact Test (one-sided, greater than), adjusted for multiple comparisons (Benjamini–Hochberg method). Asterisks denote a significant increase (*P*_adj_ < 0.05) in the proportion of overlapping genes between candidate orthologs and the target reproductive organ compared to the overlap with vegetative-specific genes. Dotted horizontal lines track the baseline overlap of each candidate ortholog dataset with the vegetative control. *P*-values for individual comparisons are given in Supplementary Data 9. Source data are provided as a **Source Data** file.

To quantify the extent of conservation between *Ceratopteris* and *Arabidopsis* reproductive gene networks, we undertook a gene orthology-based comparison. *Ceratopteris* reproductive candidates partially overlapped between the sporophyll and notch (44.5 and 11.9%, respectively) (Fig. 7a). All *Arabidopsis* gene orthologs of these candidates were identified (Fig. 7b, c, Supplementary Fig. 18, and Supplementary Data 8) and the populations of sporophyll orthologs, notch orthologs and common orthologs were each tested for enrichment within published *Arabidopsis* reproductive datasets^68–80^ compared to vegetative-specific genes^76^. Enrichment between *Ceratopteris* and *Arabidopsis* datasets occurred within reproduction-enriched candidate orthologs in the sporophyll and notch, accounted for all enrichment detected when testing all orthologs together (with the exception of notch vs embryo sac), with multiple additional significant enrichments detected (Fig. 7d–I and Supplementary Fig. 19), and the notch-enriched expression category comprised a significantly enriched proportion of candidates with *Arabidopsis* orthologs compared to the whole dataset (Supplementary Data 9). No significant enrichment was detected between *Arabidopsis* reproductive datasets and reproduction-specific or reproduction-FPKM < 1 expression categories or orthologs of candidates common between notch and sporophyll datasets (Supplementary Data 9). As such, downstream analysis focussed on the reproduction-enriched expression category.

Orthologs of sporophyll candidates were significantly enriched (*P*_adj_ < 0.05) in stamen datasets (Fig. 7d, e), but not in pollen, ovule or seed datasets (*P*_adj_ > 0.05; Fig. 7f–i). We also tested for enrichment in sporophyll and frond differentially-regulated genes (Supplementary Fig. 20), which further supported conservation between the sporophyll and stamen (*P*_adj_ < 0.05) but not the ovule (*P*_adj_ > 0.05). In contrast, notch orthologs were significantly enriched in carpel samples containing ovules (Fig. 7d) and multiple independently-derived ovule datasets: whole early (functional megaspores to 4-celled FG) and late ovules (8-celled to mature FG)^71^, nucellus and MMC genes from the premeiotic ovule^78^, and the egg, synergid and central cell of the mature FG^77^ (Fig. 7g, h). Notch orthologs also showed significant enrichment in the stamen (Fig. 7e), with both sporophyll and notch orthologs enriched in whole intermediate stamen and *spl/nzz*-downregulated datasets, representing genes relating to microsporogenesis^81,82^. Notch orthologs showed further significant enrichment in pollen, including *ms1*-downregulated stamen genes in which post-meiotic microspore development fails^83^ (Fig. 7e, f).

### Ovule genes are conserved in the post-fertilization archegonium

**Fig. 8:**
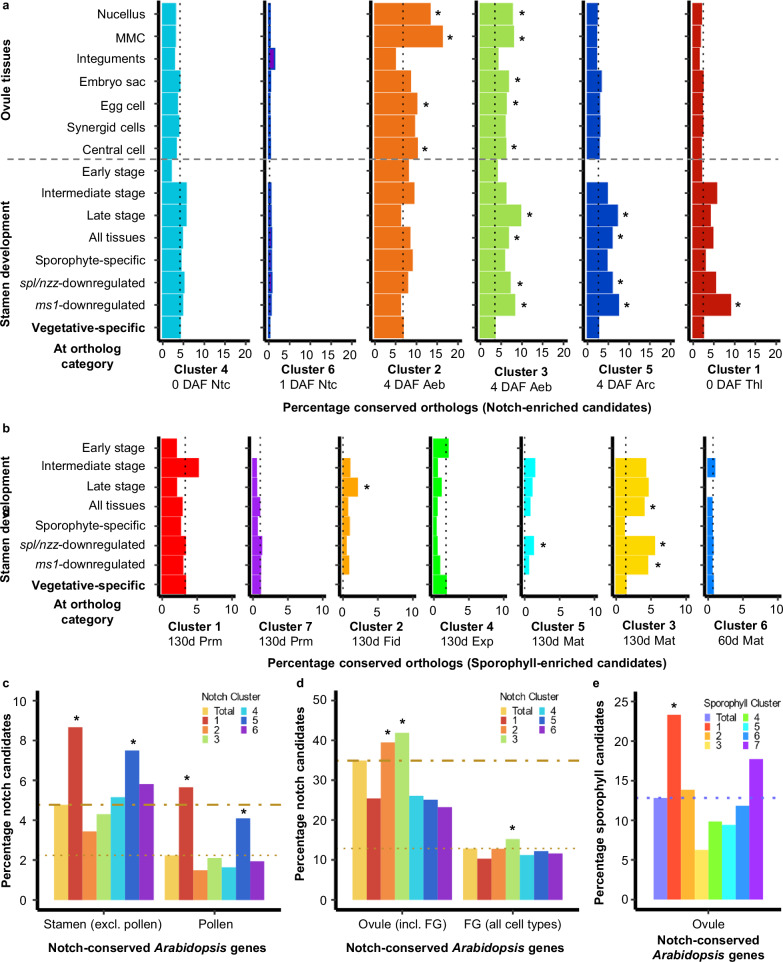
Reciprocal enrichment analysis identifies the developmental origin of conserved *Arabidopsis* reproductive gene networks in *Ceratopteris* organs. **a**, **b** Enrichment testing of *Arabidopsis* gene orthologs of *Ceratopteris* notch-enriched (**a**) and sporophyll-enriched (**b**) candidate genes by pHeatmap cluster (see Figs. 3 and 5) within published datasets of *Arabidopsis* reproductive genes expressed in the ovule and stamen. Barplots show the overlap of each *Ceratotperis* pHeatmap cluster against *Arabidopsis* reproductive datasets as a proportion of that dataset. Enrichment testing employed Fisher's Exact Test (one-sided, greater than), adjusted for multiple comparisons (Benjamini–Hochberg method). Asterisks denote a significant increase (*P*_adj_ < 0.05) in the proportion of overlapping genes between candidate orthologs and the target cluster compared to the vegetive control (dotted vertical lines). Individual *P*-values are given in Supplementary Data 9**. c–e** Reciprocal enrichment testing of *Ceratopteris* gene orthologs of *Arabidopsis* stamen (**c**) and ovule genes conserved within the notch (**d**, **e**) against pHeatmap clusters within notch (**c**, **d**) and sporophyll reproductive candidates (**e**). Barplots shows the overlap of candidate orthologs against each pHeatmap cluster as a proportion of that dataset. Enrichment testing employed Fisher's Exact Test (one-sided, greater than), adjusted for multiple comparisons (Benjamini–Hochberg method). Asterisks denote a significant increase (*P*_adj_ < 0.05) in the proportion of overlapping genes between candidate orthologs and the target cluster compared to the overlap with all sporophyll/notch reproductive candidates (dotted horizontal lines). Individual *P*-values are given in Supplementary Data 10. Source data are provided as a **Source Data** file.

To determine the developmental origin of these putative conserved networks in *Ceratopteris* reproductive organs, orthologs within each notch and sporophyll pHeatmap cluster were tested for enrichment in *Arabidopsis* reproductive genes. Notch clusters 2 and 3, both showing peak expression in the 4 DAF Aeb, were significantly enriched (*P*_adj_ < 0.05) in ovule datasets (Fig. 8a), whereas clusters 1 (0 DAF Thl), 3 (4 DAF Aeb) and 5 (4 DAF Arc) were significantly enriched in stamen datasets. Within sporophyll development, stamen orthologs were enriched in clusters 3 and 5 (mature sporophyll) and cluster 2 (fiddlehead) (Fig. 8b). We tested the robustness of these associations by reciprocal enrichment: the *Ceratopteris* orthologs of *Arabidopsis* genes from the stamen, pollen ovule and FG significantly enriched in notch, sporophyll and frond co-upregulated tests were identified (Supplementary Data 10) and their enrichment in *Ceratopteris* pHeatmap clusters tested against the overall candidate datasets. Notch-conserved *Arabidopsis* stamen genes were confirmed as reciprocally significantly enriched (*P*_adj_ < 0.05) in clusters 1 and 5 (Fig. 8c). Conserved pollen genes were enriched in the same clusters. Notch-conserved ovule genes were similarly confirmed as reciprocally enriched in notch clusters 2 and 3, with genes conserved with only the FG enriched specifically in cluster 3 (Fig. 8d). Surprisingly, notch-conserved ovule genes also demonstrated significant cross-enrichment in sporophyll cluster 1, with expression greatest in primordia (Fig. 8e), distinct from sporophyll-conserved stamen genes enriched in sporophyll cluster 3 (Supplementary Fig. 21). These cross-enriched orthologs include regulators of flowering (*FWA*)^84^, reproductive meristem development (*MRLK*)^85^, ovule specification (*BAM2*, *BAM3*)^86^ and ovule development (*BHLH11*, *AGL80*)^87,88^.

Our analyses thus suggest that gametophyte post-fertilization archegonium gene networks are conserved with the ovule (including premeiotic development), with some of these genes also conserved in sporophyll primordia. Sporophyll and notch reproductive candidates also both demonstrate significant and separate conservation with stamen sporogenous gene networks, respectively localised to late sporophyll development and gametophyte tissues distinct from the embryo-bearing archegonium.

### Conserved archegonium genes include ovule sporophytic genes

**Fig. 9:**
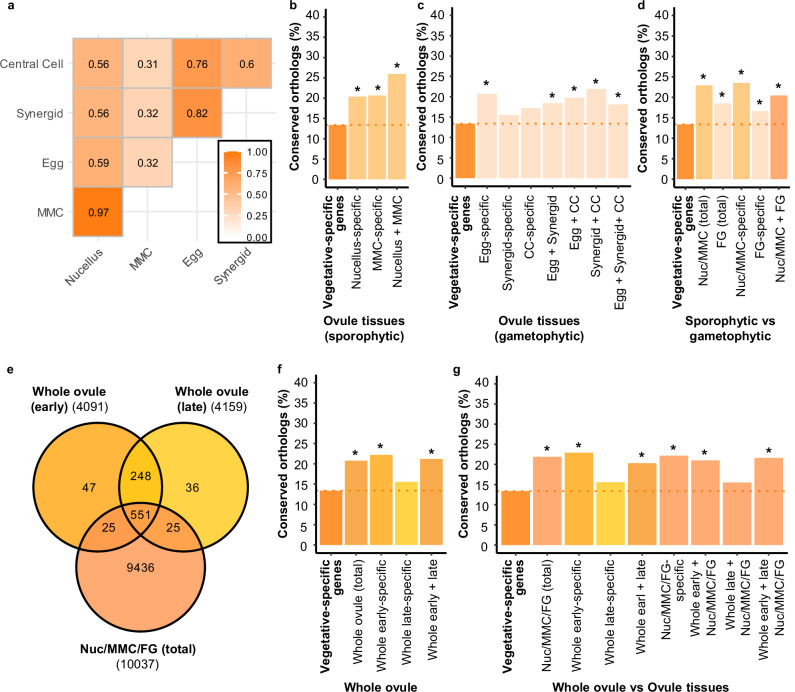
Notch candidate*s* are enriched in ovule sporophyte and gametophyte tissue-specific datasets. **a** Pairwise correlation matrix comparing the overlap in significantly-enriched notch orthologs between the different ovule tissues tested. **b–d** Enrichment testing of all notch-enriched orthologs within genes specific to or common between tissues of the premeiotic ovule (**b**), the female gametophyte (“FG”) (**c**) and both gene networks in total (**d**). “FG” represents a nonredundant summation of all the genes expressed in egg, synergid and central cell tissues^77^ where significant enrichment was identified, and “Nuc/MMC” sums the nucellus and MMC^78^ respectively. The assignment of genes to specific or shared ovule/seed tissues is shown in Supplementary Fig. 22. Barplots show the overlap of notch-enriched orthologs against *Arabidopsis* reproductive genes distributed by tissue/sample specificity as a proportion of that gene population. Dotted horizontal lines track the baseline overlap with the vegetative control, bar colours denote the tissue/sample of origin. Enrichment testing employed Fisher's Exact Test (one-sided, greater than), adjusted for multiple comparisons (Benjamini–Hochberg method). Asterisks denote a significant increase (*P*_adj_ < 0.05) in the proportion of overlapping genes between candidate orthologs and the target cluster compared to the vegetative control. **e** Overlap in *Arabidopsis* genes expressed in whole ovule stages^71,78^ and Nuc/MMC/FG samples combined. **f** Enrichment testing of all notch-enriched orthologs within genes specific to or common between whole ovule samples (**f**) and within genes specific to these or overlapping with ovule tissue datasets (**g**). Barplots show the overlap of notch-enriched orthologs against *Arabidopsis* reproductive genes distributed by tissue/sample specificity as a proportion of that gene population. Dotted horizontal lines track the baseline overlap with the vegetative control, bar colours denote the tissue/sample of origin. Enrichment testing employed Fisher's Exact Test (one-sided, greater than), adjusted for multiple comparisons (Benjamini–Hochberg method). Asterisks denote significant enrichment (*P*_adj_ < 0.05) compared to the vegetative control. Individual *P*-values are given in Supplementary Data 11. Source data are provided as a **Source Data** file.

To resolve the contribution of embryo-bearing archegonium gene networks to ovule development we tested for reproductive enrichment in gene sets specific to or common between ovule tissue and cell types (Supplementary Fig. 22). Notch orthologs significantly enriched within ovule tissues showed higher correlation within premeiotic tissues (nucellus and MMC) and mature FG tissues than between them, but still with up to 59% cross-overlap (Fig. 9a). Notch orthologs were significantly enriched (*P*_adj_ < 0.05) in nucellus-specific and MMC-specific premeiotic genes (Fig. 9b), FG cell type-specific and FG-common genes (Fig. 9c) and in genes specific to premeiotic tissues compared to the FG (Fig. 9d). We also determined that the enrichment in these tissues was distinct from that in whole early and late ovule samples, whose genes partially overlapped (Fig. 9e): notch ortholog enrichment within whole ovule samples was found to relate only to early-specific or common genes (Fig. 9f), but significant enrichment remained in gene populations absent from the whole ovule data (Fig. 9g). 627 *Arabidopsis* orthologs of notch reproductive candidates were significantly enriched specifically in the nucellus, of which 32 were also conserved with the sporophyll primordium (including *FWA, MRLK, BAM2, BAM3*; Supplementary Data 11). Within genes uniquely conserved between the nucellus and notch, we identified *HUA1* and *BEL1*, regulators of carpel and early ovule identity, respectively^89,90^. 136 FG-specific orthologs included *FERTILIZATION INDEPENDENT SEED2* (*FIS2*), which represses precocious endosperm development in the FG^91^ and 141 notch orthologs significantly enriched in all four datasets included *AtMYB65*, highly expressed in the FG central cell to also prevent endosperm division^92^ (Supplementary Data 11). Other enriched orthologs included regulators of MMC specification (*EXS/EMS1*, *DRM1*)^93,94^ and cell divisions including female meiosis (*MSH2*, *SAE2*, *RAD51C*)^53,55,56^. Significant co-enrichment (*P*_adj_ < 0.05) was also detected between notch and sporophyll orthologs enriched in *Arabidopsis* stamen sporogenous tissue (Supplementary Data 11), including the GAMYBs *MYB65* and *MYB101*^95^ and multiple auxin-responsive genes. Post-fertilization archegonium gene networks thus show significant separate and overlapping conservation with sporophytic and gametophytic tissues of the ovule, distinct from that found in the sporophyll primordium.

## Discussion

Our RNA-seq analysis identified significant genetic conservation across *Ceratopteris* and *Arabidopsis* reproductive development, consistent with the hypothesis that seed plant reproductive gene networks arose from reproductive gene networks in the last common ancestor with ancestral seedless vascular plants. Multiple predictions arising from this analysis were experimentally validated within *Ceratopteris*, and we independently identified candidate reproductive genes reported in previous individual studies (*CMADS1*^28^, *CrANT*^59^, *CrLFY*^60^). This conservation allows us to identify molecular changes between reproductive development in seedless and seed-bearing vascular plants that were potentially important to the evolution of the ovule.

GA signalling was identified as promoting the shoot sporing transition in *Ceratopteris,* similar to the shoot floral transition in angiosperms, with partially-separable roles regulating frond complexity and sporogenesis. Canonical GA signalling is inferred to have evolved in the vascular plant lineage prior to the divergence of ferns^96,97^. In lycophytes, GA promotes microspore wall development^98^ but not growth responses^97^. A role for GA in lycophyte shoot reproductive transitions has not been reported, but our data suggest this function was present at least in the last common ancestor of ferns and seed plants.

The extent of conservation between the sporing and floral transition remains to be determined. In this study, the sensitivity of our analysis was limited by the unavoidable presence of primordia within our Apx samples. However, evidence suggests not all components of the angiosperm floral transition are conserved, with Cr*LFY1* and *−2* playing no role in *Ceratopteris*^99^. Interestingly, these instead have gametophytic reproductive functions and the capacity to regulate notch meristem activity. Here, we detected further genes in the pre-fertilization notch associated with flowering and the inflorescence meristem (*TFL1-LIKE*, *SIN3-LIKE*)^50,51^. In the gymnosperm *Picea abies TFL1-like* genes are thought to lack a flowering function and instead act to promote growth cessation^100^, consistent here with the shutdown of the notch meristem. To what extent gametophytic gene networks contributed to the seed plant reproductive shoot network remains to be elucidated.

The putative *Ceratopteris* sporophyll gene network contains multiple orthologs of the angiosperm microsporogenesis programme (*EXS/EMS*, *TPD1*, *GAMYB*, *AMS1*, *MS1* and *MS2*)^49^ that results in a highly-derived three-celled male gametophyte enclosed in a sporopollenin coat^101^. Components of this network regulating sporogenesis, gametogenesis and spore/pollen wall formation are also conserved in the moss *Physcomitrium patens* (*PpMS1*, *PpMS2* and *PpGAMYB*)^98,102,103^. Our results suggest that this sporogenesis network is conserved across land plants and that the gene network in *Ceratopteris* strongly resembles that regulating microsporogenesis in seed plants, consistent with the developmental similarities reported between them^19^.

Surprisingly, the same genes relating to sporogenesis were also significantly enriched in the post-meiotic gametophyte notch. A number of these have dual roles in angiosperms: *EXS1/EMS1* and *DRM1* regulate the specification of sporogenous mother cells in both anthers and ovules^93,94,104^; *MSH2*, *SAE2*, *RAD51C* function in *Arabidopsis* male and female meiosis and also in mitosis^53,55,56^; the *GAMYB* family is essential for microsporogenesis by promoting tapetum programmed cell death (PCD)^105^ but also promotes seed germination through aleurone PCD^95^. Our findings suggest that some of these “cross-generational” genes already had functions beyond sporogenesis in the gametophyte of the last common ancestor of ferns and seed plants, and we speculate that this could have prefigured the evolution of female meiosis.

Beyond sporogenesis, *Ceratopteris* sporophyll reproductive candidate genes showed conservation with *Arabidopsis* stamen gene networks, consistent with RNA-seq analysis of *Adiantum capillus-veneris* homosporangia^34^. Gene expression changes specific to sporophyll rather than frond development were enriched in hormone-related genes, including auxin, which regulates both *Arabidopsis* early pollen development and stamen maturation^106,107^. Similarly, *Ceratopteris* auxin-related genes were associated with late sporophyll development and coincident with meiosis. A second hormone identified in sporophyll development (JA) also regulates angiosperm stamen development and, more variedly, broader aspects of floral development^108^. As such, our results support the evolution of the stamen gene network from those of ancestral sporangium-bearing sporophytic organs.

We found multiple strands of evidence that gene networks regulating post-fertilization development of the fern archegonium contributed to the origin of ovule patterning. Although conservation of FG networks between ferns and seed plants is to be expected as homologous organisms, importantly, this enrichment included genes specific to premeiotic ovules and thus cannot be explained simply by the presence of the FG in the ovule, whilst genes conversed with the angiosperm FG alone do not account for the full enrichment between ovule and post-fertilization archegonium. This putative conserved network did not include *LEC1*, previously predicted as the origin of a seed maturation network from the fern gametophyte^30^, but it is possible that further ancestral modules were co-opted later in seed evolution.

Although *Ceratoperis* sporophyll reproductive gene networks were not directly conserved with *Arabidopsis* ovule gene networks, archegonium genes conserved with the ovule were also enriched in the sporophyll primordium. This suggests that at least some part of the ovule gene network arose from an ancestral sporangium-bearing sporophytic axis, consistent with hypotheses based on morphological and fossil data^21^. This limited conservation must be considered in the context of differences observed in shoot gene networks between ferns and lycophytes^109^, raising the possibility that extant fern sporophylls do not closely resemble the ancestral spore-bearing shoot gene network from which ovules arose. Notably, however, those conserved genes identified in the sporophyll did not include all notch orthologs conserved with ovule sporophytic tissues, suggesting that part of the sporophytic network in extant ovules was gametophytic in evolutionary origin.

If current evolutionary theories are correct, can the underlying changes in development and patterning necessary to create the ovule from a sporophytic sporangiate lateral organ be explained by changes in ancestral reproductive gene networks? Our results support a model whereby that ancestral organ acquired “ovule-like” characteristics from the post-fertilization archegonium: gene networks regulating post-fertilization archegonium development were expressed in that developing organ prior to sporangium initiation, resulting in developmental modifications that began to resemble the fertilized archegonium. Consistent with this, our findings suggest this novel expression may have occurred in the primordium stage of that organ’s development. This model reconciles the morphology and fossil-based evidence of ovules evolving from modified sporangia-bearing axes^21^, the developmental genetic evidence of extant ovules as lateral organs^23^ and the observed but so-far unexplained seed-like characteristics of the gametophyte archegonium^25^.

There is precedent for large-scale ontogenetic rearrangement of gene networks that supports this hypothesised transfer from the gametophyte archegonium to a sporophytic organ. In *Physcomitrium*, gametophytic or sporophytic gene networks can be mis-expressed in the opposing generation through the mutation of regulatory protein complexes like PRC2^110^ or KNOX/BELL dimers^111^. Importantly, conservation between *Ceratopteris* and *Arabidopsis* reproductive gene networks was detected specifically in “reproduction-enriched” gene populations, suggesting that the ovule gene network arose through the repurposing of downstream targets of seedless reproductive gene networks. This model predicts that only the modifications to archegonium development associated with fertilization were expressed in the developing sporophytic axis, not the whole archegonium programme, thus allowing gametophytic reproductive development to continue without disturbance whilst modifying sporophyte organ development. Therefore, this model is also consistent with the persistence of archegonia in ancestral seed plant and extant gymnosperm ovules^5^.

## Methods

### Plant material and growth conditions

*Ceratopteris richardii* lab strain Hn-n^112^ was used for all experiments. Spores were sterilised through 15 min incubation in dilute sodium hypochlorite solution (2–3% active chlorine) containing 0.1% Tween20 (v/v), rinsed 5 times in sterile water and incubated in sterile water at room temperature under darkness for 3–4 days to imbibe^113^. Spores were sown onto 90 mm-diameter petri dishes (hereafter “plates”) containing 25 ml C-fern 1% agar medium (pH6.0) with a sterile cellophane disc between spores and media^113^. Plates were sown with a density of 200–300 spores per plate, wetted with 1 ml sterile water and sealed with micropore tape. Plates were kept humid during subsequent incubation by adding 1.5 ml sterile water at 3 and 7 days after sowing (DAS), with no water added beyond this time to prevent premature sperm release and fertilization. Fertilization was triggered at 14 DAS by flooding plates with 7.5 ml sterile water, with embryo development assumed to have begun on the day of flooding. Individual sporophytes transferred individually to fresh C-fern agar media once visible (9–11 days after flooding [DAF]). Individual sporophytes were transferred to sterile GA-7 Magenta boxes containing 100 ml C-fern 1% agar medium (pH6.0) when 2–3 fronds and elongating roots were visible (14–16 days) and wetted with 5 ml sterile water. Sporophytes were subsequently maintained in Magenta boxes until 130 days old, with sterile water re-applied whenever no surface water was visible (typically every 2–3 weeks). At 100 and 120 days 5 ml of sterile C-fern liquid media (pH6.0) was applied instead of water to maintain healthy growth. Sporophytes grown for harvesting 25 d samples were grown on 90 mm plates, transferred at 14 DAF at a density of 30–40 per plate. All plant material was grown in an MLR-352H-PE controlled environment cabinet (PHC Europe, Breda, Netherlands) at 28 °C, constant 50% relative humidity and a 16 h light/8 h dark photoperiod at a fluence of 150 μmol m^−2^ s^−1^.

### Embryo development synchronisation

To confirm the efficacy of restricted watering at synchronising embryo development, populations of gametophytes were grown with parallel growth regimes applied: either restricted watering (as described above) or “well-watered” conditions with sterile water applied at 9 and 11 DAS to maintain a constant layer of surface water, at ratio of 2 restricted water:1 well-watered plates. At 14 DAS, all well-watered plates and half of the restricted water plates were flooded with sterile water as described above (“+Flooding”), with the remaining restricted water plates left dry (“−Flooding”). One plate per treatment was sacrificed for analysis at 14 DAS (restricted-water, well-watered), 4 DAF and 7 DAF (restricted-water ±Flooding, well-watered +Flooding). From each plate 50 hermaphrodite gametophytes were selected at random and scored for the presence of an embryo using an ultraZOOM-2 stereomicroscope (GT Vision Ltd, Newmarket, UK) at 20× magnification. The experiment was replicated on four different occasions.

To characterise the timing of post-fertilization embryo development and archegonium production in response to flooding, populations of gametophytes were grown at a density of 200–300 spores per plate under restricted water conditions (as described above) and flooded at 14 DAS. At 0 (unflooded), 1, 2, 3, 4, and 7 DAF, single plates were sacrificed for analysis. From each plate, 50 hermaphrodite gametophytes were selected at random and scored for the presence of an embryo (as described above). At 1–7 DAF gametophyte selection was restricted to those with evidence of fertilization in response to flooding (brown archegonia). The experiment was replicated on five different occasions. During three of these replicates the number of archegonia per gametophyte were counted alongside scoring for the presence of an embryo. Numbers of embryo-bearing and embryo-less flooded gametophytes recorded per replicate varied due to availability within the population.

### Sporing transition characterisation

Sporophytes were grown in Magenta boxes (as described above) in populations of 25. Eleven replicate populations were grown, with populations initiated at intervals over 13 months. From 4 weeks old, the number of sporophytes with visible sporophylls within each population was recorded once per week ±1 days until 19 weeks old, by which time 100% of sporophytes had begun to spore. For nine of these replicates, the number of fronds per sporophyte was also recorded at the same time.

### Sporangium development characterisation

Single sporophylls were collected from 130 day-old (130 d) sporophytes, secured dorsal-side down to microscope slides using double-sided tape, and pinnae dissected open under a stereomicroscope using hypodermic needles as micro-scalpels to expose developing sporangia on the ventral surface. Sections of pinnae were recorded photographically to capture sporangium planar area. Sporangia were recorded at two locations per pinna (base and tip) and from two pinnae per frond, positioned basally and distally. Four sporophytes were sampled per frond developmental stage. Sporangia areas were measured from scaled micrographs using the FIJI image analysis software package^114^.

### Sporophyte hormone treatments

Sporophytes were treated with sterile 100 µM GA_3_ (0.1% EtoH, 0.01% Tween20 [v/v]) or 100 µM PBZ (0.1% EtoH, 0.01% Tween20 [v/v]) vs mock solution. For GA experiments, 14 d sporophytes were transferred to Magenta boxes (as above) containing C-fern 1% agar media with 100 µM GA_3_ or 0.1% EtOH added. Sporophytes were then treated with corresponding GA/PBZ or mock solution every 2–3 days thereafter, with 0.5 ml solution applied to the shoot tip and emerging fronds. For PBZ experiments 14 d sporophytes were transferred to Magenta boxes (as above) containing C-fern 1% agar media and treatment with PBZ or mock solution was applied (as above) at 4 weeks old and once per week thereafter. Sporophytes were grown in populations of 25 with 12/13 individuals per treatment.

### Gametophyte experimental treatments

For analysis of post-fertilization development, plates of gametophytes were grown to 14 DAS as described above. For sucrose experiments plates were flooded with 7.5 ml sterile 2% sucrose (w/v) or water and incubated under normal growing conditions for 7 days. For JA experiments plates were flooded with 7.5 ml sterile 100 µM MeJA or mock solution (0.00002% EtOH [V/V]) and then incubated under normal growing conditions for 7 days. At 7 DAF, 30 (sucrose) or 50 (JA) hermaphrodite gametophytes from each plate were randomly selected and scored for the presence of a visible embryo at 2× magnification using a dissecting microscope and the number of embryos counted. If <30/<50 embryo-bearing gametophytes were collected, further embryo-bearing gametophytes were subsequently screened, and the number of embryos counted. For auxin experiments plates were flooded with 7.5 ml sterile 10 µM NAA or mock solution (0.1 µM NaOH), then incubated under normal growing conditions for 4 days. Gametophytes were imaged under brightfield microscopy.

For analysis of notch meristem activity, plates of gametophytes were grown to 7 DAS and then transferred to fresh C-fern 1% agar media containing 100 µM MeJA or mock treatment (0.00002% EtOH [v/v]), and then wetted with 1.5 ml of the corresponding sterile JA or mock solution (as above). Plates were then incubated under normal growing conditions. At 15 DAS, 20 hermaphrodite gametophytes were randomly selected from each plate and the number of visible archegonia scored at 2× magnification using a dissecting microscope.

### mRNA-seq sample preparation

Tissue samples for mRNA-seq were harvested by dissection and immediately frozen in liquid nitrogen. Sequential stages of sporophyte frond development (shoot apex [Apx], frond primordium [Prm], frond fiddlehead [Fid], expanding frond [Exp] and mature frond [Mat], see Fig. 1) were harvested from 60 d sporophytes (vegetative phase) and 130 d sporophytes (reproductive phase) within 2 h per population grown, with replicate populations harvested between 5 and 10 h after dawn (ZT5-ZT10). Frond stages were determined by eye and populations were harvested on separate occasions to prevent confusion of similar-looking stages between 60 and 130 d. Dissection of Apx and Prm samples was performed under a stereomicroscope using watchmaker’s forceps. Tissues were frozen immediately after dissection by collection into tubes floating on liquid nitrogen. All biological replicates comprised tissue from at least 4 independent sporophytes (Supplementary Data 12) from one (all Mat, Exp, Fid, 60 d Apx and Prm), 2–3 (130 d Apx and Prm) or 3–4 independently-harvested populations (25 d Apx and Prm), respectively. Exp samples were designed to encompass “early” (unfurling), “mid“ (unfurled to 50% final size) and “late“ frond expansion (50–75% final size) within each biological replicate (Supplementary Fig. 4). Samples across frond development at 60 and 130 d comprised tissue from the same harvested population(s) per biological replicate.

Gametophyte stages were necessarily harvested on separate days, individual populations harvested within 2–4 h with replicate populations harvested between 5 and 10 h after dawn (ZT5-ZT10). Dissection of the notch was performed under a stereomicroscope using hypodermic needles as micro-scalpels, the developmental status of each individual confirmed by eye. Each biological replicate comprised notch or thallus tissue from 150 to 225 individual gametophytes (Supplementary Data 12) pooled from 1 (0 DAF, 1 DAF, 2DAF), 2-3 (4 DAF) or 3-4 independently-harvested populations (Dem, 761–788 embryos per sample), respectively. Notch and thallus from the same individuals remained paired when preparing biological replicates for sequencing.

RNA was extracted using the Qiagen RNeasy plant mini kit with on-column DNase treatment (QIAGEN, Hilden, Germany). RNA quality was determined using a 2100 Bioanalyser RNA 6000 nano assay (Agilent Technologies, Santa Clara, USA). Individual sample RIN values are supplied in Supplementary Data 12. Library preparation, 150 bp paired-end Illumina sequencing was performed separately for sporophyte and gametophyte samples by Novogene (Novogene UK, Cambridge, UK) to a depth of ≥ 20 million reads per sample. QC data is provided in Supplementary Data 12.

### Imaging

Whole sporophytes and gametophyte plates were photographed using a Cybershot DSC-HXTV digital camera (Sony Corporation, Tokyo, Japan). Individual gametophytes and dissected tissue samples were photographed using an ultraZOOM-2 stereomicroscope with an attached GXCAM-U3PRO-X digital camera and GXCam software (GT Vision Ltd, Newmarket, UK). Photographs were minimally processed by adjusting brightness and contrast and sharpness for whole images.

### Statistics and reproducibility

No statistical method was used to predetermine sample size. Randomization was used during the growing of plant material for each experiment. Sporophyte populations under tissue culture were grown in a 5 × 5 grid pattern, with the positions of Magenta boxes randomised once per week. Similarly, the positions of gametophyte plates growing within an experiment was randomised once per week. For gametophyte characterisation experiments, individuals were selected for scoring at random within each plate, with the exception of when additional embryo-bearing gametophytes were required. For gametophyte sucrose, auxin and JA response experiments, the investigators were blinded to treatment allocation until after data collection was complete. For sporophyte GA and PBZ response experiments, the requirement to apply treatments repeatedly and the clear growth phenotypes exhibited before completion of the experiment made blinding an impracticable approach.

Statistical analyses of characterization experiments were conducted in R version 4.2.2 using the car package^115^. Pairwise comparisons were performed using two-tailed students *T*-tests or Mann–Whitney nonparametric tests (as shown within each figure), following analysis of the data structure using Shapiro–Wilk for Normal distribution^116^ and Levene’s Test for equal variance^117^ to determine what test was appropriate. Where necessary data transformation was applied (indicated within each figure legend). Datasets were assessed for significant outlying datapoints, but it was not necessary to exclude any data from the analyses performed. A post-hoc correction for multiple comparisons was applied to *P*-values using the Benjamini–Hochberg method^118^.

### mRNA-seq data analysis

Library preparation and sequencing were conducted by Novogene using NovaSeq 6000 PE150. We obtained an average of over 56 million pair reads per samples, with each sample covered by at least by 30 million paired reads. We sequenced three biological samples (replicates) for each developmental stage analysed. FAST QC (v.0.11.5) was used to assess the quality of the sequenced libraires. Reads were aligned to the *Ceratopteris richardii* genome v2.1^36^ using HISAT2 (v.2.2.1)^119^. SAMtools (v.1.10)^120^ was used to sort and index Sam files into Bam format. Read counts were generated using HTSeq (v.0.13.5)^121^. Read counts per gene were analysed in R using DEseq2 (v.1.34.0)^122^. Differentially expressed genes (DEGs) were computed in pairwise comparisons across all developmental stages (each composed by three biological samples). We considered DEG any gene with an adjusted *p*-value (*P*_adj_) below 0.05 and a log2 foldchange > 1. For each gene, the transcripts per million reads) was computed in R starting from the raw read count.

Orthology of *Ceratopteris* genes to *Arabidopsis* was identified using Orthofinder^123^ with default settings against the following proteomes: *Adiantium capillus-veneris*^34^, *Alsophila spinulosa* (v3.1)^35^, *Arabidopsis thaliana* (TAIR10)^124^, *Amborella trichopoda* (v1.0)^125^, *Cycas panzhihuaensis*^126^, *Gnetum montanum*^127^, *Glycine max* (Wm82.a4.v1)^128^, *Lycopodium clavatum*^129^, *Manihot esculenta* (v8.1) (https://phytozome-next.jgi.doe.gov/info/Mesculenta_v8_1), *Marsilea vestita* (v3.0)^130^, *Micromonas pusilla* CCMP1545 (v3.0)^131^, *Ostreococcus lucimarinus* (v2.0)^132^, *Oryza sativa* (v7.0)^133^, *Physcomitrium patens* (v3.3)^134^, *Quercus robor* (PM1N)^135^, *Solanum lycopersicum* (ITAG4.0)^136^, *Selaginella moellendorffii* (v1.0)^137^, *Spirodela polyrhiza* (v2)^138^, *Thuja plicata* (v3.1)^139^, and *Volvox carteri* (v2.1)^140^. Orthology data is provided in Supplementary Data 8. Additional GO term annotations were assigned from gene orthologs in the model species *Arabidopsis*, *Oryza*, *Selaginella, Physcomitrium* and *Marchantia*. Higher-order Venn comparisons were performed in Interactivenn^141^. Further analysis of transcriptome data was performed in R, with cluster analysis using the pHeatmap package and GO term enrichment testing using the TopGO package employing Fisher’s Exact Test (classic algorithm). *Arabidopsis* reproductive gene datasets were obtained from past publications and filtered against published vegetative-specific genes (Supplementary Data 9). Enrichment testing of *Arabidopsis* orthologs of *Ceratopteris* genes against these datasets was performed in R using Fisher’s Exact Test (one-sided, “greater than”) and corrected for multiple comparisons using the Benjamini–Hochberg method^118^. All other plots were prepared in R using the ggplot2^142^ and the ggvenn and ggcorrplot packages.

### Reporting summary

Further information on research design is available in the Nature Portfolio Reporting Summary linked to this article.

## Supplementary information

Supplementary Information

Supplementary Data 1

Supplementary Data 2

Supplementary Data 3

Supplementary Data 4

Supplementary Data 5

Supplementary Data 6

Supplementary Data 7

Supplementary Data 8

Supplementary Data 9

Supplementary Data 10

Supplementary Data 11

Supplementary Data 12

Reporting Summary

Transparent Peer Review file

## Source data

Source Data

## Data Availability

The raw sequencing read data generated in this study have been deposited in the NCBI Gene Expression Omnibus (GEO) database under accession code GSE291236. The processed gene expression data are provided in the Source Data file. The statistical analyses generated in this study are provided in the Supplementary Information. The published *Arabidopsis* microarray expression data used in this study are available at the NCBI GEO under accession codes GSE1265^70^, GSE1266^70^, GSE1267^70^, GSE1268^70^, GSE1269^70^, GSE1270^70^, GSE1271^70^, GSE1272^70^, GSE1273^70^, GSE1274^70^, GSE1275^70^, GSE8864^72^, GSE680^76^, GSE12404^80^; the EMBL-EBI Array Express database under accession codes E-MEXP-1246^73^, EMEXP-1920 [https://www.ebi.ac.uk/biostudies/ArrayExpress/studies/E-MEXP-1920]^75^, E-MEXP-3137 MMC [https://www.ebi.ac.uk/biostudies/ArrayExpress/studies/E-MEXP-3137]^78^, E-MEXP-3138^78^; or online supplementary material^68,69,71,74,77,79^. The processed *Arabidopsis* gene datasets used in this study are provided in the Supplementary Information. Source data are provided with this paper.
