# Supplementary material for: The post-fertilization archegonium gene network of seedless plants contributed to the origin of seeds
Source: Nat Commun. 2026 Jun 19;17:7751. doi: 10.1038/s41467-026-74577-w (PMC13434465; doi:10.1038/s41467-026-74577-w)
Supplement: Supplementary file 1 [file 41467_2026_74577_MOESM14_ESM.pdf]

nature portfolio

Corresponding author(s): Andrew R.G. Plackett

Last updated by author(s): Jan 25, 2026

Reporting Summary

Nature Portfolio wishes to improve the reproducibility of the work that we publish. This form provides structure for consistency and transparency in reporting. For further information on Nature Portfolio policies, see our Editorial Policies and the Editorial Policy Checklist.

Statistics

For all statistical analyses, confirm that the following items are present in the figure legend, table legend, main text, or Methods section.

n/a	Confirmed	
☐	☒	The exact sample size (*n*) for each experimental group/condition, given as a discrete number and unit of measurement
☐	☒	A statement on whether measurements were taken from distinct samples or whether the same sample was measured repeatedly
☐	☒	The statistical test(s) used AND whether they are one- or two-sided<br>*Only common tests should be described solely by name; describe more complex techniques in the Methods section.*
☒	☐	A description of all covariates tested
☐	☒	A description of any assumptions or corrections, such as tests of normality and adjustment for multiple comparisons
☐	☒	A full description of the statistical parameters including central tendency (e.g. means) or other basic estimates (e.g. regression coefficient) AND variation (e.g. standard deviation) or associated estimates of uncertainty (e.g. confidence intervals)
☐	☒	For null hypothesis testing, the test statistic (e.g. *F*, *t*, *r*) with confidence intervals, effect sizes, degrees of freedom and *P* value noted<br>*Give P values as exact values whenever suitable.*
☒	☐	For Bayesian analysis, information on the choice of priors and Markov chain Monte Carlo settings
☒	☐	For hierarchical and complex designs, identification of the appropriate level for tests and full reporting of outcomes
☒	☐	Estimates of effect sizes (e.g. Cohen's *d*, Pearson's *r*), indicating how they were calculated

Our web collection on statistics for biologists contains articles on many of the points above.

Software and code

Policy information about availability of computer code

Data collection	Next generation sequencing (mRNA-seq) was conducted by Novogene using NovaSeq 6000 Paired-End 150bp sequencing using Illumina proprietary software.
Data analysis	FAST QC (v.0.11.5) was used to assess the quality of the sequenced libraries (Andrew et al. 2010). Reads were aligned to the Ceratopteris richardii genome v2.1 (Marchant et al. 2021) using HISAT2 (v.2.2.1) (Kim et al. 2019). SAMtools (v.1.10) (Li et al., 2009) was used to sort and index Sam files into Bam format. Read counts were generated using HTSeq (v.0.13.5) (Anders et al., 2015). The read counts per gene were analysed in R using DEseq2 (v.1.34.0) (Love et al., 2014).<br><br>Orthology of Ceratopteris genes to Arabidopsis was identified using Orthofinder (Emms and Kelly, 2019) with default settings. Higher-order Venn comparisons were performed in Interactivenn (Heberle et al., 2015).<br><br>Further analysis of transcriptome data was performed in R, with cluster analysis using the pHeatmap package (Kolde, 2018) and GO term enrichment testing using the TopGO package (Alexa and Rahnenfuhrer, 2024), employing Fisher's Exact Test.

For manuscripts utilizing custom algorithms or software that are central to the research but not yet described in published literature, software must be made available to editors and reviewers. We strongly encourage code deposition in a community repository (e.g. GitHub). See the Nature Portfolio guidelines for submitting code & software for further information.

Data

Policy information about availability of data

All manuscripts must include a data availability statement. This statement should provide the following information, where applicable:

- Accession codes, unique identifiers, or web links for publicly available datasets
- A description of any restrictions on data availability
- For clinical datasets or third party data, please ensure that the statement adheres to our policy

Raw sequencing read data is available via the NCBI Gene Expression Omnibus (GEO) data repository (reference GSE291236). Processed data and statistical analyses from the experiments presented here are provided in online supplemental information. Source data are provided with this paper.

Research involving human participants, their data, or biological material

Policy information about studies with human participants or human data. See also policy information about sex, gender (identity/presentation), and sexual orientation and race, ethnicity and racism.

Reporting on sex and gender	N/A
Reporting on race, ethnicity, or other socially relevant groupings	N/A
Population characteristics	N/A
Recruitment	N/A
Ethics oversight	N/A

Note that full information on the approval of the study protocol must also be provided in the manuscript.

Field-specific reporting

Please select the one below that is the best fit for your research. If you are not sure, read the appropriate sections before making your selection.

[x] Life sciences [ ] Behavioural & social sciences [ ] Ecological, evolutionary & environmental sciences

For a reference copy of the document with all sections, see nature.com/documents/nr-reporting-summary-flat.pdf

Life sciences study design

All studies must disclose on these points even when the disclosure is negative.

Sample size

For spoprhyte mRNA-seq all biological replicates comprised tissue from at least 4 independent sporophytes per population harvested from one (Mat, Exp, Fid, 60d Apx and Prm), 2-3 (130d Apx and Prm) or 3-4 independently-harvested populations (25d Apx and Prm), respectively. For gametophyte mRNA-seq each biological replicate comprised notch or thallus tissue from 150-225 individual gametophytes pooled from 1 (0 DAF, 1 DAF, 2DAF), 2-3 (4 DAF) or 3-4 independently-harvested populations (Dem, 761-788 embryos per sample) respectively.

For sporophyte developmental characterisation experimental populations of 25 plants were growth, which was constrained by the availability of growth space. For sporophyte hormone response experiments 12-13 plants were assigned to each treatment group per experimental population (half the population that could be grown), with the proportion reversed in the next replicate grown.

For gametophyte characterization and sucrose/hormone response populations of 30 or 50 randomly-selected individuals (from an average population of 80-100) were used per experimental replicate.

No sample size calculation was performed. In all growth responses tested the variability between individuals within a population was found to be small.

Data exclusions

No data exclusions were used.

Replication

All mRNA-seq experiments included three biogially-independent replicates sequenced per sample, following the conventionally accepted minimum. Replicate populations included in the biological replicates were harvested between 5 and 10 hours after dawn (ZT5-ZT10), requiring 2 hours (sporophyte) or 2-4 hours (gametophyte) per replicate. Each biological replicate sequenced included each stage of frond development from the same population(s), thus minimising bias in the experimental results. Populations were not re-used between biological replicates.

For sporophyte experiments the following numbers of independent populations were were grown: 11 (sporing characterisation), 3 (GA response), 3 (PBZ response).

For gametophyte experiments a minimum of 3 experimental replicates were performed (JA gametophyte growth), and for all other treatments 5 experimental replicates were performed.

	ANOVA was used to determine if there was a significant difference between experimental replicates. ANOVA results are reported in supplementary data.
Randomization	For sporophyte mRNA-seq sampling individuals were selected to harvest for individual frond stages based on the developmental stage of the most recent frond to emerge, to ensure that each frond stage was collected from each population grown. With the exception of Apx and Prm samples, where all individuals within a population were harvested from, for all other stages of frond development re-sampling of plants between stages was avoided.<br><br>For gametophyte mRNA-seq sampling Notch and thallus from the same individuals remained paired when preparing biological replicates for sequencing.<br><br>For sporophyte treatment experiments individuals were assigned to treatment groups randomly, and grown in a random design within the CE chamber. The location of individuals within the design was re-randomised once per week to avoid environmental bias.<br><br>For gametophyte treatment experiments treatment were assigned to populations (agar plates) by pairing plates based on population density and then assigning one of each pair to either the mock or experimental treatment, to control for variable population density. Individuals from the population were selected for experiments at random using forceps and a dissecting microscope, ensuring that individuals were taken from >3 locations spaced around the growth surface.
Blinding	For sporophyte hormone treatment experiments, because of the long-term nature of these with the need to repeatedly and regularly re-apply hormone treatments, making fresh treatment solutions each time, together with the pronounced morphological effect on plant growth from very soon after treatment, it was not technically feasible or effective to anonymise treatments.<br><br>For gametophyte treatment experiments, after application of experimental treatments plates were anonymised using randomly-assigned letter codes. Experimental data was collected from anonymised plates 4-7 days later in letter code order, and treatments subsequently de-anonymised at the data analysis stage.

Reporting for specific materials, systems and methods

We require information from authors about some types of materials, experimental systems and methods used in many studies. Here, indicate whether each material, system or method listed is relevant to your study. If you are not sure if a list item applies to your research, read the appropriate section before selecting a response.

Materials & experimental systems

n/a	Involved in the study
☒	☐ Antibodies
☒	☐ Eukaryotic cell lines
☒	☐ Palaeontology and archaeology
☒	☐ Animals and other organisms
☒	☐ Clinical data
☒	☐ Dual use research of concern
☐	☒ Plants

Methods

n/a	Involved in the study
☒	☐ ChIP-seq
☒	☐ Flow cytometry
☒	☐ MRI-based neuroimaging

Dual use research of concern

Policy information about dual use research of concern

Hazards

Could the accidental, deliberate or reckless misuse of agents or technologies generated in the work, or the application of information presented in the manuscript, pose a threat to:

No	Yes
☒	☐ Public health
☒	☐ National security
☒	☐ Crops and/or livestock
☒	☐ Ecosystems
☒	☐ Any other significant area

Experiments of concern

Does the work involve any of these experiments of concern:

No	Yes	
☒	☐	Demonstrate how to render a vaccine ineffective
☒	☐	Confer resistance to therapeutically useful antibiotics or antiviral agents
☒	☐	Enhance the virulence of a pathogen or render a nonpathogen virulent
☒	☐	Increase transmissibility of a pathogen
☒	☐	Alter the host range of a pathogen
☒	☐	Enable evasion of diagnostic/detection modalities
☒	☐	Enable the weaponization of a biological agent or toxin
☒	☐	Any other potentially harmful combination of experiments and agents

Plants

Seed stocks

Ceratopteris richardii wild-type laboratory strain Hn-n (Warne & Hickok, 1987)

Novel plant genotypes

No novel plant genotypes produced.

Authentication

mRNA-seq data was aligned against the Ceratopteris richardii Hn-n reference genome (Marchant et al. 2022). For sporoprhyte samples 87-92% of reads mappedper sample, for gametophyte samples 83-89% of reads mapped per sample. Percentage mapping for each sample is provided in supplementary data 11.
