# Supplementary material for: The post-fertilization archegonium gene network of seedless plants contributed to the origin of seeds
Source: Nat Commun. 2026 Jun 19;17:7751. doi: 10.1038/s41467-026-74577-w (PMC13434465; doi:10.1038/s41467-026-74577-w)
Supplement: Supplementary file 2 [file 41467_2026_74577_MOESM15_ESM.pdf]

nature portfolio

Peer Review File

The post-fertilization archegonium gene network of seedless plants contributed to the origin of seeds

Corresponding Author: Dr Andrew Plackett

This file contains all reviewer reports in order by version, followed by all author rebuttals in order by version.

Version 0:

Reviewer comments:

Reviewer #1

(Remarks to the Author)
This work by Plackett and Catoni aimed to trace the origin of seed by generating and examining a comprehensive gene expression atlas for the fern Ceratopteris richardii. This is a monumental work, combining detailed characterizations of developmental progression and comparisons of gene expression across tissues and species. Importantly, a few follow-up experiments were conducted to test the hypotheses resulted from the RNA-seq data. The results are interesting, and would definitely lead to further research to test the various hypotheses raised in this study.

My overall take is that the authors are asking very important questions, using well-designed experiments and thorough data analyses.
That being said, the manuscript is complex and, to me, difficult to digest at places. The writing can be tightened up more. In addition, without further gene functional studies, one cannot conclusively link the genetic networks between ferns and Arabidopsis. For the most part the authors are cautious with the wording, but not consistently.

A few detailed comments/suggestions below:
Line 30-33. These two first sentences in the abstract have very similar structure "… how… remained…" and should probably be revised.

Line 54. I'm not a big fan of the use of "monilophytes". I think it's an unnecessary jargon; just refer them as "ferns".

Line 82. "retained within early seeds and gymnosperms". Something is missing here.

Line 167-169, 171-172. The authors expected the key genes to be differentially expressed between apex and primordia, but my understanding is that their Apx samples actually contained early primordia as well. I am not sure if such comparison is reasonable.

Line 176. GA 20-oxidase is part of a larger gene family (cytochrome P450). It would be necessary to present a gene phylogeny to demonstrate this candidate is in fact an ortholog of GA 20ox.

Line 182. The phenotype from GA-treated plants is very interesting and exciting. I wonder if the authors could further interrogate the role of GA by using GA biosynthesis inhibitors. There are many inhibitors that work on different part of the pathway.

Line 233-237. This sentence is too long and apparently missing some words. I cannot quite follow it.

Line 291. "Associated genes were mostly related to auxin". But in Fig. 6, I could see many JA related GO terms. I wonder if the authors have looked into JA and why JA wasn't highlighted in the text.

Line 295-298. So auxin treatment changed phenotypes of archegonia, but what does it mean? Does this finding support anything?

Line 390. I think "unambiguously" is too strong here.

Line 422-425. I might have missed something but I couldn't see the link between "homosporous meiotic gene networks" and "seed plant microsporogenesis".

Line 578-580. It is important to state exactly how novogene carried out read-mapping, assembly, and transcript quantification and annotation.

Supplementary Fig. 5. The image resolution for PCA plots is too low.

Reviewer #2

(Remarks to the Author)
The manuscript entitled "The post-fertilization archegonium gene networks of seedless plants contributed to the origin of the seed" aims to explore the molecular origin of the ovule in seed plants by analyzing into the gene networks of fern sporogenesis and archegonia development.

I want to start this review by saying that I greatly enjoy this topic and understanding the evolution of reproductive structures in plants is a topic with broad interest. I think this work is worthwhile and the questions are important to explore. I find the work of the authors to be extremely robust, rigorous, and heroic in scale—there is a lot of data here.

However, I have many issues with the argument, hypotheses proposed, and evolutionary/developmental explanations. While I do not think the manuscript or the hypotheses in their current state should move forward, I hope that the authors can view my review as constructive and not destructive, as that is my intention.

First, the authors present a molecular-level hypothesis for the origin of the seed, proposing that "mis-expression" of archegonium gene networks played a key role in the origin of the ovule. This hypothesis appears to stem from correlations of gene networks in homologous structures (e.g., developing egg-bearing structures in ferns and ovules, which are developing egg-bearing structures in seed plants). As the authors know and explain, in seed plants sporangia are modified into the nucellus (with some modification and addition of other maternal tissue), within which a single surviving meiotic product gives rise to the female gametophyte and subsequently the egg-bearing structure. Thus, this paper, while a heroic amount of work, seems to simply demonstrate that there are similar gene networks in homologous structures (e.g., some parts of the ovule and some parts of the egg-bearing portion of the developing fern gametophyte). If the canonical evolutionary process is true, then the evolution of the ovule is brought about by the retention of the gametophyte within the sporangium and this would be borne on the mother sporophyte. In this case, it is axiomatic that egg-producing gene expression in seedless plants would be observed in egg-producing structures of seed plants. Put another way, the gene networks in fern archegonia (and during embryogenesis) should inherently mirror those of seed plants because ovules are essentially archegonia (or egg-bearing structures) enveloped in maternal tissue. These genetic correlations highlight that egg-bearing structures across plants are homologous and exhibit similar gene networks without providing deeper evolutionary insights. Moreover, the specifics of this hypothesis are also tenuous, and they are supported by similarities in sugar transport and auxin signaling genes. These processes are ubiquitous in all plant organs and their similar expression between archegonia and ovules is unconvincing.

Zooming out for a moment, the authors' assertion that ovules arose from fertilized archegonium gene networks also lacks clarity overall clarity. Perhaps I do not understand the processes here but what does this hypothesis even mean from a developmental or evolutionary perspective? How exactly does the heterotopy of archegonial developmental genes (or embryogenesis gene networks) lead to the evolution of the ovule? This needs to be fleshed out in much more detail.

The lack of clarity around the hypothesis is also furthered by other connected but tangential hypotheses laid out in the discussion. For instance, line 427 the authors make a statement about the origin of heterospory, however the main claim elsewhere is about ovule development not the heterospory. In this same vein, in line 413 the authors indicate that floral and sporophyll reproductive networks may have arisen from ancestral genetic networks. Again, anthers and fern sporophylls would be homologous structures (structures bearing sporangia). In this case it is not only separate from their primary claim, but also it seems like the authors are observing that microsporangia in seed plants and sporangia in homosporous ferns express similar gene networks, which is interesting, but not necessarily surprising. Rather than providing new frameworks or hypotheses this latter point supports the hypothesis that anthers may be homologous to spore-bearing structures in ferns.

Another important point is that it would be much more poignant to compare ovule development with megasporophyll development in a heterosporous fern or lycophytes, not homosporous ferns. The fact that the authors only found that archegonia-related genes are related to ovule genes and not sporophyll gene is not surprising and I wonder if a comparison between megasporophyll development would be more likely to yield interesting results.

Importantly, this work would be enhanced by invoking developmental processes such as heterotopy or heterochrony, if that is what the authors mean by "repurposing ancestral reproductive gene networks." However, I would argue that it is not "repurposing" as the genes are still being expressed in homologous structures (e.g., egg producing structures in both ferns and seed plants), so I don't really understand what the new hypothesis is.

Finally, the authors should refine their comparisons between homologous structures (lines 409–413) and provide greater specificity regarding developmental processes invoked in lines 424–425. Overall, while the topic is intriguing, the

hypotheses and the paper require significant clarification and refinement.

I also will say that most of the figures and data generated and presented, while interesting, do not bear on the main claim of the paper. Methodologically, it may also be important to run a differential gene expression analysis using deseq2 or masigpro across developmental stages.

Furthermore, specific line edits are necessary to streamline the writing. Please see below.

I want to end where I started and say that I do believe this work is exciting and important. However, I think the claims proposed by the authors are somewhat unclear from a developmental and evolutionary perspective and I further do not think the data the authors generated support their claims (in the way I perceive them).

Line edits

30-32: two back to back statements of unsolved questions could be streamlined.

57: It is important to caution against these claims that the seed and the flower are the major dominant trait promoting seed/flowering plant diversification. The Acrogymnosperms are quite species poor and many of the sister group angiosperms (e.g., ANA grade) are also quite species poor. Neither the seed nor the flower alone likely is responsible for the major diversity of flowering plants—rather it is a few hypoer diverse groups of flowering plants that contribute mostly to the impressive diversity of the group. This has to do with unique floral morphologies and pollinator interactions (O'Meara et al., 2016 Proc B.).

Line 57-59: The body plan of the ovule is no different than the hypothetical scenario of a fern maintaining a single endorsporically developed megagametophyte on the parent sporophyte plant. The ovule is of course an interesting evolutionary innovation, but it is inherited from previous structures with clear holomogies.

Line 85-86: redundant use of pollen and embryo sac

Line 390: I am unsure about this claim.

Line 397: the authors indicate "but three Ceratopteris homologs were identified with expression patterns consistent with downstream targets of the sporing transition. This could indicate that the GA-MADS module of angiosperm shoots was co-opted from an ancestral mechanism in the vascular plant shoot reproductive transition." To confirm this the authors would want to build gene trees and observe the phylogenetic placement of the MADS-box genes expressed in Ceratopteris and those expressed in developing ovules. But moreover, what are the authors even implying from this statement? What about the MADS module in fern reproduction would have been co-opted for shoot reproductive transition? Clarity around the actual developmental shifts and what they mean are important to explain.

Line 409-413: these are homologous structures (structures bearing sporangia). It seems like the authors are just observing that microsporangia in seed plants and sporangia in ferns express similar gene networks.

Line 416-417: this is somewhat confusing, perhaps it is just syntactic. From reading this it seems like the authors are saying that SGO and ZIP4 are produced in the developing sporophyte and also the gametophytic notch, but what do the authors mean by post-meiotic? Do you mean that the entire gametophyte is post meiotic (e.g., derived from a spore that is the product of meiosis?). If so, it just reads a bit odd given that the entire gametophyte of any plant (seedless or seeded) is post meiotic.

Line 424-425: what do the authors mean when they say "female-specific meiosis mechanisms could have diverged by co-opting pre-existing notch gene networks already utilising meiosis genes." What exactly is the developmental process here? I believe this is a case of invoking causation from genetic correlations. It also may be more important to look at heterosporous ferns or lycophytes and compare the development of megasporophylls with ovule development.

Line 437: More than embryogenesis what are the "striking functional similarities between the post-fertilization fern archegonium and the seed" ?

Fig 1. How could the authors tell between fertile and sterile leaf primordia. Figure g/m look awfully similar.

Methods:

Line 470: no capitalization after period "individual"

Line 532: what are the different frond developmental stages?

Line 582: Glycine not italicized

Line 584: seems like this analysis would benefit from having heterosporous fern genomes in it.

Reviewer #3

(Remarks to the Author)
The evolution of seeds from previously spore-bearing plants is enigmatic. In this paper the authors analysed the network of seed genes in the non-seed plant Ceratopteris based on novel RNA-seq data of reproductive stages. The idea behind is was to determine how the seed-development network evolved. They find that seed genes are enriched specifically in the female reproductive organ (archegonium) and argue that the ovule gene network originates from the archegonium, and may have first arisen by expressing post-fertilization archegonium gene networks within the developing sporophyte. They experimentally validated the role of sugar signaling by carbohydrate supplementation.
I congratulate the authors on providing the community with a plethora of new data, including methodology for reproductive research in Ceratopteris! The study is well conducted and the conclusions justified. I have but two concerns that I would like to ask the authors to address.

Both in the abstract and in the discussion the authors write about mis-expression of archegonium genes. The term mis-expression strikes me as odd, in an evolutionary sense, co-option of a gametophytic network to a sporophytic structure occurred and I would rather put it that way than calling it mis-expression.
Rather than being counter-intuitive, as the authors write, I find it straight-forward that gametophytic GRNs are converted to sporophytic structures during evolution. There are several examples described in the literature, in particular in P. patens. The authors describe one example in the discussion, but there are many more, relating e.g. to the function of vasculature control or the homeodomain KNOX/BELL control of generations, or the resemblance of parts of seed and spore germination.

Along similar lines, using a fern and hence the sister group to seed plants for this study makes sense. However, there is a plethora of data available for bryophytes, sister to vascular plants and spore-bearing as well. Of interest are in particular the moss P. patens, which is hermaphroditic, and the liverwort M. polymorpha, in which the sexes are separated into disctinct thalli. For example, a lot of studies has been carried out on the role of MADS genes, in particular in P. patens. I feel it would be proper to reference this literature and to put the results of the present work into context.

Version 1:

Reviewer comments:

Reviewer #1

(Remarks to the Author)
The authors have nicely addressed all my previous comments.

Reviewer #2

(Remarks to the Author)
Review

I found the authors have more or less adequately responded to my comments. Overall, I still find the manuscript difficult to read in certain places. The discussion is overly results-heavy. I also think the authors ought to explain what they mean by the following statement “merging of archegonium gene networks with those of a sporophytic primordia.” My interpretation of this is that our canonical understanding of the evolution of the ovule is supported by their data (e.g., a fern-like ancestral gametophyte with an archegonia wrapped up in a sporophytic-leaf/stem like structure).

Specific comments on the response to the previous review:

I don’t understand what this would mean from an evolutionary perspective: “ovule development more strongly resembles a shoot at the genetic level than a modified sporangium and undermining the assumptions of homology made during morphological comparisons between evolving structures.” This also ties in with the author’s response “We respectively disagree with Reviewer 2’s assertion that the ovule is simply a homologous structure with fern sporangia with a gametophyte retained”

While I understand the authors point and perspective about providing a molecular developmental analysis, it seems quite far-fetched to propose that the ovule—and thus the embryo—is not homologous across land plants. That is not to say that it isn’t true, evolution does not follow our conceptions of “reasonable” or “unreasonable,” but we would then need to ask much deeper and broader questions about the evolution of sex in plants (e.g., how does one evolve a novel egg producing structure, how does meiosis or fertilization arise independently in different cell lines), and it also raises questions about

interpretations of the nucellus (previously understood as the sporangium).

This statement that the authors make in the response needs further clarification and articulation in the manuscript. How do their data support this claim? “we believe we demonstrate a transfer of gene network from the gametophyte to the sporophyte phase, and that these are responsible for developmental changes in the sporophyte tissues that give rise to the ovule body plan.”

Line edits

Abstract
Line 38: before “found” add “we”

Line 46: I think one more sentence would benefit the abstract here. Particularly, a focus on what this means for the origin and evolution of the ovule. My interpretation is that these data support the homologous hypothesis that the ovule is derived from a retention of a single megasporangium on a megasporophyll (e.g., gene network similarities of both are found). So, just rounding out what the authors think will be helpful.

Introduction

Line 61: the parenthetical “(‘ovules’ before fertilization)” may confuse some readers. I understand that the authors mean that before fertilization seeds are called ovules, but some may interpret this as them meaning seeds are ovules before fertilization.

Line 63: The nucellus is also a nutritive tissue in some lineages

Line 72–75: the independent origin of fern leaves does not preclude the ancestral telomic axis bearing a sporangium as being the ancestor of the ovule.

Line 86: be careful not to perpetuate the trope of the hermaphroditic fern gametophyte. See Haufler, Christopher H., et al. "Sex and the single gametophyte: Revising the homosporous vascular plant life cycle in light of contemporary research." BioScience 66.11 (2016): 928-937.

Discussion

Line 529: I suggest using a more phylogenetically-minded term than “basal”

Line 549–550: typo “candidates genes”

Reviewer #3

(Remarks to the Author)
I thank the authors for having aptly dealt with my comments. I have no further remarks.

Version 2:

Reviewer comments:

Reviewer #2

(Remarks to the Author)
The authors have addressed most of my concerns. I think where we currently lie is in a disagreement of how to frame the work and whether comparisons between extant homosporous fern reproduction and seed plants are meaningful to make assessments about the transitions hat occurred in the common ancestor of seed plants. That is fine. I believe that the questions the authors ask are inherently interesting and I do appreciate the attempt of opening the "black box" of development to try to find a mechanistic molecular explanation for the origin of the ovule. I hope this work can be a foundation and springboard for future explorations into this question.

A few suggestions to help the authors make the manuscript more readable.

Line 43–46: "Our findings support a novel mechanistic model to explain the origin of the ovule from a sporangium-bearing axis, with the developmental modifications underpinning the ovule body plan arising from gene networks in the seed-like post-fertilization archegonium that became expressed during development of that sporangium-bearing axis." this statement is quite confusing. I understand what the authors are articulating, but it took me a few reads. I suggest rewording as follows: "Our findings support a novel mechanistic model to explain the origin of the ovule from a sporangium-bearing axis, whereby

gene networks in the post-fertilization archegonium of seed-free ancestors were heterotopically expressed during the development of the seed-plant sporangium-bearing axis."

Line 81-85: I may suggest flipping heterospory and endospory as it is likely that heterospory evovles prior to endospory (e.g., we see many heterosporous ferns that are not endosporous (Marsilieaceae), but we do not see any endosporous plants that are homosporous.

Line 503–505: it is not necessarily that the presence of similar gene expression is a smoking gun for heterotopy and leveraging ancestral GRN. I would suggest rewording.

Open Access This Peer Review File is licensed under a Creative Commons Attribution 4.0 International License, which permits use, sharing, adaptation, distribution and reproduction in any medium or format, as long as you give appropriate credit to the original author(s) and the source, provide a link to the Creative Commons license, and indicate if changes were made.
In cases where reviewers are anonymous, credit should be given to 'Anonymous Referee' and the source.
The images or other third party material in this Peer Review File are included in the article's Creative Commons license, unless indicated otherwise in a credit line to the material. If material is not included in the article's Creative Commons license and your intended use is not permitted by statutory regulation or exceeds the permitted use, you will need to obtain permission directly from the copyright holder.
To view a copy of this license, visit https://creativecommons.org/licenses/by/4.0/

Plackett & Catoni, Response to Reviewer Comments

Reviewer #1 (Remarks to the Author):

This work by Plackett and Catoni aimed to trace the origin of seed by generating and examining a comprehensive gene expression atlas for the fern Ceratopteris richardii. This is a monumental work, combining detailed characterizations of developmental progression and comparisons of gene expression across tissues and species. Importantly, a few follow-up experiments were conducted to test the hypotheses resulted from the RNA-seq data. The results are interesting, and would definitely lead to further research to test the various hypotheses raised in this study.

My overall take is that the authors are asking very important questions, using well-designed experiments and thorough data analyses.
That being said, the manuscript is complex and, to me, difficult to digest at places. The writing can be tightened up more. In addition, without further gene functional studies, one cannot conclusively link the genetic networks between ferns and Arabidopsis. For the most part the authors are cautious with the wording, but not consistently.

- We thank Reviewer 1 for their constructive criticism of the language used. Because of other changes to the manuscript we have substantially redrafted the Results and Discussion sections, and have attempted to revise our wording to make our meaning as clear as possible. Without specific examples to correct we have gone through the manuscript and attempted to ensure that we have been appropriately cautious with our wording to avoid over-claiming, for example referring to ‘annotated as orthologs’, ‘putative orthologs’ and ‘putative gene networks’.

- We thank Reviewer 1 for their point about gene functional studies to validate this work. While this would be ideal, the lifecycle of *Ceratopteris* (approx. 6-8 months spore to spore) means that this would take additional years of research, and as pointed out this manuscript is already monumental. As such, we have instead focussed on functional validation of predictions from our RNA-seq dataset that can be tested through other means, eg. chemical treatment. We would like to point out that in each case (**GA/PBZ treatment, Fig. 2; sucrose treatment, Fig. 6; auxin treatment, Fig. 6; JA treatment, Supplementary Fig 17**) our prediction was successfully validated by these methods, giving us high confidence in the quality of the RNA-seq dataset that we have generated.

A few detailed comments/suggestions below:
Line 30-33. These two first sentences in the abstract have very similar structure “… how… remained…” and should probably be revised.

- We have revised the beginning of the abstract to make sentences 1 and 2 address different points, as suggested (**lines 30-34**)

Line 54. I’m not a big fan of the use of “monilophytes”. I think it’s an unnecessary jargon; just refer them as “ferns”.

- We agree with Reviewer 1’s intention to reduce jargon in the field, but feel that at this point in the manuscript ‘monilophytes’ is phylogenetically the most precise term, because it refers collectively to the horsetails and ferns (which are generally recognised as separate

groups), and together these form the lineage that is the closest outgroup to the seed plants. To address this concern we have edited the manuscript to say 'monilophytes (horsetails and ferns, hereafter 'ferns') (**lines 54-55**) and changed all subsequent uses of 'monilophytes' to 'ferns' accordingly.

Line 82. "retained within early seeds and gymnosperms". Something is missing here.

- Thank you to Reviewer 1 for spotting this. We have edited the sentence to improve its clarity (**line 110-113**).

Line 167-169, 171-172. The authors expected the key genes to be differentially expressed between apex and primordia, but my understanding is that their Apx samples actually contained early primordia as well. I am not sure if such comparison is reasonable.

- We agree that the Apx samples contain primordia as well as the shoot apex, which reduces the sensitivity of our comparison, but we feel that this comparison remains reasonable because we are comparing samples with shoot apex + primordia to samples with primordia. Therefore any genes that show differentially increased expression in Apx samples, which we do detect, we think is reasonable to assign to the presence of the shoot apex within the sample. We have edited out Results section to explain this logic more clearly (**lines 186-188**)
- We agree that we are probably not finding all possible candidates, and have edited our discussion to acknowledge this limitation (**lines 534-534**).

Line 176. GA 20-oxidase is part of a larger gene family (cytochrome P450). It would be necessary to present a gene phylogeny to demonstrate this candidate is in fact an ortholog of GA 20ox.

- We have now included a gene tree of the orthogroup within which this putative CrGA20ox gene was identified (**Supplementary Fig. 8**) and refer to it in the Results (**lines 201-203**). This shows the candidate *CrGA20ox* gene forming a fern-specific outgroup to the majority of seed plant orthologs (including known *AtGA20ox* genes), with lycophytes and bryophytes sequences forming sequential outgroups to this. We feel that this reflects the evolutionary history of GA biosynthesis and signalling, which originated in the lycophytes.

Line 182. The phenotype from GA-treated plants is very interesting and exciting. I wonder if the authors could further interrogate the role of GA by using GA biosynthesis inhibitors. There are many inhibitors that work on different part of the pathway.

- We thank Reviewer 1 for this suggestion and agreed that the reciprocal experiment with GA biosynthesis inhibitors is an important further test of our conclusion. We have now performed this experiment using paclobutrazol (PBZ) and found that inhibiting GA biosynthesis delays the shoot sporing transition (along with other phenotypes) both chronologically and developmentally. We have incorporated these new results into the manuscript **(Fig. 2h-l, lines 212-218)**.

Line 233-237. This sentence is too long and apparently missing some words. I cannot quite follow it.

- Thank you to the Reviewer for highlighting this. We have divided the points in this sentence across two to increase clarity. **(lines 286-290)**.

Line 291. "Associated genes were mostly related to auxin". But in Fig. 6, I could see many JA related GO terms. I wonder if the authors have looked into JA and why JA wasn't highlighted in the text.

- We thank Reviewer 1 for highlighting this point. We have conducted additional experiments to test for a role for JA in gametophyte development, and found that it acts to negatively regulate the activity of the notch meristem based on thallus size and the number of archegonia produced at 14 DAS. This is consistent with the pattern of GO term enrichment seen, where JA related terms are mostly enriched before and just after fertilization. We have included this new information in the manuscript (**Supplementary Fig. 17; lines 380-388**).

- In addition, we have rearranged data presented between the main and supplementary figures to better highlight how our GO term analysis supports a role for auxin (**Fig. 6f**), and clarified this in the manuscript text (lines **388-390**).

Line 295-298. So auxin treatment changed phenotypes of archegonia, but what does it mean? Does this finding support anything?

- We thank Reviewer 1 for highlighting this. We have expanded on this point by including additional images in a more logical order (**Fig. 6g-j**), showing the effect of auxin treatment both on embryo-bearing archegonia and embryoless archegonia. We believe that this shows two things:

  1. Growth of the archegonium envelope (calyptra) is normally co-ordinated with embryo growth, but becomes deregulated under NAA treatment. This resembles the coordinating role that auxin has during seed development between the embryo, endosperm and seed coat.

  2. Under auxin treatment we observed what appears to be calyptra development and growth in archegonia where no embryo is present, and so is presumably unfertilized. This strongly parallels the well-known ability of auxin to trigger parthenocarpic growth of unfertilized seeds because auxin is an important signal of successful fertilization. Collectively these points suggest to us that auxin regulation of post fertilization archegonium development and seed development are similar, and may be conserved.

- We have now revised our Results section to provide a more detailed explanation about these points and how they relate to known functions in Arabidopsis seed development (**lines 390-401**).

Line 390. I think "unambiguously" is too strong here.

- Thank you to Reviewer 1 for pointing this out. We have removed this term from the manuscript to avoid over-claiming (**line 521**).

Line 422-425. I might have missed something but I couldn't see the link between "homosporous meiotic gene networks" and "seed plant microsporogenesis".

- We thank Reviewer 1 for highlighting this. We have substantially redrafted the Discussion, and as part of this we have introduced a section discussing the evolution of sporogenesis and microsporogenesis in more detail and with reference to what is known in bryophytes. Essentially we find that the genes that regulate pollen development (microsporogenesis) in flowering plants are also identified as regulating sporogenesis in the homosporous fern. This suggests that the regulatory gene networks between the two are conserved, which is consistent with the developmental similarity observed between them. We have included this explanation in the manuscript text (**lines 548-558**)

Line 578-580. It is important to state exactly how novogene carried out read-mapping, assembly, and transcript quantification and annotation.

- We thank Reviewer 1 for catching this omission. We have now included more detail on the methods used (**lines 794-802**).

Supplementary Fig. 5. The image resolution for PCA plots is too low.

- We thank Reviewer 1for pointing this out. We found that this was an artefact of the plots being resized and flattened. We have simplified the labelling of the PCA plots to improve clarity and increased their size to improve legibility (**Supplementary Fig. 5**). To achieve this the correlation plots have been moved to separate files. (**Supplementary Figs. 6 and 13**)

Reviewer #2 (Remarks to the Author):

The manuscript entitled "The post-fertilization archegonium gene networks of seedless plants contributed to the origin of the seed" aims to explore the molecular origin of the ovule in seed plants by analyzing into the gene networks of fern sporogenesis and archegonia development.

I want to start this review by saying that I greatly enjoy this topic and understanding the evolution of reproductive structures in plants is a topic with broad interest. I think this work is worthwhile and the questions are important to explore. I find the work of the authors to be extremely robust, rigorous, and heroic in scale—there is a lot of data here.

However, I have many issues with the argument, hypotheses proposed, and evolutionary/developmental explanations. While I do not think the manuscript or the hypotheses in their current state should move forward, I hope that the authors can view my review as constructive and not destructive, as that is my intention.

- We thank Reviewer 2 very much for their very thoughtful and expert review. We believe that many of the criticisms given below are due to our not having explained our hypotheses sufficiently clearly, or explained the implications of our findings well enough. We have attempted to remedy this, as outlined in our responses below.

First, the authors present a molecular-level hypothesis for the origin of the seed, proposing that "mis-expression" of archegonium gene networks played a key role in the origin of the ovule. This hypothesis appears to stem from correlations of gene networks in homologous structures (e.g., developing egg-bearing structures in ferns and ovules, which are developing

egg-bearing structures in seed plants). As the authors know and explain, in seed plants sporangia are modified into the nucellus (with some modification and addition of other maternal tissue), within which a single surviving meiotic product gives rise to the female gametophyte and subsequently the egg-bearing structure. Thus, this paper, while a heroic amount of work, seems to simply demonstrate that there are similar gene networks in homologous structures (e.g., some parts of the ovule and some parts of the egg-bearing portion of the developing fern gametophyte). If the canonical evolutionary process is true, then the evolution of the ovule is brought about by the retention of the gametophyte within the sporangium and this would be borne on the mother sporophyte. In this case, it is axiomatic that egg-producing gene expression in seedless plants would be observed in egg-producing structures of seed plants. Put another way, the gene networks in fern archegonia (and during embryogenesis) should inherently mirror those of seed plants because ovules are essentially archegonia (or egg-bearing structures) enveloped in maternal tissue. These genetic correlations highlight that egg-bearing structures across plants are homologous and exhibit similar gene networks without providing deeper evolutionary insights. Moreover, the specifics of this hypothesis are also tenuous, and they are supported by similarities in sugar transport and auxin signaling genes. These processes are ubiquitous in all plant organs and their similar expression between archegonia and ovules is unconvincing.

- We sincerely thank Reviewer 2 for making these points and the spirit in which they were made.

- While we agree that from first principles it is likely that the ovule arose from by modifying a sporangium ss suggested by comparative morphology, we do not believe that this has been proven beyond doubt, with the developmental-genetic evidence suggesting that ovule development more strongly resembles a shoot at the genetic level than a modified sporangium and undermining the assumptions of homology made during morphological comparisons between evolving structures (summarised in Mathews & Kramer 2012 https://nph.onlinelibrary.wiley.com/doi/10.1111/j.1469-8137.2012.04091.x). We have expanded upon this point in the introduction to make the context for this investigation more clear (**lines 98-104**).

- Our primary purpose behind this research is that we do not understand the process by which such a transformation came about- *how* was development changed, not merely what changes took place. In order to understand how development was changed we need to understand how the underlying gene networks changed. We have redrafted parts of the introduction to make that focus clearer (**lines 56-59; 93-96; 104-106**).

- Consequently, in this work we believe that we have tested the hypothesis that ovule gene networks arose by repurposing gene networks involved in ancestral development, and that the original function/location of these ancestral gene networks informs us about the developmental origins of the ovule. We have revised our Introduction to clarify this (**lines 117-120**).

- We respectively disagree with Reviewer 2's assertion that the ovule is simply a homologous structure with fern sporangia with a gametophyte retained:
  1. We do not believe this is in fact fully proven (see above).
  2. This view does not take into account ovule-specific modifications such as the multi-layer integument, the nucellus acting as a support/nutritive structure, or apical modifications for pollen reception, nor does it take into account the modifications that have arisen in the

gametophyte (reduction, the invention of 'femaleness'). There a great deal of ovule-specific development that we do not yet understand, nor do we understand how it evolved. We have included this point in our revised introduction to clarify our reasoning for taking this approach (**lines 90-93**).

- We agree that many of our results are correlative, but we consistently compare against a vegetative dataset as a negative control, and we believe that through that they are rigorous and meaningful. We have also taken pains to validate what predictions we can experimentally arising from our RNA-seq data, and in each case those predictions have been proven correct (**GA/PBZ treatment, Fig. 2; sucrose treatment, Fig. 6; auxin treatment, Fig. 6; JA treatment, Supplementary Fig 17**).

- Re. Reviewer 2's conclusion that our results stem from finding homologous gene networks, we would like to highlight that our results do not in fact simply show homology gametophyte-to-gametophyte within the ovule. We apologise that this was not presented sufficiently clear in our first submission. Our enrichment analysis specifically shows enrichment of orthologs of fern gametophyte genes in pre-meiotic ovules (nucellus and MMC). As such, the explanation that we see overlap because the gametophyte is still present is not valid- we believe we demonstrate a transfer of gene network from the gametophyte to the sporophyte phase, and that these are responsible for developmental changes in the sporophyte tissues that give rise to the ovule body plan. We have undertaken additional analyses to demonstrate this point more clearly (**Fig. 9, lines 474-494**), identifying ovule-specific genes within those enriched just in nucellus tissue (eg. BEL1). We have also redrafted the Discussion to better clarify the evolutionary implications of this finding (**line 578-590**).

- We agree that auxin and sugar have many roles in angiosperm development, but both trigger seed-specific reactions not observed in other development contexts. We find specific parallels to both of these in archegonium development:

  1. Auxin has seed specific functions triggering fertilization dependent development and coordinated growth between embryo, seed and seed coat, which are distinct from auxin's role in organogenesis elsewhere in plant development. We see similar processes disrupted when we apply auxin to the archegonium, which was applied specifically at the point of fertilization to prevent confusion of our results by other pleiotropic effects. These results strongly suggest that the same seed-specific processes are present in both, and are not simply a general auxin response, and this is supported by the specific enrichment of auxin GO terms in the archegonium tissues. We have expanded our results figure (**Fig. 6e-j**) and manuscript text to better clarify our explanation of this result (**lines 356-375**).

  2. Sugar signalling is a common feature in plant processes but the seed response is highly specific, ie grain filling vs causing abortion. This is triggered by trehalose-6-phosphate as a signalling molecule. Here we show that orthologs of trehalose-6-phosphate genes are expressed specifically in reproductive tissues at a very specific stage of development, during fertilization, and that applying sucrose has a direct effect on fertilization success. The generic effect of sucrose treatment on fern gametophytes is to trigger apogamy (Cordle et al. 2007, https://www.journals.uchicago.edu/doi/full/10.1086/511049) and we have now discounted that possibility. As such, the response we see strongly parallels the known sugar signalling mechanism and response in seed development. We have edited our results figure (**Fig. 6a-d**) and manuscript text to better clarify our explanation of this result (**lines 388-401**).

Zooming out for a moment, the authors' assertion that ovules arose from fertilized archegonium gene networks also lacks clarity overall clarity. Perhaps I do not understand the processes here but what does this hypothesis even mean from a developmental or evolutionary perspective? How exactly does the heterotopy of archegonial developmental genes (or embryogenesis gene networks) lead to the evolution of the ovule? This needs to be fleshed out in much more detail.

- We thank Reviewer 2 for asking for clarification on this. Our point is to understand how such a novel organ as the ovule (which, as we state above, we believe is developmentally more complex than a sporangium containing a gametophyte) could have arisen from existing gene networks. We have edited our introduction to make this point more clearly (**lines 94-96; 117-120**).

- From our updated results, we believe our evidence suggests that this was achieved by the merger of two pre-existing gene networks- one in sporophyte organ primordia and one in the post-fertilization archegonium. Combining these two gene networks in novel combination could explain the novel development that we observe in the ovule. We have revised our Discussion to explain this point and what support our data provides for it (**lines 620-635**).

The lack of clarity around the hypothesis is also furthered by other connected but tangential hypotheses laid out in the discussion. For instance, line 427 the authors make a statement about the origin of heterospory, however the main claim elsewhere is about ovule development not the heterospory. In this same vein, in line 413 the authors indicate that floral and sporophyll reproductive networks may have arisen from ancestral genetic networks. Again, anthers and fern sporophylls would be homologous structures (structures bearing sporangia). In this case it is not only separate from their primary claim, but also it seems like the authors are observing that microsporangia in seed plants and sporangia in homosporous ferns express similar gene networks, which is interesting, but not necessarily surprising. Rather than providing new frameworks or hypotheses this latter point supports the hypothesis that anthers may be homologous to spore-bearing structures in ferns.

- We thank Reviewer 2 for this point about tangenital hypotheses. To improve clarity of the manuscript we have removed the more speculative claims regarding heterospory and floral gene networks from the Discussion section.

- Re. the point about stamen evolution, we believe that it is appropriate to include and present this information because it is essential for understanding ovule evolutionary origins: we show that there is not significant enrichment between the sporophyll and the ovule, which as the fern sporangium-bearing organ was the primary candidate for the ancestor of the ovule following the prevailing hypotheses about organ homology raised yourself in your comments above. We feel that this negative result is thus as important to understanding the evolution of the ovule as the positive finding that there is homology with the archegonium. We have revised our Discussion to clarify this point (**lines 607-617**).

- In order to avoid this additional information being distracting, we have re-ordered and reformatted the Discussion into three sections (shoot reproductive transition, **lines 525-545**; stamen evolution, **lines 547-574** ; and ovule evolution, lines **576-635**).

Another important point is that it would be much more poignant to compare ovule development with megasporophyll development in a heterosporous fern or lycophytes, not homosporous ferns. The fact that the authors only found that archegonia-related genes are related to ovule genes and not sporophyll gene is not surprising and I wonder if a comparison between megasporophyll development would be more likely to yield interesting results.

- We must strongly disagree with this suggestion. Phylogenetic analysis shows that heterospory in ferns has an independent origin from that in seed plants, much as it is independent in the lycophytes. Because they are independent origins we cannot make an assumption that they will have arisen in the same way or using the same gene networks, and if they have then that is convergent evolution. Comparing heterospory in seed plants to heterospory in ferns without understanding the ancestral state (homospory) risks identifying false homologies. To understand how gene networks evolved we have to compare from the ancestral to the derived state, or as near as we can achieve. We have used *Ceratopteris* for this analysis precisely because it is a homosporous fern and as such represents the closest proxy we can have to the ancestral homosporous seed plant ancestor.

- We again argue that not finding homology between the ovule and sporophyll is in fact surprising, because many ovule tissues are of sporophytic origin and it undergoes sporogenesis, which is what happens in the fern sporophyll but does not in the fern archegonium, which develops as a haploid structure.

- Further to this, in our updated analysis we have now found independent enrichment of ovule genes with a fern sporophytic organ, but the fern frond and not genes specific to the sporophyll. This has allowed us to make a much more specific prediction as to the mechanism through which the ovule gene network was first assembled, i.e. the archegonium gene network became merged with the sporophytic gene network and became expressed in sporophytic tissues. We have redrafted our Discussion to explain these points and the evidence supporting them (**lines 607-635**).

Importantly, this work would be enhanced by invoking developmental processes such as heterotopy or heterochrony, if that is what the authors mean by "repurposing ancestral reproductive gene networks." However, I would argue that it is not "repurposing" as the genes are still being expressed in homologous structures (e.g., egg producing structures in both ferns and seed plants), so I don't really understand what the new hypothesis is.

- Our understanding of these terms is that heterotopy and heterochrony refer to the movement of organs in either position or the timing of their development in an organism. What we are describing here is the change in spatial and temporal expression of gene networks to affect development and thus change the nature of an organ. We believe that this is a slightly different concept and so are reluctant to use the terms in case it causes confusion.

- We believe we have found evidence for at least some of the gene networks from ancestral organs being repurposed in different tissues/generations because we see enrichment of archegonium genes (from the haploid gametophyte) in pre-meiotic (i.e. sporophytic) ovule tissues, and specifically so- if we filter out the female gametophyte-expressed genes we still see significant enrichment. We have attempted to clarify this point in the data presented in our revised **Fig 9**, and the accompanying manuscript text (**Lines 474-494**).

Finally, the authors should refine their comparisons between homologous structures (lines 409–413) and provide greater specificity regarding developmental processes invoked in lines 424–425. Overall, while the topic is intriguing, the hypotheses and the paper require significant clarification and refinement.

- Thank you to Reviewer 2 for this suggestion. Re the comparison between homologous structures, with additional analysis we have refined the specificity of where gene networks are conserved between angiosperm and fern reproductive structures:

  1. Stamen genes are conserved with late sporophyll development, coincident with sporogenesis. We also only see significant enrichment with the reproductive genes modifying frond development into the sporophyll. As such, this does not support direct homology between sporophylls and stamens but rather is consistent with the theory that fern and seed plant lateral organs are not homologous, and that the same modifying gene networks were inherited by independently-originating lateral organs. We have updated the discussion to make this point (**lines 561-574**).

  2. Ovule genes are conserved with the post-fertilization archegonium, and that conservation includes sporophytic genes in the pre-meiotic ovule as well as the Arabidopsis female gametophytes. With our updated analysis we also find significant enrichment specifically with the frond primordium. This suggests that, in terms of development, the ovule gene network represents an 'archegoniumized primordium'. We have updated out Results and Discussion sections to more clearly outline this argument (**lines 496-513; 620-635**).

- Re specificity of developmental processes, we have undertaken additional analysis to identify the gene orthologs that show enrichment to better understand the processes involved. These data have now been presented in **Supplementary Data 9** and **Supplementary Data 10** and their implications highlighted in the manuscript (**lines 484-492, 501-508, 592-602, 624-625**).

  3. Around meiosis, we have revised this Discussion section to include much greater detail about the genes involved and their level of conservation (**lines 549-559**).

  4. In addition, our updated analysis has allowed us to be more specific about the developmental processes regulated by the two conserved ovule gene networks in the fern frond primordia and the post-fertilization archegonium, with the earliest stages of ovule specification represented in the frond primordium. We have updated our Results and Discussion section to include this (**lines 501-508, 624-625**).

I also will say that most of the figures and data generated and presented, while interesting, do not bear on the main claim of the paper. Methodologically, it may also be important to run a differential gene expression analysis using deseq2 or masigpro across developmental stages.

- We thank Reviewer 2 for this suggestion. We have conducted further differential expression analysis as suggested, in addition to our existing DE analysis to identify our reproductive candidate genes, by comparing gene expression across development of each organ and identifying genes differentially-regulated specifically in reproductive tissues. With these we have been able to additionally validate our reproductive candidate genes as being involved in reproduction-specific processes and refine some of our conclusions about gene expression patterns. We have incorporated these results into our Supplementary Material (**Supplementary Figures 7, 9, 10, 14, 16, 20, 21; Supplementary Data 3)** and have made reference to them throughout the Results section where appropriate.

- We agree that much of the data we present is in addition to the central claim of this paper but it nevertheless arises from the large-scale RNA-seq analysis that we needed to conduct to gather evidence for that main claim. We argue that all of the data presented is relevant to the conservation or otherwise of reproductive gene networks between ferns and seed plants and thus has value and bearing on the evolution of seed plant reproductive structures. The title of this paper simply picks out the most interesting finding from this analysis, which we believe is often common practice. We have reformatted our Discussion section to separate out these different themes to make that more apparent (shoot reproductive transition, **lines 525-545**; stamen evolution, **lines 547-574** ; and ovule evolution, lines **576-635**) and to avoid confusing the reader in thinking all of these have a direct bearing on the title of the paper.

Furthermore, specific line edits are necessary to streamline the writing. Please see below.

I want to end where I started and say that I do believe this work is exciting and important. However, I think the claims proposed by the authors are somewhat unclear from a developmental and evolutionary perspective and I further do not think the data the authors generated support their claims (in the way I perceive them).

Line edits

30-32: two back to back statements of unsolved questions could be streamlined.

- We thank Reviewer 2 for highlighting this, and we have redrafted to avoid it (**lines 30-34**).

57: It is important to caution against these claims that the seed and the flower are the major dominant trait promoting seed/flowering plant diversification. The Acrogymnosperms are quite species poor and many of the sister group angiosperms (e.g., ANA grade) are also quite species poor. Neither the seed nor the flower alone likely is responsible for the major diversity of flowering plants—rather it is a few hypoer diverse groups of flowering plants that contribute mostly to the impressive diversity of the group. This has to do with unique floral morphologies and pollinator interactions (O'Meara et al., 2016 Proc B.).

- We thank Reviewer 2 for this insight. We have removed this claim from the introduction and instead focus on the extent of developmental change involved in the evolution of the seed which remains unexplored (**lines 55-59**).

Line 57-59: The body plan of the ovule is no different than the hypothetical scenario of a fern maintaining a single endorsporically developed megagametophyte on the parent sporophyte plant. The ovule is of course an interesting evolutionary innovation, but it is inherited from previous structures with clear holomogies.

- We apologise but we do not understand how this comment relates to the lines listed. We have gone through the introduction and tried to make sure that we clearly describe the theories as to which organs the ovule is believed to be homologous with (**lines 98-100**).

- We also believe that our definition of 'body-plan' must not be clear- we are referring to the patterning of the ovule tissues and structure within the organ, which we would argue is distinct and more complex from a single sporangium on the parent plant- its development is highly shoot-like. We respectfully believe the Reviewer is mistaking our developmental definition for one of organ homologies. To resolve this we have clarified what we mean by the term (**lines 61-65**).

Line 85-86: redundant use of pollen and embryo sac

- We thank Reviewer 2 for pointing this out- we have edited 'pollen sac' to 'anther' to avoid redundancy (**line 83**). We have further avoided the use of 'embryo sac' and for clarity now refer to the female gametophyte ('FG') throughout.

Line 390: I am unsure about this claim.

- In response to reviewers' concerns we have modified this claim to be less definite and removed 'unambiguously'. (**line 522-523**)

Line 397: the authors indicate "but three Ceratopteris homologs were identified with expression patterns consistent with downstream targets of the sporing transition. This could indicate that the GA-MADS module of angiosperm shoots was co-opted from an ancestral mechanism in the vascular plant shoot reproductive transition." To confirm this the authors would want to build gene trees and observe the phylogenetic placement of the MADS-box genes expressed in Ceratopteris and those expressed in developing ovules. But moreover, what are the authors even implying from this statement? What about the MADS module in fern reproduction would have been co-opted for shoot reproductive transition? Clarity around the actual developmental shifts and what they mean are important to explain.

- We thank Reviewer 2 for these points. In our updated analysis the enrichment of and significance of MADS-box genes was substantially reduced, and as such we have removed this speculative point from the discussion.

Line 409-413: these are homologous structures (structures bearing sporangia). It seems like the authors are just observing that microsporangia in seed plants and sporangia in ferns express similar gene networks.

- We have modified this section of the Discussion to incorporate new data that allows a more specific inference about the homology of gene networks between sporophylls and stamens (**lines 561-574**). Stamen genes are specifically conserved only with late sporophyll development (ie. the onset of meiosis) and not development of the whole organ.

- We also must content that we believe this point about conservation of genes between sporogenesis and microsporogenesis is in fact of evolutionary interest, because one is homosporous and the other is a heterosporous state and we do not yet know the ancestral gene network- how similar are the gene networks regulating homospory and microsporogenesis? Knowing this will have implications for our understanding of the evolution of heterospory. We have updated the discussion to make the evolutionary significance of this information more clear (**lines 549-559**).

- We would also like to draw Reviewer 2's attention to more recent genetic studies that suggest that seed plant leaves and fern fronds are not homologous- as such, logically the sporophyll and stamen cannot be homologous organs. To resolve this we have rephrased out Discussion to refer to inheritance of gene networks from the sporangium-bearing organ in the last common ancestor (**lines 571-574**).

Line 416-417: this is somewhat confusing, perhaps it is just syntactic. From reading this it seems like the authors are saying that SGO and ZIP4 are produced in the developing sporophyte and also the gametophytic notch, but what do the authors mean by post-meiotic? Do you mean that the entire gametophyte is post meiotic (e.g., derived from a spore that is the product of meiosis?). If so, it just reads a bit odd given that the entire gametophyte of any plant (seedless or seeded) is post meiotic.

- In our updated analysis, using an Orthofinder dataset including a heterosporous fern as requested (amongst other additional genomes, please see below), these two genes were no longer annotated as orthologs of SGO and ZIP4, and so this section has been removed.

Line 424-425: what do the authors mean when they say "female-specific meiosis mechanisms could have diverged by co-opting pre-existing notch gene networks already utilising meiosis genes." What exactly is the developmental process here? I believe this is a case of invoking causation from genetic correlations. It also may be more important to look at heterosporous ferns or lycophytes and compare the development of megasporophylls with ovule development.

- We thank Reviewer 2 for this question- many of the genes identified play a role in meiosis but have additional roles in the somatic cell cycle (mitosis), The archegonium is a rapidly-growing and dividing tissue and so it is likely that the genes identified are in fact involved in mitosis, but have the capacity to undertake meiosis. As such, we speculate that this could be a route through which female meiosis evolved: gene networks from the archegonium may have become merged through these common genes with the sporogenesis programme to result in a modified form of meiosis. However, to avoid confusion and increase clarity we have removed this hypothesis from the manuscript.

Line 437: More than embryogenesis what are the "striking functional similarities between the post-fertilization fern archegonium and the seed" ?

- We thank Reviewer 2 for their question. We are referring to the post-fertilization growth of the archegonium calyptra to keep the embryo enclosed, analogous to the seed coat, and the development of a placenta to provide the embryo with nutrients, analgous to an endosperm. We have now included this information explicitly in the Introduction to clarify our meaning (**lines 107-110**).

Fig 1. How could the authors tell between fertile and sterile leaf primordia. Figure g/m look awfully similar.

- We thank the reviewer for this question. Sterile and fertile leaf primordia were harvested from plants of different ages, those at 60d not yet producing spores and those of 130d all producing sporophylls (as validated and quantified across multiple repeat populations by our characterisation of sporing, Fig 1a-c). As such, there was no danger of confusing the two primordia because they were harvested from different plants at different times. We have updated the Methods section to clarify this (**line 742-744**).

Methods:

Line 470: no capitalization after period "individual"

- Thank you, corrected.

Line 532: what are the different frond developmental stages?

- The frond developmental stages are those described in Figure 1 and in the accompanying results section (lines 146-149). We have updated the Methods section (**lines 738-741**) to make this description standalone.

Line 582: Glycine not italicized

- Corrected, with thanks.

Line 584: seems like this analysis would benefit from having heterosporous fern genomes in it.

- In response to Reviewer 2's concern we have rerun our analysis with the following additional genomes to improve sampling in under-represented lineages to improve the robustness of our analysis:
  - Ferns: *Marsilea vestita* (heterosporous), *Adiantium capillus-veneris* (homosporous), *Alsophila spinulosa* (homosporous).
  - Lycophytes: *Lycopodium clavatum* (homosporous).
  - Gymnosperms: *Gnetum montanum* (Gnetales), *Cycas panzhihuaensis* (Cycad), *Thuja plicata* (Cupressaceae).

- Our analysis thus now includes homosporous and heterosporous species in both the fern and lycophyte lineages.

- We have updated all of our downstream enrichment analysis based on the new orthology (GO term enrichment, Arabidopsis ortholog enrichment). We wish to highlight that the primary result of our analysis remains unchanged, ie. that ovule gene networks are conserved specifically with post-fertilization archegonium development in the fern.

- As a result of this improved analysis we have also detected a second hotspot of conservation with the genes regulating general fern frond development, not those associated with sporophyll development. This gene network is overlapping but distinct

from that found in the archegonium, and has allowed us to propose a more specific mechanism as to how the ovule gene network was first formed in a sporophytic organ.

- We have redrafted the Results and Discussion to include these new results (**lines 457-516; 607-635**).

Reviewer #3 (Remarks to the Author):

The evolution of seeds from previously spore-bearing plants is enigmatic. In this paper the authors analysed the network of seed genes in the non-seed plant Ceratopteris based on novel RNA-seq data of reproductive stages. The idea behind is was to determine how the seed-development network evolved. They find that seed genes are enriched specifically in the female reproductive organ (archegonium) and argue that the ovule gene network originates from the archegonium, and may have first arisen by expressing post-fertilization archegonium gene networks within the developing sporophyte. They experimentally validated the role of sugar signaling by carbohydrate supplementation.
I congratulate the authors on providing the community with a plethora of new data, including methodology for reproductive research in Ceratopteris! The study is well conducted and the conclusions justified. I have but two concerns that I would like to ask the authors to address.

- We would like to thank Reviewer 3 very much for their kind comments.

Both in the abstract and in the discussion the authors write about mis-expression of archegonium genes. The term mis-expression strikes me as odd, in an evolutionary sense, co-option of a gametophytic network to a sporophytic structure occurred and I would rather put it that way than calling it mis-expression.

- We thank Reviewer 3 for making this point. We have removed the term mis-expressed from these sections and instead refer to the ‘merging’ of gene networks (**lines 45, 621, 627-629**). We hope that this term will be acceptable.

Rather than being counter-intuitive, as the authors write, I find it straight-forward that gametophytic GRNs are converted to sporophytic structures during evolution. There are several examples described in the literature, in particular in P. patens. The authors describe one example in the discussion, but there are many more, relating e.g. to the function of vasculature control or the homeodomain KNOX/BELL control of generations, or the resemblance of parts of seed and spore germination.

- We thank the Reviewer for highlighting these. We have removed reference to this evolutionary change being ‘counter-intuitive’.

- We have included the additional example of KNOX/BEL control of generations in *Physcomitrium* as requested, but for reasons of space we do not feel able to provide an exhaustive list of examples. We apologise for this but instead highlight the existence of other examples (**lines 630-632**).

Along similar lines, using a fern and hence the sister group to seed plants for this study makes sense. However, there is a plethora of data available for bryophytes, sister to vascular plants

and spore-bearing as well. Of interest are in particular the moss P. patens, which is hermaphroditic, and the liverwort M. polymorpha, in which the sexes are separated into disctinct thalli. For example, a lot of studies has been carried out on the role of MADS genes, in particular in P. patens. I feel it would be proper to reference this literature and to put the results of the present work into context.

- We thank Reviewer 3 for their suggestion. In response we have included reference to the bryophyte and lycophyte literature in the following Discussion sections in what we hope is an appropriate way:

  1. The evolution of GA signalling (**lines 528-532**)
  2. The evolution of sporogenesis gene networks (**lines 552-555**).

- Unfortunately, for reasons of space we have had to remove our discussion on MADS-box genes in favour of other less speculative results.

Plackett & Catoni, Response to Reviewer Comments

- In the interest of transparency we would like to highlight to all Reviewers that during our latest revision we identified an error in one of the supplementary analyses included in the previous revision, namely our identification of genes showing differential regulation (DR) between developmental stages within frond development (**Supplementary Fig. 7, Supplementary Dataset 3**). We apologise to the reviewers for not spotting this earlier. We have corrected this analysis and those downstream analyses/datasets dependent on it (**Supplementary Fig. 10**, **Supplementary Dataset 4 + 5**; **Supplementary Fig. 20**, **Supplementary Dataset 8 + 9 + 10**), and have updated the mentions to these analyses' results within the manuscript Results to accurately reflect these (**lines 183, 258-260**).

- We wish to highlight that the updated results of these revised analyses **do not significantly alter the main claims or conclusions of our manuscript**, with overall significance not changing in the majority of the analyses. The principal difference is in results for enrichment of Arabidopsis reproductive gene networks in genes DR during sporophyll and frond development. Previously this analysis showed significant enrichment between vegetative fronds and the ovule. In our corrected analysis there is no significant enrichment in ovule genes in either the sporophyll or frond DR datasets, **but our parallel analysis showing significant enrichment between the sporophyll primordium and notch-conserved ovule genes remains unaffected**.
  -> In consequence, reference to conservation between the ovule and vegetative frond has been removed from the Results (**Figure 8, Figure 9**) and Discussion, where we had previously suggested based on this result that the ovule may have arisen by modifying sporophytic vegetative frond gene networks. With our revised analysis we believe that this is no longer supported, with an origin by modifying sporophyll gene networks now more likely, supporting existing morphological and fossil-based hypotheses.
  -> We have replaced the data in **Figure 8e** with our prior analysis of notch-conserved ovule genes in the sporophyll (moved from **Supplementary Figure 21**). We have increased the detail provided about this analysis in the Results to explain this data more fully (**lines 456-461, 485-488**).

- The updated results of our DR enrichment analyses instead show increased support for conservation between the sporophyll and stamen gene networks (**Supplementary Fig. 20, Supplementary Dataset 9**) which was not detected previously. This corrected result is consistent with our analysis of sporophyll reproductive candidates (**Figure 7**). We have redrafted this section of the Results to highlight this point (**lines 426-429**).

Reviewer #1 (Remarks to the Author):
The authors have nicely addressed all my previous comments.

- Thank you- we are glad that our edits were able to address Reviewer 1's concerns.

Reviewer #2 (Remarks to the Author):

I found the authors have more or less adequately responded to my comments. Overall, I still find the manuscript difficult to read in certain places. The discussion is overly results-heavy.

- We thank Reviewer 2 for this constructive criticism. In response to this, and where not essential to explain the point being made, we have redrafted a majority of the Discussion (**lines 533-617**) to minimise repeating descriptions of our findings and details such as gene names to focus more clearly on their underlying evolutionary significance.

I also think the authors ought to explain what they mean by the following statement “merging of archegonium gene networks with those of a sporophytic primordia.” My interpretation of this is that our canonical understanding of the evolution of the ovule is supported by their data (e.g., a fern-like ancestral gametophyte with an archegonia wrapped up in a sporophytic-leaf/stem like structure).

- We thank Reviewer 2 for highlighting the need to clarify this. We have now expanded upon this point in far more detail the Discussion to better explain our meaning (**lines 592-603**). In brief, we believe our results support a scenario in which genes normally expressed in the post-fertilization archegonium started to be expressed during the development of the ancestral sporophytic organ that would become the sporophyte component of the ovule. We think our data supports this expression occurring in the organ’s development prior to initiation of the sporangia, thus modifying development of that sporophytic organ to bring about the ancestral ovule body-plan.

- Reading through Reviewer 2’s comments here and below, we think a number of points stem from an unintentional misalignment of perspectives/assumptions. Our understanding is that Reviewer 2’s criticisms are focussed on the idea of evolutionary homologies. We would like to clarify that we are not trying to challenge the canonical view that the ovule evolved from a sporophytic stem-like structure, and we apologise to Reviewer 2 if we have mistakenly given that impression in the manuscript. In response to this misunderstanding we have edited the manuscript to make this point clearer (**lines 42-43, 463-465, 567-568, 582-583, 592-603**).
- We are instead attempting to explain how that ancestral sporophytic structure’s development changed to become the ovule, which is what we believe current evolutionary theories stop short of explaining- the ‘devo’ in evo-devo, if you will. To this end, we believe we have identified a major source of those developmental modifications as gene networks that were ancestrally part of archegonium post-fertilization development but have now become part of the pre-fertilization ovule gene network. This is the advance in knowledge and understanding that we believe that this manuscript delivers. We have edited the manuscript to to clarify our intended focus within this topic (**lines 42-46, 508-510**).

Specific comments on the response to the previous review:

I don’t understand what this would mean from an evolutionary perspective: “ovule development more strongly resembles a shoot at the genetic level than a modified sporangium and undermining the assumptions of homology made during morphological comparisons between evolving structures.”

- We thank Reviewer 2 for raising this query. Our intended point here was that, as currently articulated in the primary literature, the published developmental-genetic evidence does not unambiguously support the identity of extant ovules as sporangia as would be predicted

based on comparative morphology with seedless plants and the fossil record. To elaborate this point:

1. The gene network that regulates ovule initiation is more similar to an angiosperm vegetative lateral organ primordium than to a seedless plant sporangium. In part this could simply be due to a lack of data about the genes involved in ancestral sporangium development (which this manuscript now provides), but this perceived contradiction opens up an alternative hypothesis for the origin of ovules, namely that ovules evolved *de novo* from a shoot primordium that was modified by other gene networks, rather than evolving from pre-existing sporangium-bearing structure. This is reinforced by our understanding of development in extant plants, which shows how plastic (ie. variable) plant form can be with relatively few changes in the underlying genes.

2. In evolutionary terms, this variability therefore calls into doubt whether previous morphologically-based studies compared like-with-like when assigning the ancestry of ovules to sporangium-bearing axes of seedless plants. As an example of this, we would like to highlight the long-running (and continuing) controversy in the literature over the relationship of gymnosperm lineages to the angiosperms due to the differences between the conclusions of cladistic reconstructions of the seed plants based on morphological characters and the conclusions of those based on phylogenetics. To better illustrate this point in the manuscript introduction, we have included reference to this past controversy as (**lines 107 - 109**).

3. We are at pains to stress that we are not saying here that we believe the morphologically-based analyses are wrong, but that the two contrasting strands of evidence have created ambiguity which needs to be resolved (as already stated in the manuscript, **lines 103-107**). We believe that the information about gene networks regulating sporophyll development presented in this manuscript goes a long way towards helping resolve this ambiguity, showing support for the ovule gene network arising at least partly from a seedless vascular plant sporangium-bearing organ. In response to Reviewer 2's query, we have more clearly highlighted this point in the Discussion (**lines 582-584, 600-603**).

This also ties in with the author's response "We respectively disagree with Reviewer 2's assertion that the ovule is simply a homologous structure with fern sporangia with a gametophyte retained"

While I understand the authors point and perspective about providing a molecular developmental analysis, it seems quite far-fetched to propose that the ovule—and thus the embryo—is not homologous across land plants. That is not to say that it isn't true, evolution does not follow our conceptions of "reasonable" or "unreasonable," but we would then need to ask much deeper and broader questions about the evolution of sex in plants (e.g., how does one evolve a novel egg producing structure, how does meiosis or fertilization arise independently in different cell lines), and it also raises questions about interpretations of the nucellus (previously understood as the sporangium).

- We thank Reviewer 2 for making this point. We think that there has been a misunderstanding based around our use of the phrase 'homologous structure' in the context of that sentence. In hindsight we can see how this could be misinterpreted, and we apologise for this ambiguity. To clarify, we did not intend to suggest that ovules are polyphyletic. To make this absolutely clear we have edited the manuscript to specifically state that seeds have a single evolutionary origin (**line 53**).

- Instead, our intention was to highlight that we disagree with the premise that, in developmental terms, the ovule can be considered simply as a sporangium with a gametophyte retained. We certainly do not disagree that the ovule probably evolved from a sporangium-bearing organ (see above), but ovule development is now significantly different (and more complex) from that, and the mechanism(s) through which those developmental differences arose still needs to be explained. We believe that this knowledge gap is what this manuscript makes progress towards resolving.

This statement that the authors make in the response needs further clarification and articulation in the manuscript. How do their data support this claim? "we believe we demonstrate a transfer of gene network from the gametophyte to the sporophyte phase, and that these are responsible for developmental changes in the sporophyte tissues that give rise to the ovule body plan."

- We thank Reviewer 2 for highlighting the need to explain this further. We believe this claim is supported by the following evidence:
  1. We identify enrichment of a population of genes between the fern post-fertilization archegonium and the angiosperm ovule that is statistically greater than with other non-reproductive angiosperm tissues (**Fig. 7,8**), indicating conservation of gene networks between the ovule and the post-fertilization archegonium.
  2. We further provide evidence that shows this enrichment is not simply due to the presence of a female gametophyte within the ovule, but instead there is enrichment of these post-fertilization archegonium genes (627) exclusively in the pre-meiotic (ie. sporophytic) ovule tissues, including the nucellus (**Fig. 9**).
  3. We do not detect any similar significant enrichment of genes directly between the fern diploid sporangium-bearing axis (the sporophyll) and the ovule (**Fig. 7**), indicating that this ovule gene network is not also fully-present in the sporophyll, and thus (we deduce) not in the ancestral sporangium-bearing axis from which ovule sporophytic tissues derived. In response to the Reviewer's concern we have edited this discussion to more clearly highlight this point (**lines 580-588**).
  4. We do indirectly detect enrichment of notch genes conserved with the ovule (116) in the sporophyll primordium. This suggests that some part of the ovule gene network arose from the pre-existing gene networks of the sporophyll, but comparison of this set of genes with those directly conserved between ovule sporophytic tissue and post-fertilization archegonium shows that many of gametophyte-enriched ovule genes are not already present in the sporophyll primordia. In response to the Reviewer's concern, to better articulate this point in the manuscript we have included the analysis described above showing this comparison (**Supplementary Data 11**) and have highlighted it in the Results (**lines 456-461, 458-488**) and discuss its evolutionary implication in the Results and Discussion sections (**lines 588-590**).
  5. From the evidence outlined above we infer that the expression of some genes in the ovule sporophytic tissues potentially arose by their expression domain transferring (or at least expanding) from the post-fertilization archegonium to the ancestral sporophytic axis. In response to the Reviewer's concern we have now elaborated this point in the Discussion (**lines 592-603**) and further explained how this fits with other observations about gene regulation and the observed evolutionary history of seed-based reproduction (**lines 605-617**).

Line edits

Abstract
Line 38: before “found” add “we”

- We thank Reviewer 2 for spotting this- due to other edits requested this sentence has been further rephrased to meet the Abstract word limit (**lines 37-38**).

Line 46: I think one more sentence would benefit the abstract here. Particularly, a focus on what this means for the origin and evolution of the ovule. My interpretation is that these data support the homologous hypothesis that the ovule is derived from a retention of a single megasporangium on a megasporophyll (e.g., gene network similarities of both are found). So, just rounding out what the authors think will be helpful.

- We thank Reviewer 2 for this suggestion. We have edited the final sentence of the Abstract to better explain what our data suggests about the evolutionary origins of the ovule whilst remaining within the Abstract word limit. We now make clear that our results support the ovule’s origin from a sporangium-bearing axis, but that the development of that sporangium-bearing shoot became modified into a new form by expression of genes originally associated with post-fertilization archegonium development (**lines 42-46**). From our data we cannot make any claims over whether this axis was already a megasporangium/megasporophyll or not. We hope that this summary strikes an appropriate balance between the perspectives of evolutionary homologies and the mechanistic developmental changes underlying the evolution of plant form.

Introduction

Line 61: the parenthetical “(‘ovules’ before fertilization)” may confuse some readers. I understand that the authors mean that before fertilization seeds are called ovules, but some may interpret this as them meaning seeds are ovules before fertilization.

- We thank Reviewer 2 for highlighting this potential confusion. In response we have expanded this parenthesis to ‘(referred to as ‘ovules’ before fertilization)’ to clarify that seeds and ovules are the same structure in different stages of development. **(line 61**)

Line 63: The nucellus is also a nutritive tissue in some lineages

- We thank Reviewer 2 for highlighting this, and have now included this point (**lines 66-68**).

Line 72–75: the independent origin of fern leaves does not preclude the ancestral telomic axis bearing a sporangium as being the ancestor of the ovule.

- We thank Reviewer 2 for highlighting this. In the interests of clarity we have now included the point that fern fronds might be homologous to ancestral axes (**lines 74-75**).

Line 86: be careful not to perpetuate the trope of the hermaphroditic fern gametophyte. See Haufler, Christopher H., et al. "Sex and the single gametophyte: Revising the homosporous vascular plant life cycle in light of contemporary research." BioScience 66.11 (2016): 928-937.

- We thank Reviewer 2 for highlighting this- in response we have included this reference and edited this sentence to highlight the possible diversity of gametophyte morphology and reproductive strategies and to make it 100% clear that we are specifically describing the reproductive mode of our experimental model, *Ceratopteris richardii* (**lines 88-90**).

Discussion

Line 529: I suggest using a more phylogenetically-minded term than “basal”

- We thank Reviewer 2 for highlighting this, and have edited the sentence to avoid the term ‘basal’ (**lines 515-516**).

Line 549–550: typo “candidates genes”

- Thank you- as part of our redrafting of the Discussion this has been removed (line 534-536).

Reviewer #3 (Remarks to the Author):

I thank the authors for having aptly dealt with my comments. I have no further remarks.

- Thank you- we are glad that we were able to address all of Reviewer 3’s comments to a satisfactory standard.

PLACKETT & CATONI 2026- RESPONSE TO REVIEWERS' COMMENTS

Reviewer #2 (Remarks to the Author):

The authors have addressed most of my concerns. I think where we currently lie is in a disagreement of how to frame the work and whether comparisons between extant homosporous fern reproduction and seed plants are meaningful to make assessments about the transitions hat occurred in the common ancestor of seed plants. That is fine. I believe that the questions the authors ask are inherently interesting and I do appreciate the attempt of opening the "black box" of development to try to find a mechanistic molecular explanation for the origin of the ovule. I hope this work can be a foundation and springboard for future explorations into this question.

- We thank Reviewer 2 for this clarification, and we are sincerely grateful to them for their efforts in helping us to put our results into the appropriate context within current literature and thinking within the field of seed evolution.

A few suggestions to help the authors make the manuscript more readable.

Line 43–46: "Our findings support a novel mechanistic model to explain the origin of the ovule from a sporangium-bearing axis, with the developmental modifications underpinning the ovule body plan arising from gene networks in the seed-like post-fertilization archegonium that became expressed during development of that sporangium-bearing axis." this statement is quite confusing. I understand what the authors are articulating, but it took me a few reads. I suggest rewording as follows: "Our findings support a novel mechanistic model to explain the origin of the ovule from a sporangium-bearing axis, whereby gene networks in the post-fertilization archegonium of seed-free ancestors were heterotopically expressed during the development of the seed-plant sporangium-bearing axis."

- We thank Reviewer 2 for this suggestion. We have incorporated their rewording with minor modification to fit within the Abstract word limit (**lines 36-42**).

Line 81-85: I may suggest flipping heterospory and endospory as it is likely that heterospory evovles prior to endospory (e.g., we see many heterosporous ferns that are not endosporous (Marsilieaceae), but we do not see any endosporous plants that are homosporous.

- We thank Reviewer 2 for this suggestion. We have edited this paragraph within the introduction to discuss the evolution of heterospory before endospory (**lines 75-84**). The order of citations/references has been updated to correspond with this change.

Line 503–505: it is not necessarily that the presence of similar gene expression is a smoking gun for heterotopy and leveraging ancestral GRN. I would suggest rewording.

- We thank Reviewer 2 for highlighting this point. We agree and have edited this paragraph to better clarify our intended meaning, namely that our results support the hypothesis that reproductive gene networks are conserved between ferns and angiosperms (**lines 489-491**) and that this raises the potential of identifying changes to ancestral GRN important to the evolution of the ovule (**lines 494-496**).
