# Supplementary material for: The post-fertilization archegonium gene network of seedless plants contributed to the origin of seeds
Source: Nat Commun. 2026 Jun 19;17:7751. doi: 10.1038/s41467-026-74577-w (PMC13434465; doi:10.1038/s41467-026-74577-w)
Supplement: Supplementary file 3 [file 41467_2026_74577_MOESM1_ESM.pdf]

The post-fertilization archegonium gene network of seedless plants contributed to the origin of seeds

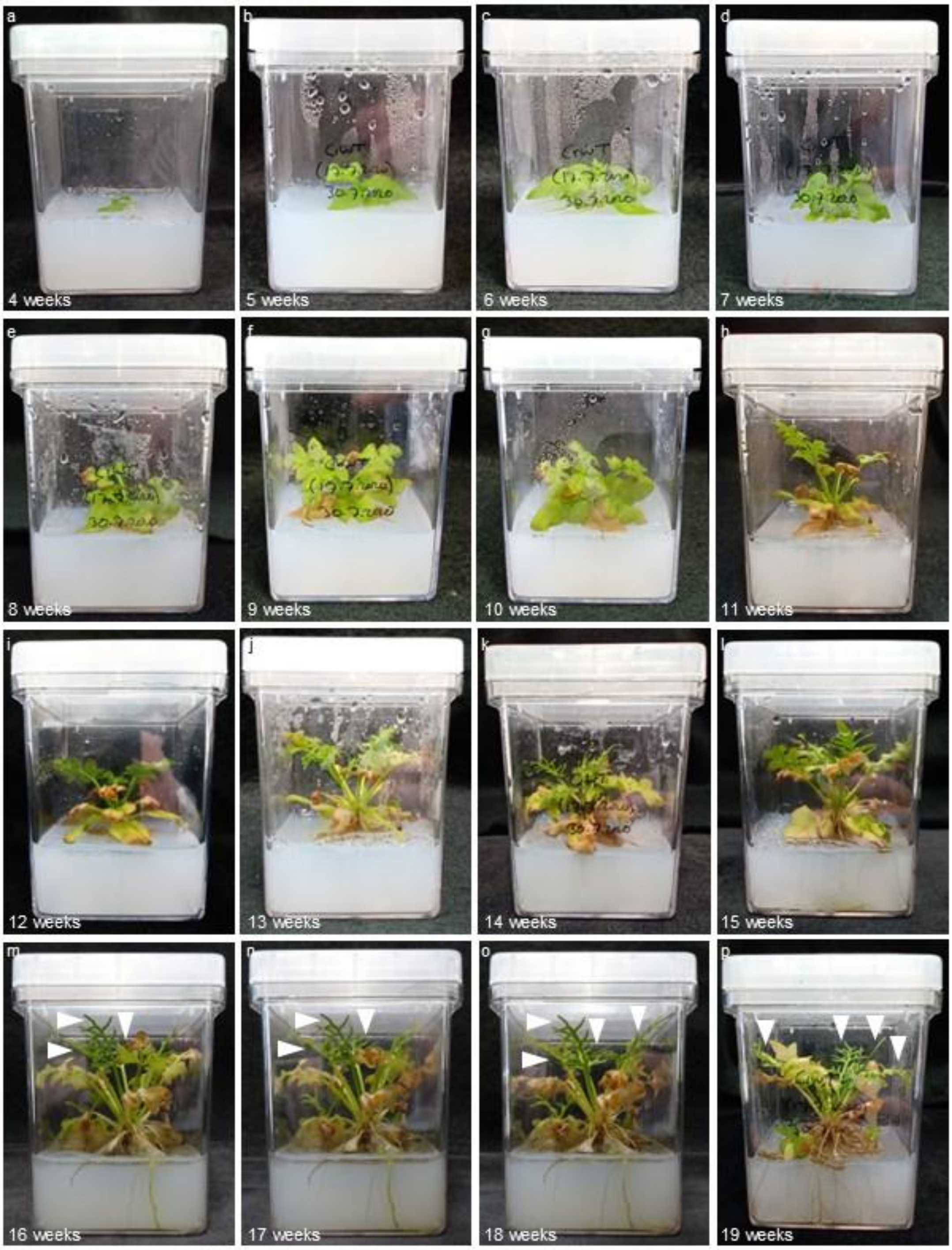

Supplementary Fig. 1. Growth of the *Ceratopteris* sporophyte under tissue culture conditions. **a-p**, Developmental series of the *Ceratopteris* sporophyte grown under sterile tissue-culture for reproductive characterisation, shown at weekly intervals from four weeks old (**a**) until tissue harvesting for mRNA-seq analysis at 19 weeks (**p**). Sporophylls are morphologically distinct and discernible from preceding vegetative fronds by eye (white arrowheads). Experiment repeated 9 times.

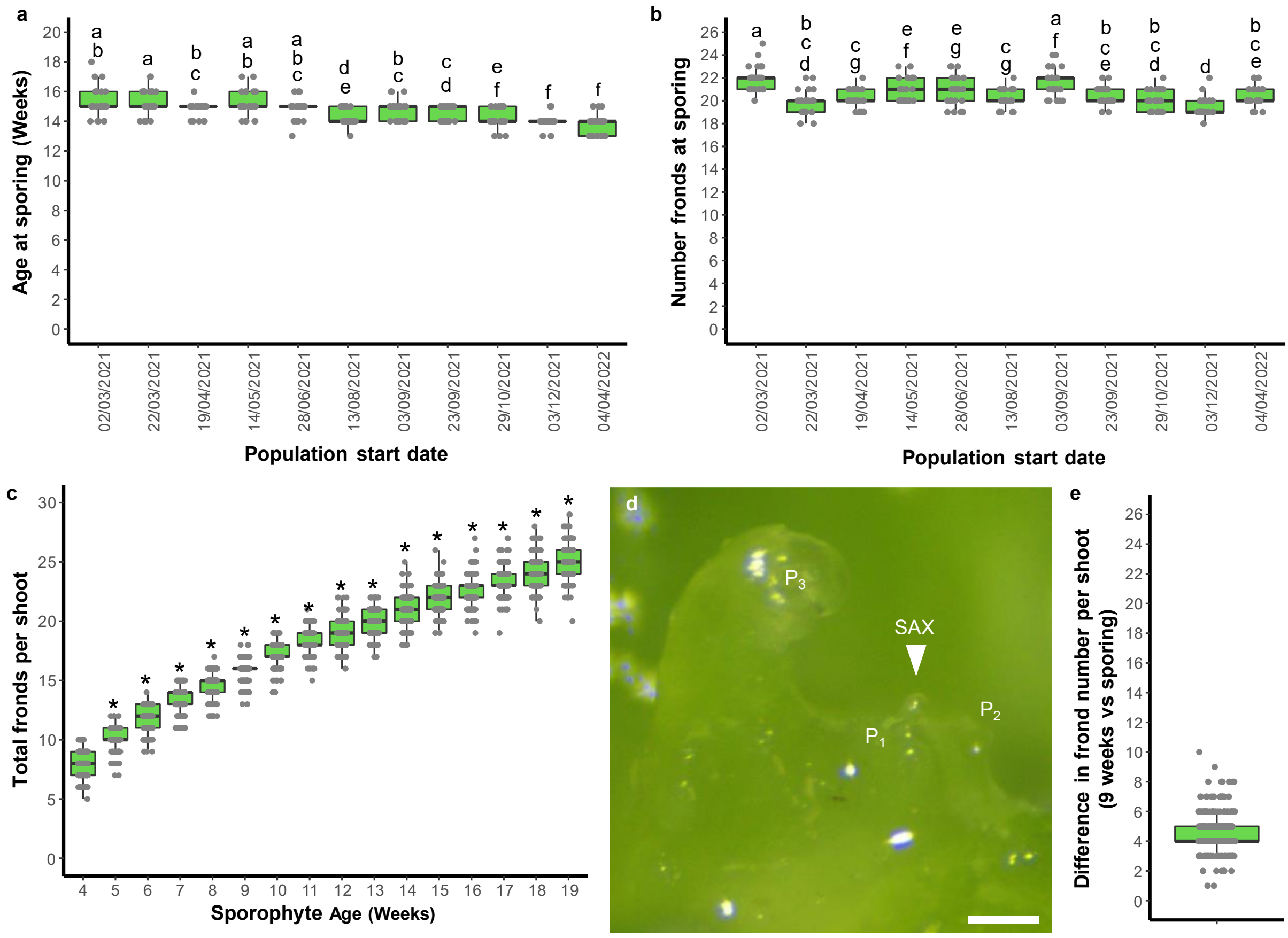

Supplementary Fig. 2. Reproducibility of the *Ceratopteris* sporing transition.

a-b, Mean sporing time within each population grown, measured by chronological age (**a**) and frond position of the first sporophyll (**b**). Letters denote statistical similarity, with different letters indicating a significant difference ($P_{adj} < 0.05$) between experimental populations.

c, The total number of fronds per sporophyte during development, confirming that shoot development remained active throughout the growing period. Asterisks denote a significant difference ($P_{adj} < 0.05$) in total frond number compared to the preceding week.

d,**e,** At 9 weeks old the shoot apical region (**d**) contains the shoot apex (SAX) and sequentially-developing primordia (P_1-P_3) not visible to the naked eye and so not included in frond counts in (**a-c**). Scale bar = 200 μm. Comparing the number of visible fronds at 9 weeks old and the corresponding first sporophyll position per plant (**e**) found a mean difference significantly greater than 3 ($P < 0.0001$), indicating that 9 week shoots have not transitioned to sporing.

Boxplots (**a-c**, **e**) denote the data median and interquartile range (box) ±1.5x the interquartile range (whiskers). *n* = 11 (**a-c**), 271 (**h**). Pairwise comparisons were performed using two-tailed Mann-Whitney tests (**a-c**, two-tailed; **e**, one-tailed [greater than]) on log10-transformed (**a**,**b**) and square root-transformed data (**c**), respectively, adjusted for multiple comparisons (Benjamini-Hochberg method). Statistical analysis and adjusted P-values are given in **Supplementary Data 1**. Source data are provided as a **Source Data** file.

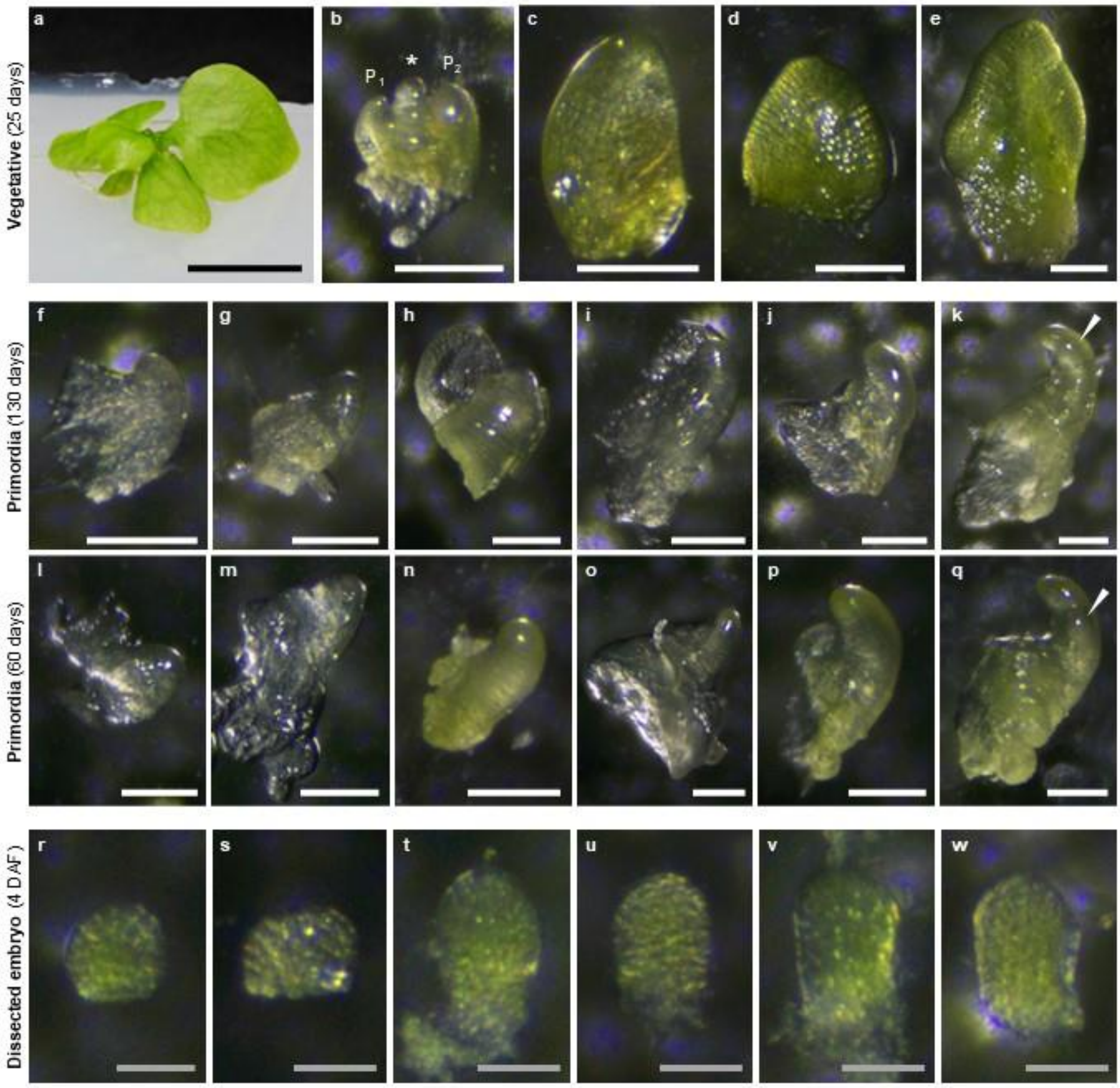

Supplementary Fig. 3. Additional mRNA-seq samples and developmental ranges.

a-e, At 25 days old *Ceratopteris* sporophytes generate fronds with a simple (spade-like) morphology (**a**). Two samples were dissected for inclusion in mRNA-seq analysis: the shoot apical region (Apx) (**b**) comprising the shoot apex (asterisk) and recently-emerged primordia (P_1, P_2), and the earliest dissectible frond primordium within the enclosed shoot apical region (Prm), encompassing a range of sizes (**c-e**).

f-q, Primordium developmental stages encompassed by Prm samples at 130 days (**f-k**) and 60 days (**l-q**). Development within samples at both ages ranged from initial organ outgrowth to curling and the initiation of lateral pinnae (arrowheads) but excluded complete fiddleheads.

r-w, Embryo development stages encompassed by dissected 4 DAF dissected embryo (Dem) samples.

Scale bars = 10mm (black), 200 µm (white) and 100 µm (grey).

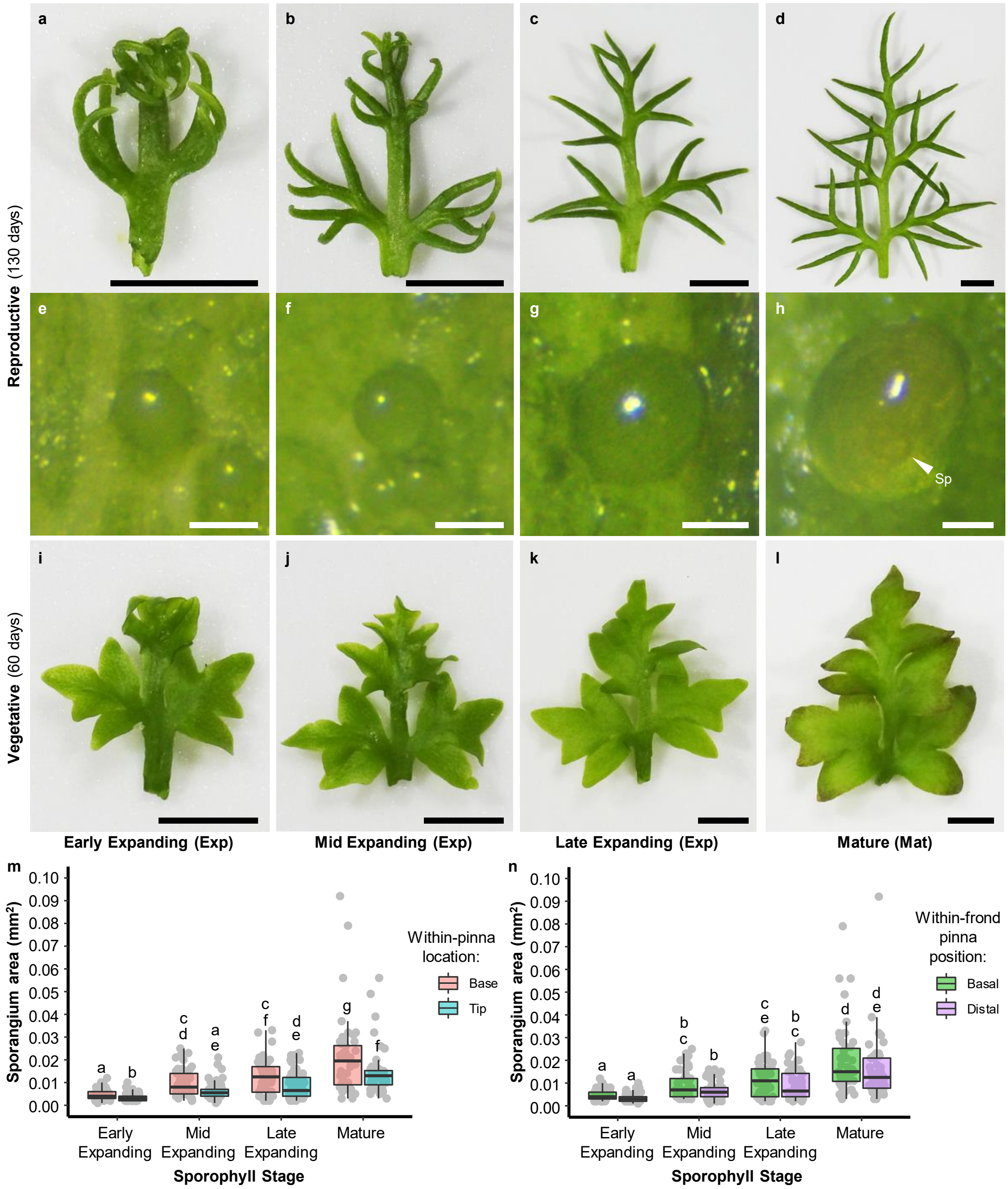

Supplementary Fig. 4. Characterisation of *Ceratopteris* sporangium development during sporophyll expansion.

a-h, Expanding sporophylls and representative sporangia, divided into sub-stages based on frond size: ‘early expanding’ (**a**,**e**), where fronds are still unfurling; ‘mid expanding’ (**b**,**f**), between newly-unfurled fronds and approximately 50% of final size; ‘late expanding’ (**c**,**g**), between approximately 50% and 75% of final size and ‘mature’ (**d**,**h**) where fronds are of similar size to those immediately preceding it. Post-meiotic sporangia containing individual spores (Sp) were visible in mature fronds only. Experiment repeated 3 times.

i-l, Expanding vegetative fronds from 60d sporophytes, following the sub-stages established for sporophylls (**a-d**). Scale bars = 5 mm (black) and 100 μm (white). Experiment repeated 3 times.

m,n, Sporangium planar area across sub-stages of frond expansion, comparing sizes within individual pinnae (**m**) and between pinnae within an individual frond (**n**). *n* =24 (4 sporangia per pinna location, measuring 6 independent sporophytes). Boxplots denote the data median and interquartile range (box) ±1.5x the interquartile range (whiskers). Letters denote statistical similarity, with different letters indicating a significant difference ($P_{adj} < 0.05$) between groups. Pairwise comparisons were performed using two-tailed Mann-Whitney tests on log10-transformed data, adjusted for multiple comparisons (Benjamini-Hochberg method). Statistical analysis and adjusted P-values are given in **Supplementary Data 1**. Source data are provided as a **Source Data** file.

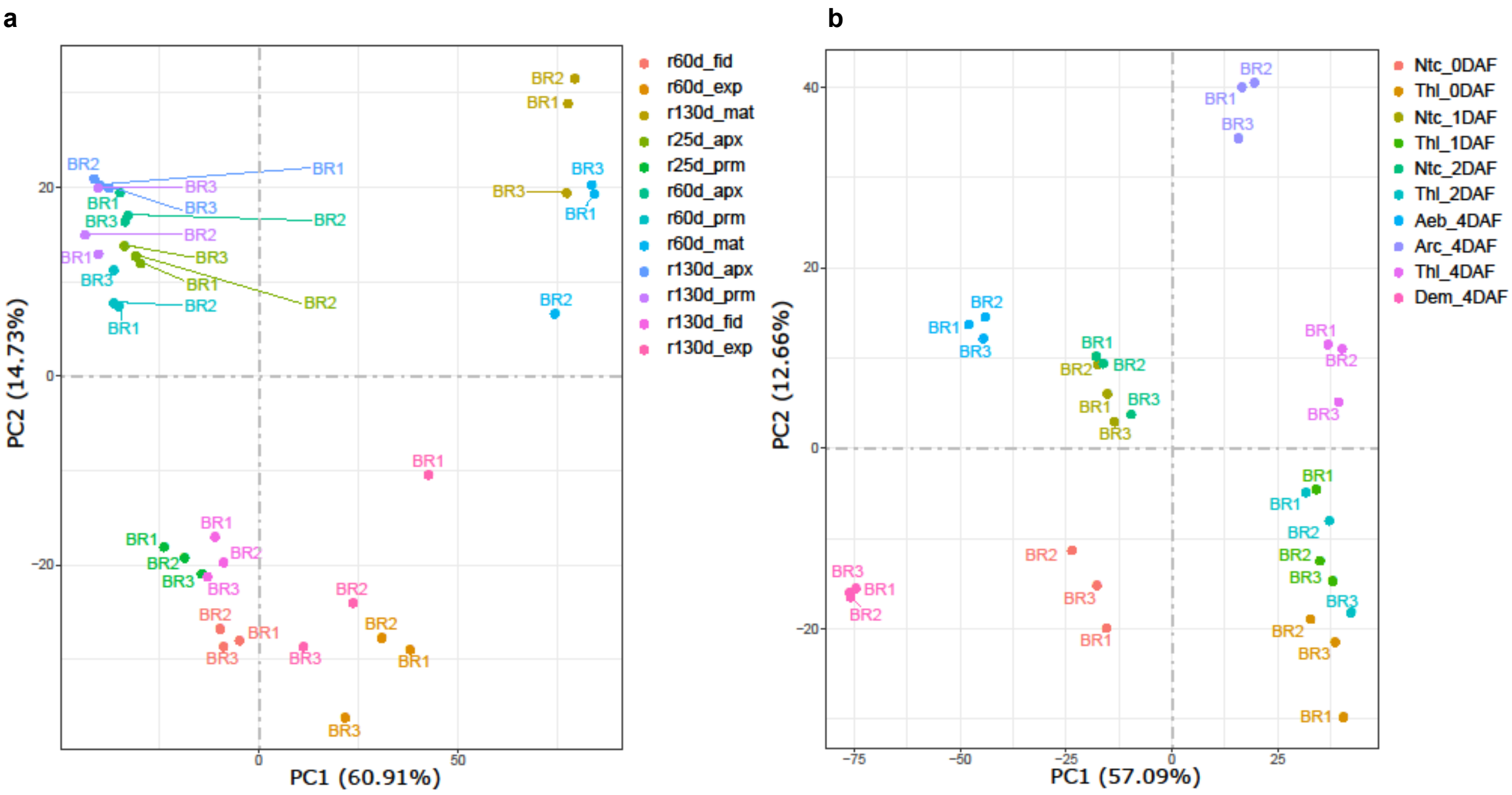

Supplementary Fig. 5. *Ceratopteris* mRNA-seq PCA analysis.

a, PCA plot of sporophyte mRNA-seq samples. PC1 separates samples by frond developmental stage, PC2 separates vegetative and reproductive samples within each stage. Samples predominantly group by tissue type, with biological replicates typically cluster more closely together than with replicates of other samples. The exception to this are 25d primordia samples (r25d_prm) which cluster more closely with fiddleheads than other primordia.

b, PCA plot of gametophyte mRNA-seq samples. PC1 separates notch and embryo-bearing samples from thallus samples, with embryo-less 4DAF notch tissues (Arc_4DAF) falling between. PC2 separates samples progressively by age within tissue types (with 1DAF and 2DAF overlapping), with the exception of dissected embryos (Dem_4DAF). Biological replicates typically cluster more closely together than with other samples, with the exception of 1DAF vs 2DAF within the notch and thallus. Gene expression values within each sample (FPKM) are provided as a **Source Data** file.

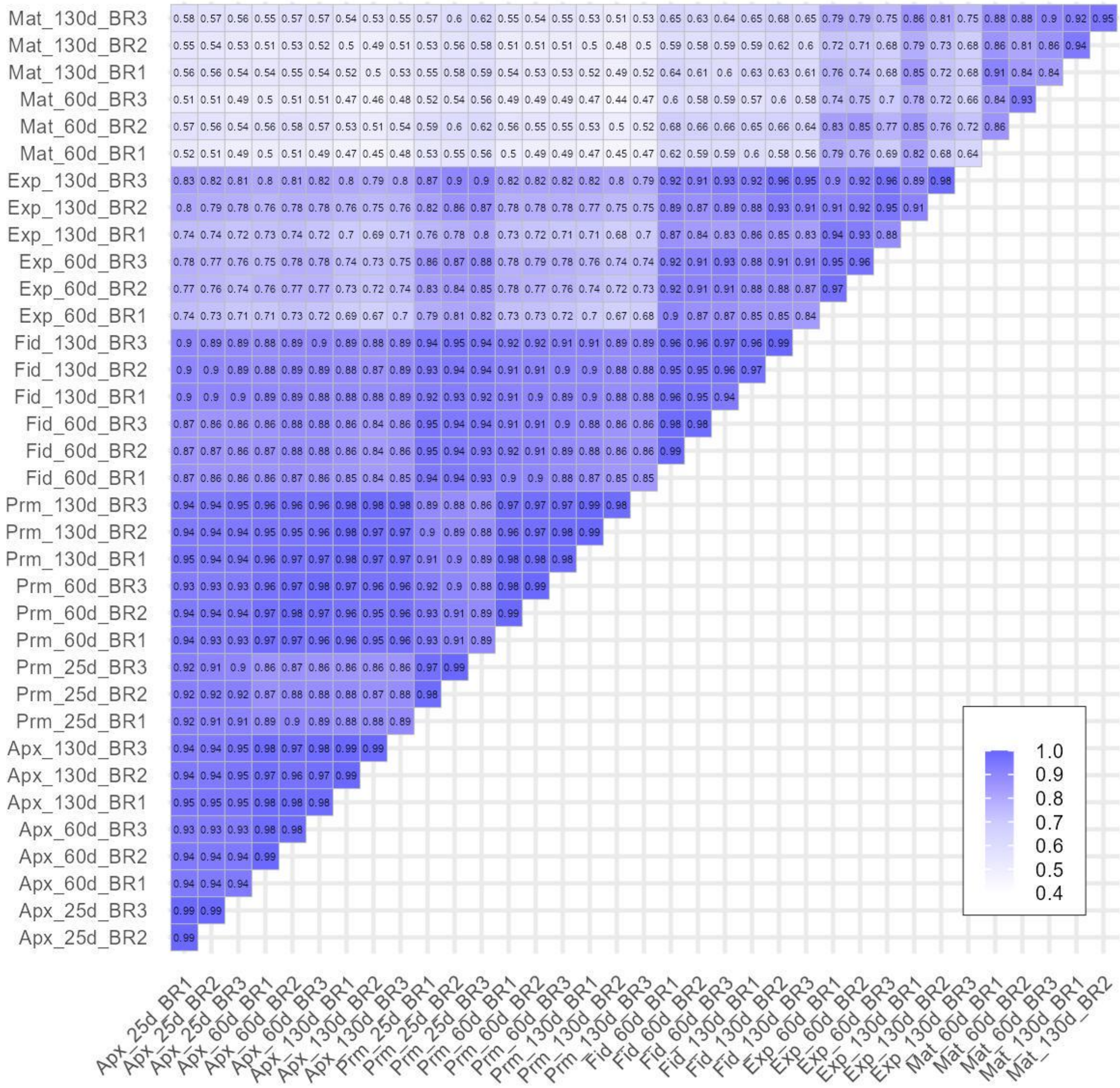

Supplementary Fig. 6. *Ceratopteris* sporophyte mRNA-seq sample correlation data. Pairwise correlation of sporophyte mRNA-seq samples, grouped by biological replicate, then by frond developmental stage in order of frond development. Colour intensity denotes increasing correlation, as shown. Samples typically show the highest correlation between biological replicates, and then between vegetative and reproductive samples within the same frond developmental stage, with the exception of 25d primordia.

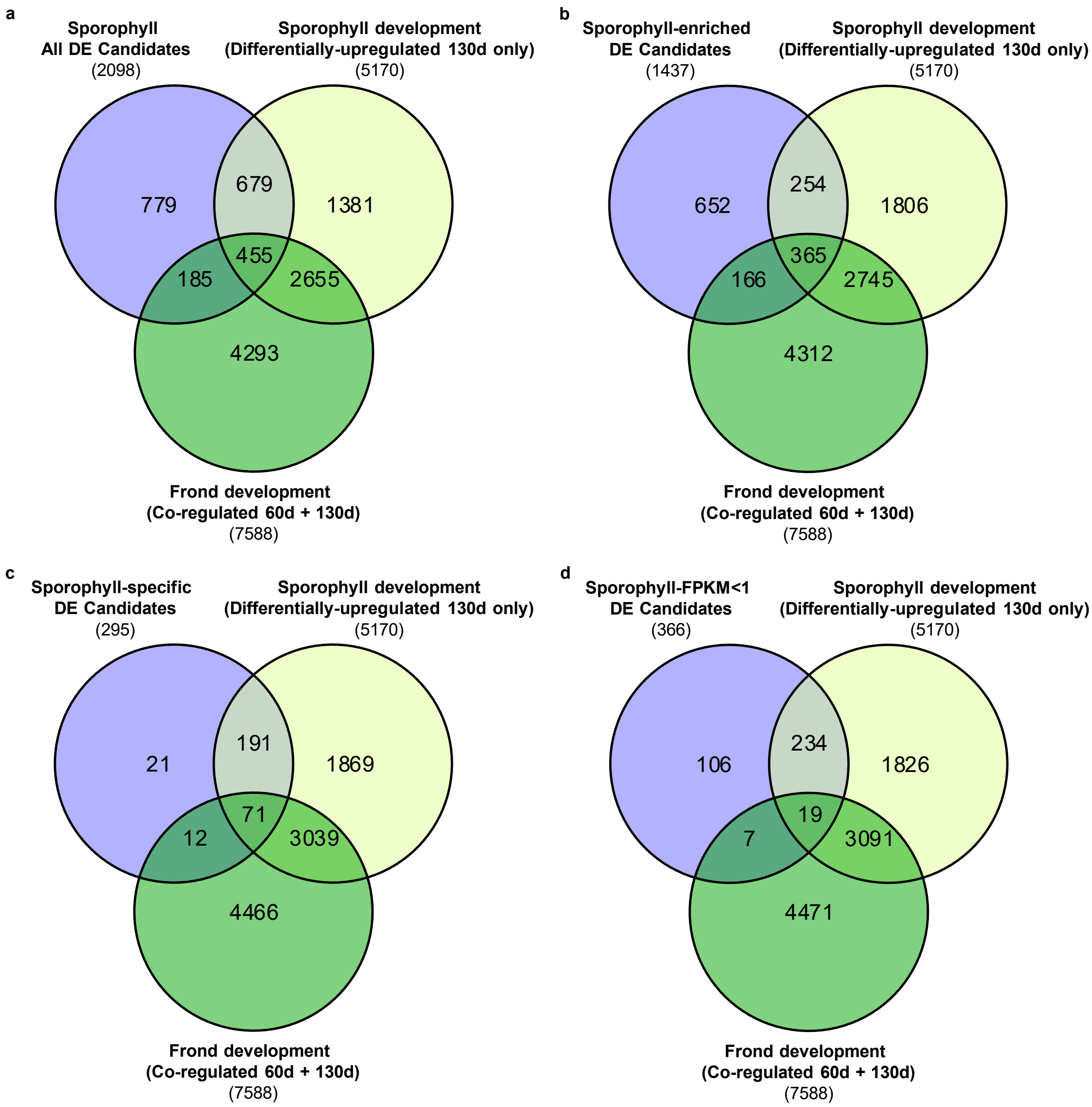

Supplementary Fig. 7. Overlap between sporophyll reproductive candidates and genes differentially-regulated during sporophyll and frond development.

Venn comparison of reproductive candidates identified via differential expression (DE) directly between reproductive and vegetative samples within frond developmental stages with genes showing differential upregulation between at least two stages of frond development specifically in sporophylls (130d fronds) and genes showing co-upregulation between the same stages of vegetative frond and sporophyll development (60d + 130d). Venn diagrams show the comparison of all DE candidates (**a**) and the sporophyll-enriched (**b**), sporophyll-specific (**c**) and sporophyll-FPKM<1 subsets (**d**) identified in **Fig. 1q**. Numbers in brackets represent the total number of genes within each dataset. Source data are provided as a **Source Data** file.

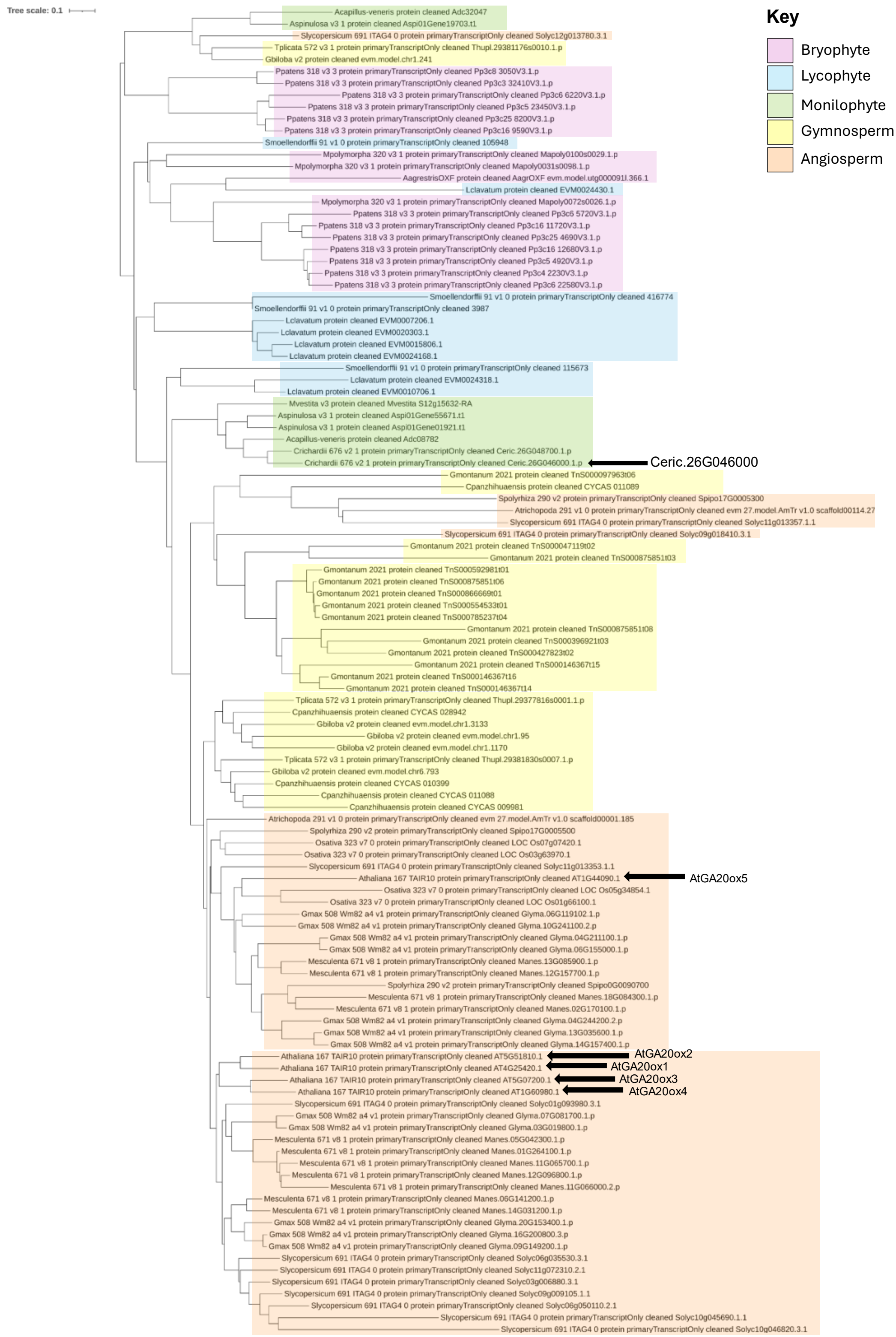

Supplementary Fig. 8. Phylogenetic gene tree of land plant putative *GA20ox* gene orthologs. Phylogenetic tree derived from Orthofinder, showing all genes within Orthogroup 661. Clades are colour-coded according to their phylogenetic lineage (angiosperm, gymnosperm, monilophyte, lycophyte, bryophyte). The positions of *Arabidopsis GA20ox* genes *AtGA20ox1-5* and the putative *CrGA20ox* (Ceric.26G046000) are indicated by black arrows.

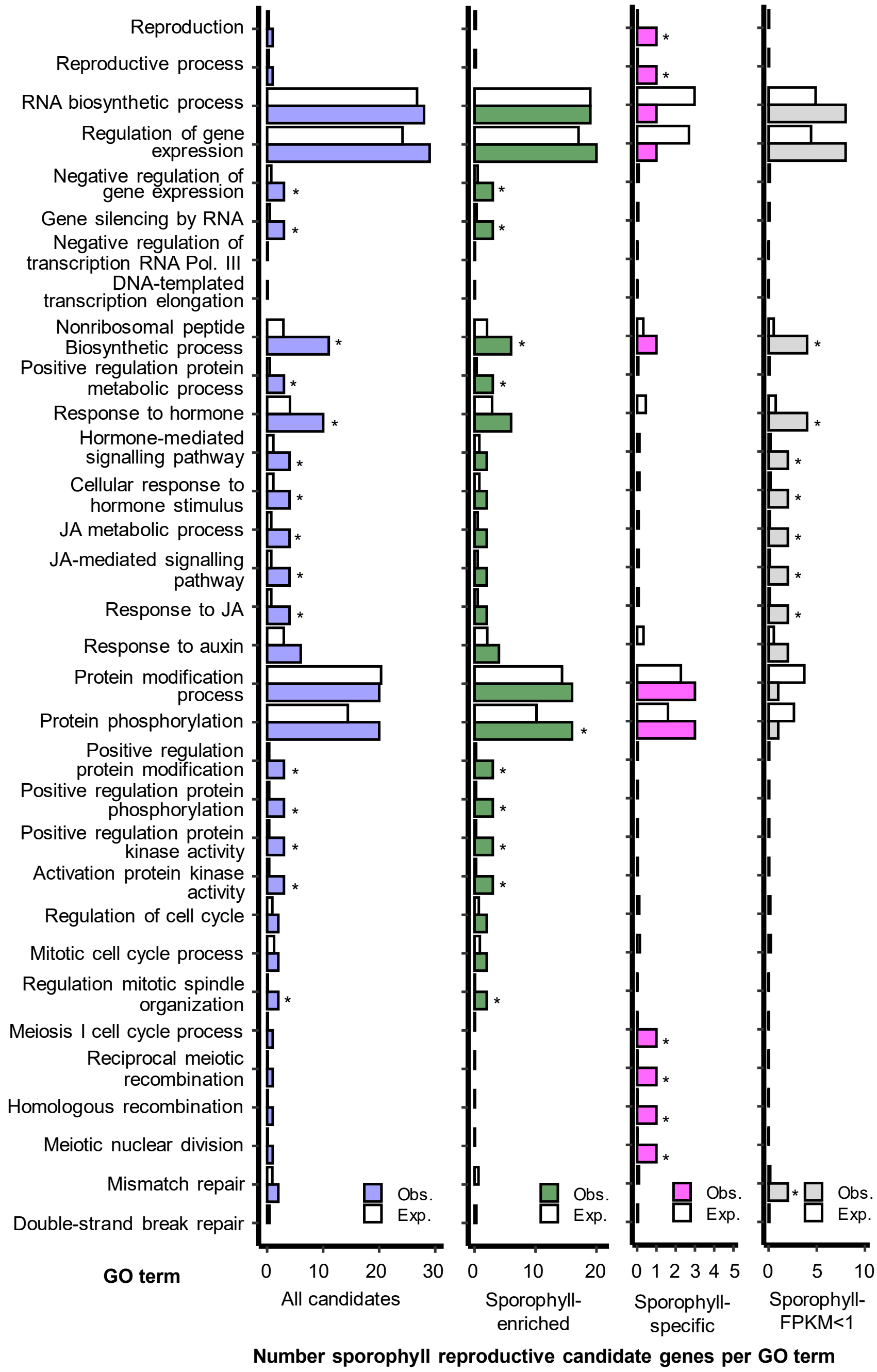

Supplementary Fig. 9. Distribution of enriched GO terms across sporophyll DE candidate expression categories.

Frequency of sporophyll DE reproductive candidate genes with individual GO terms observed against the frequency expected based on the whole genome, comparing all candidates vs sporophyll-enriched sporophyll-specific and sporophyll-FPKM<1 expression categories. Barplots show the observed ('Obs.') and expected ('Exp.') gene frequencies per GO term (*n* = 1). Enrichment was tested using Fisher's Exact test (one-sided, greater than). Asterisks denote significant enrichment ($p < 0.05$) in genes with that GO term compared to the expected frequency. Exact P-values are given in **Supplementary Data 5**. Source data are provided as a **Source Data** file.

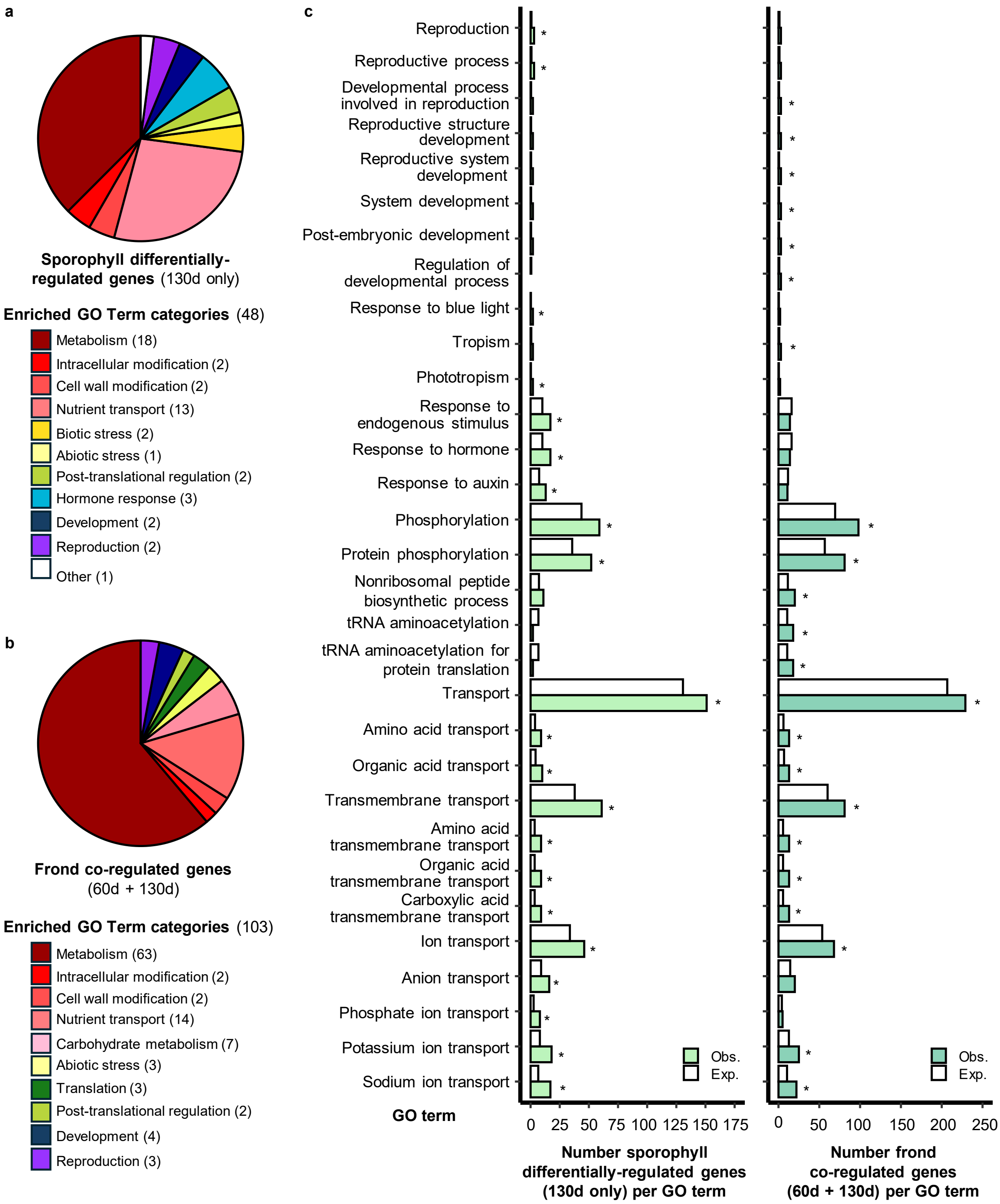

Supplementary Fig. 10. GO term enrichment analysis of sporophyll development genes. Summary of enriched GO terms (**a**,**b**) and the frequency of sporophyll DE reproductive candidate genes with individual GO terms against the frequency expected based on the whole genome (**c**) in genes differentially-upregulated during sporophyll development and genes co-upregulated during sporophyll and vegetative frond development. Barplots show the observed ('Obs.') and expected ('Exp.') gene frequencies per GO term (n = 1). Enrichment was tested using Fisher's Exact test (one-sided, greater than). Asterisks denote significant enrichment ($p < 0.05$) in genes with that GO term compared to the expected frequency. Exact P-values are given in **Supplementary Data 5**. Source data are provided as a **Source Data** file.

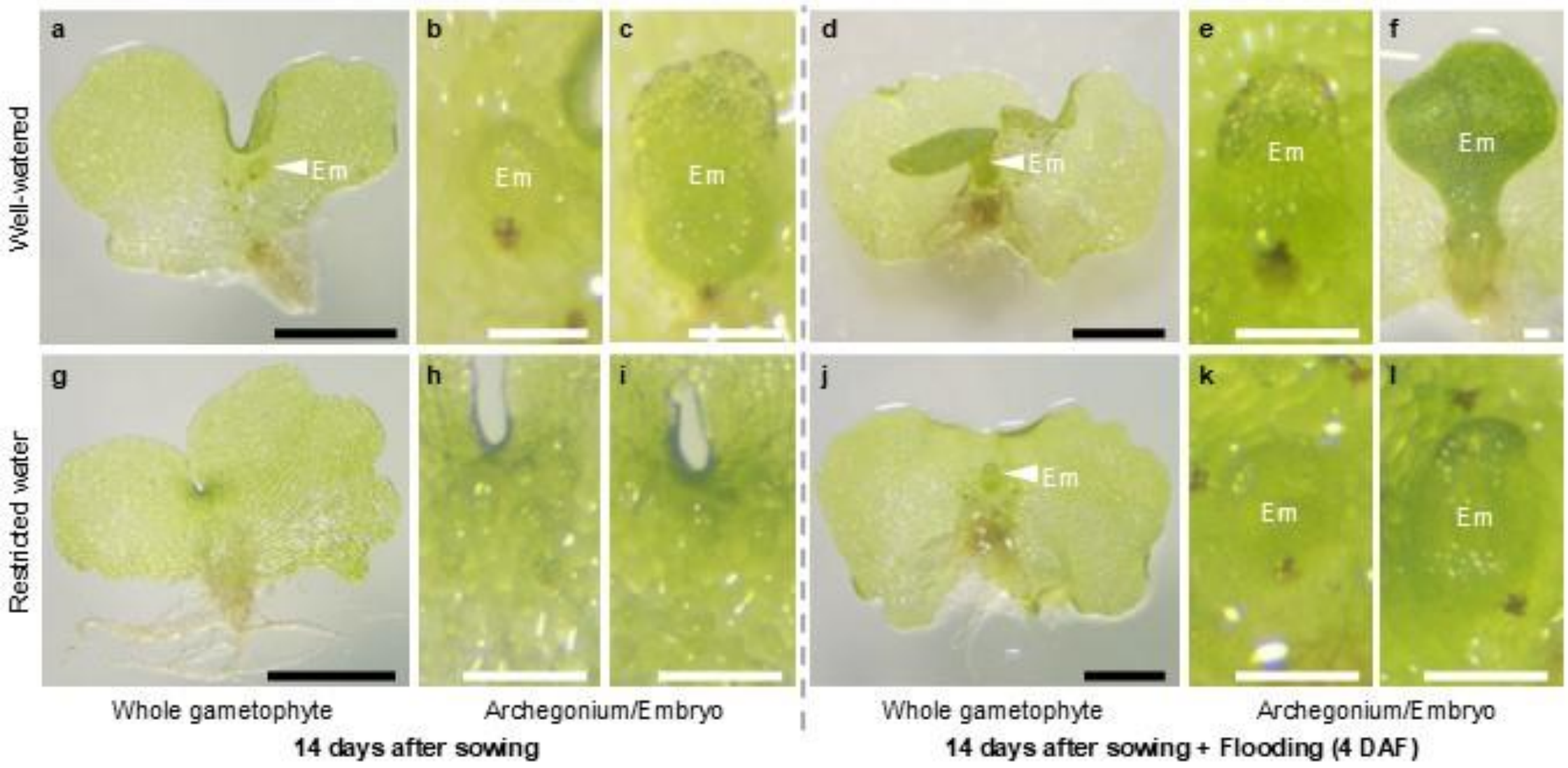

Supplementary Fig. 11. *Ceratopteris* embryo development is synchronised through restricted watering.

Comparison of gametophyte and embryo morphology 14 days after sowing between well-watered (**a-f**) and restricted water growth conditions (**g-l**) in unflooded populations 14 days after sowing (**a-c**, **g-i**) and 4 days subsequently after flooding (**d-f**, **j-l**). In well-watered populations, embryos (Em) were already visible on gametophytes at 14 days after sowing (**a**) with a range of different developmental stages detectable (**b,c**). This disparity persisted in the population after flooding (**d-f**). Under restricted water, embryos were not visible 14 days after sowing (**g-i**) and after flooding the developmental range of visible embryos was much closer (**j-l**). Scale bars = 1 mm (black), 200 μm (white). Experiment repeated 3 times.

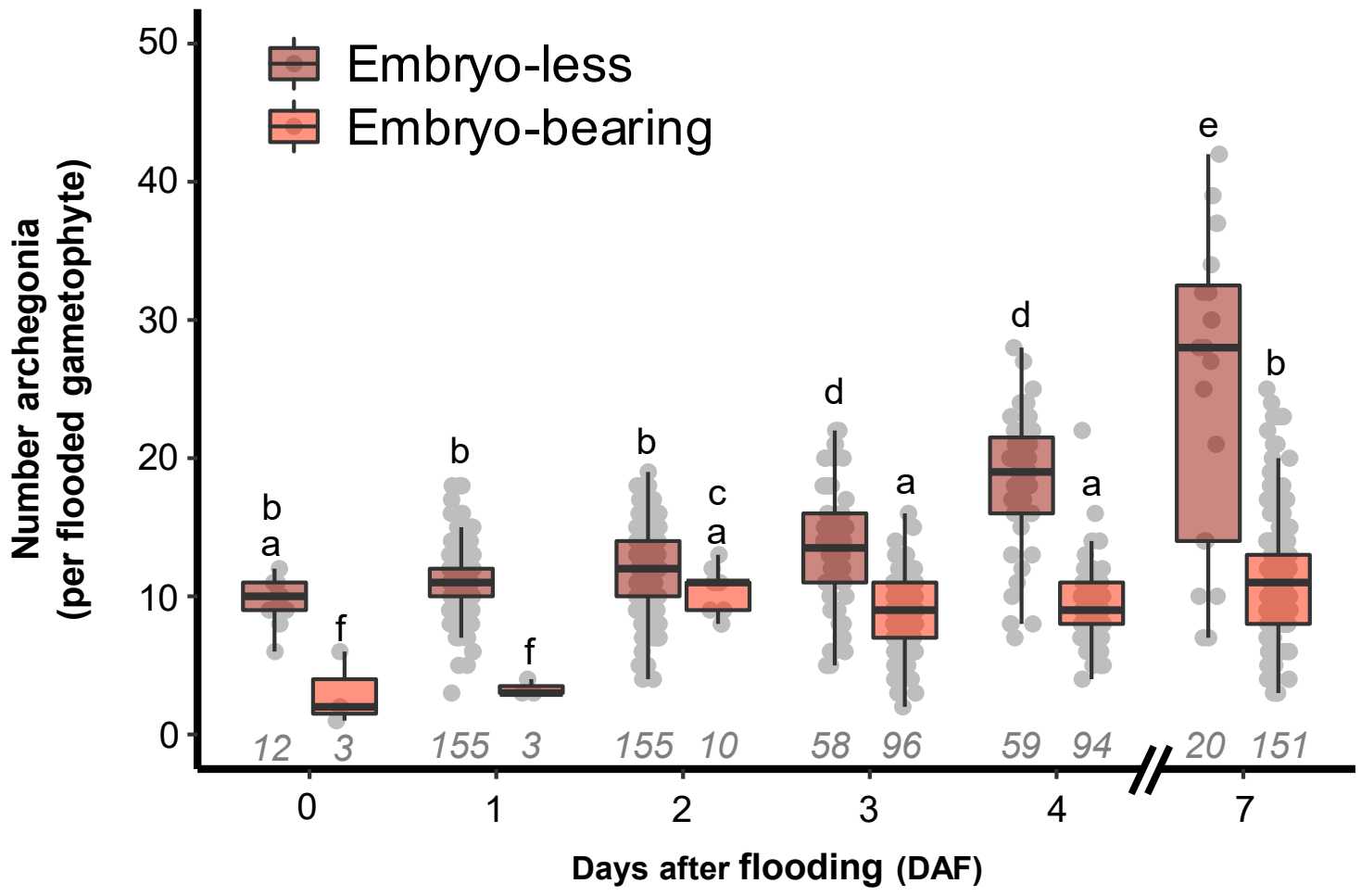

Supplementary Fig. 12. Characterising *Ceratopteris* notch shut down after fertilization. Number of archegonia per flooded gametophyte per DAF, comparing between flooded gametophytes with visible embryos ('embryo-bearing) and those without ('embryo-less'). Archegonium production stopped increasing in embryo-bearing gametophytes after 2 DAF but continued in embryo-less individuals, suggesting embryo-dependent notch meristem shutdown. Boxplots show the median and interquartile range (box) ±1.5x interquartile range (whiskers). Letters denote statistical similarity, with different letters indicating a significant difference ($P_{adj} < 0.05$) between groups. *n* is given per embryo status vs DAF combination (grey italics). Pairwise comparisons were performed using two-tailed Mann-Whitney tests on log10-transformed data, adjusted for multiple comparisons (Benjamini-Hochberg method). Statistical analysis and adjusted P-values are given in **Supplementary Data 6**. Source data are provided as a **Source Data** file.

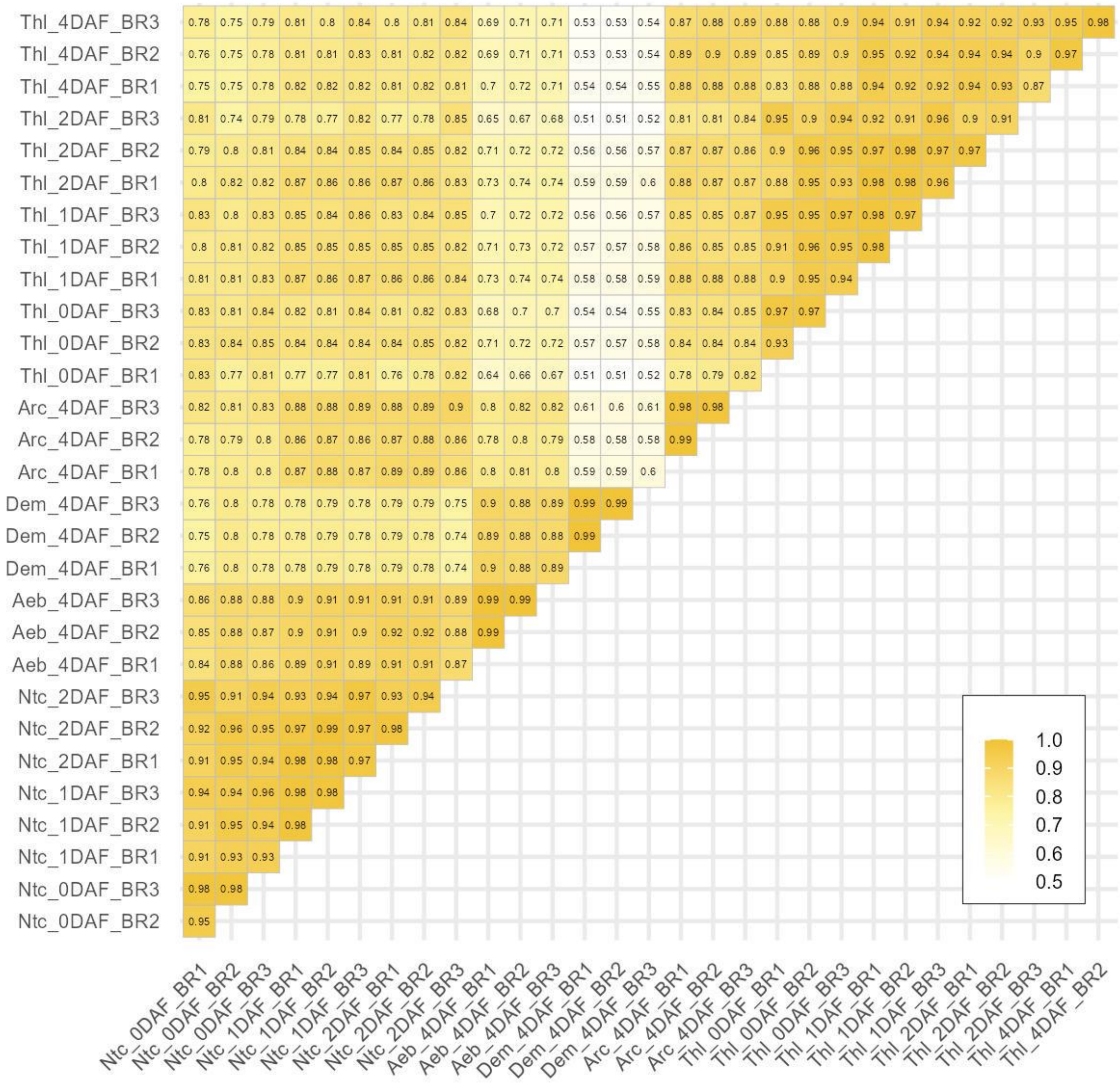

Supplementary Fig. 13. *Ceratopteris* gametophyte mRNA-seq sample correlation data. Pairwise correlation of gametophyte mRNA-seq samples, grouped by biological replicate, and then by each developmental stage within notch (Ntc) and thallus (Thl) groups. Colour intensity denotes increasing correlation, as shown. Samples typically show the highest correlation between biological replicates, and then within notch and thallus tissue types, with the exception dissected embryos (Dem), which correlate closely with embryo-bearing archegonia (Aeb) but are distinct from all other samples.

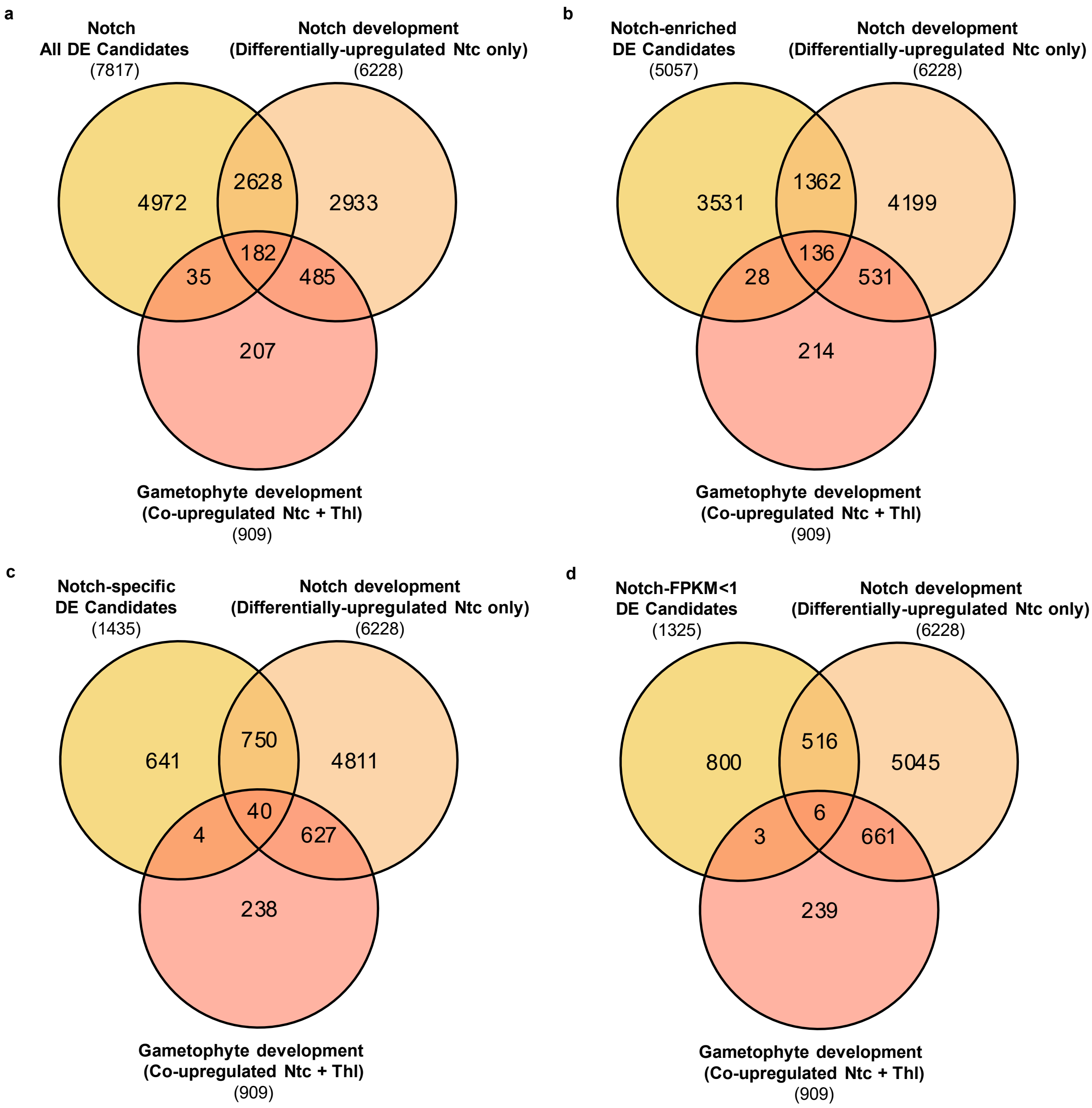

Supplementary Fig. 14. Overlap between differentially-expressed notch reproductive candidates and genes differentially-regulated during notch development.

Venn comparison of reproductive candidates identified via differential expression (DE) directly between reproductive and vegetative samples within gametophyte developmental stages against genes showing differential upregulation between at least two stages of gametophyte development specifically in the notch (Ntc only) and genes showing co-upregulation between the same stages of notch and thallus development (Ntc + Thl). Venn diagrams show the comparison of all DE candidates (**a**) and the notch-enriched (**b**), notch-specific (**c**) and notch-FPKM<1 subsets (**d**) identified in **Fig. 4s**. Numbers in brackets represent the total number of genes within each dataset. Source data are provided as a **Source Data** file.

a

Protein	Species	E-value	% identity	Align len	Strands	Query ID	Query from	Query to	Target from	Target to	Bitscore	# identical	Positives	Gaps	Query len	Target len
Ceric.31G036600.1.p	C.richardii v2.1	0	99	840	+/+	CrANT_Bui_et_al_2017	1	838	113	951	1671.75	829	829	3	838	952
Ceric.31G036600.4.p	C.richardii v2.1	0	99	811	+/+	CrANT_Bui_et_al_2017	30	838	7	816	1612.43	799	800	3	838	817
Ceric.31G036600.3.p	C.richardii v2.1	0	99	797	+/+	CrANT_Bui_et_al_2017	44	838	1	796	1580.46	786	786	3	838	797
Ceric.31G036600.2.p	C.richardii v2.1	0	94	840	+/+	CrANT_Bui_et_al_2017	1	838	113	912	1568.13	790	790	42	838	913
Ceric.12G002600.1.p	C.richardii v2.1	1.07E-124	49	550	+/+	CrANT_Bui_et_al_2017	355	837	516	1026	401.364	268	315	106	838	1028
Ceric.10G056200.1.p	C.richardii v2.1	2.07E-105	81	218	+/+	CrANT_Bui_et_al_2017	357	571	444	660	347.051	176	189	4	838	915
Ceric.10G056200.2.p	C.richardii v2.1	2.68E-105	81	218	+/+	CrANT_Bui_et_al_2017	357	571	422	638	346.28	176	189	4	838	893
Ceric.26G003300.1.p	C.richardii v2.1	2.97E-102	80	215	+/+	CrANT_Bui_et_al_2017	357	568	303	513	332.798	172	185	7	838	683
Ceric.32G008600.3.p	C.richardii v2.1	1.54E-101	58	320	+/+	CrANT_Bui_et_al_2017	257	568	67	373	330.102	187	225	21	838	665
Ceric.32G008600.2.p	C.richardii v2.1	2.05E-99	58	320	+/+	CrANT_Bui_et_al_2017	257	568	339	645	331.643	187	225	21	838	937
Ceric.32G008600.1.p	C.richardii v2.1	2.71E-99	58	320	+/+	CrANT_Bui_et_al_2017	257	568	345	651	331.257	187	225	21	838	943

b

Protein	Species	E-value	% identity	Align len	Strands	Query ID	Query from	Query to	Target from	Target to	Bitscore	# identical	Positives	Gaps	Query len	Target len
Ceric.33G031700.1.p	C.richardii v2.1	0	100	370	+/+	CrLFY1_Plackett_et_al_2018	1	370	112	481	747.658	370	370	0	370	482
Ceric.33G031700.2.p	C.richardii v2.1	0	100	370	+/+	CrLFY1_Plackett_et_al_2018	1	370	100	469	747.273	370	370	0	370	470
Ceric.33G031700.3.p	C.richardii v2.1	0	100	370	+/+	CrLFY1_Plackett_et_al_2018	1	370	1	370	745.732	370	370	0	370	371
Ceric.18G076300.3.p	C.richardii v2.1	0	85	379	+/+	CrLFY1_Plackett_et_al_2018	1	370	17	394	650.588	323	342	10	370	395
Ceric.18G076300.1.p	C.richardii v2.1	0	85	379	+/+	CrLFY1_Plackett_et_al_2018	1	370	17	394	650.588	323	342	10	370	395
Ceric.33G031700.4.p	C.richardii v2.1	3.22E-179	98	251	+/+	CrLFY1_Plackett_et_al_2018	120	370	11	261	498.819	246	249	0	370	262
Ceric.18G076300.2.p	C.richardii v2.1	8.84E-164	90	247	+/+	CrLFY1_Plackett_et_al_2018	124	370	5	251	459.144	222	229	0	370	252
Ceric.18G076300.4.p	C.richardii v2.1	3.48E-157	91	235	+/+	CrLFY1_Plackett_et_al_2018	136	370	1	235	441.81	213	220	0	370	236

Supplementary Fig. 15. Validation of notch reproductive candidate genes as previously-published embryo-related genes.

a,**b**, BLASTp search results of *C. richardii* genome v2.1 against the amino acid sequences of CrANT (**a**) and CrLFY1 (**b**), showing the genomic loci with the closest identity. Closest matches were identified as Ceric.31G036600 and Ceric.33G031700, respectively.

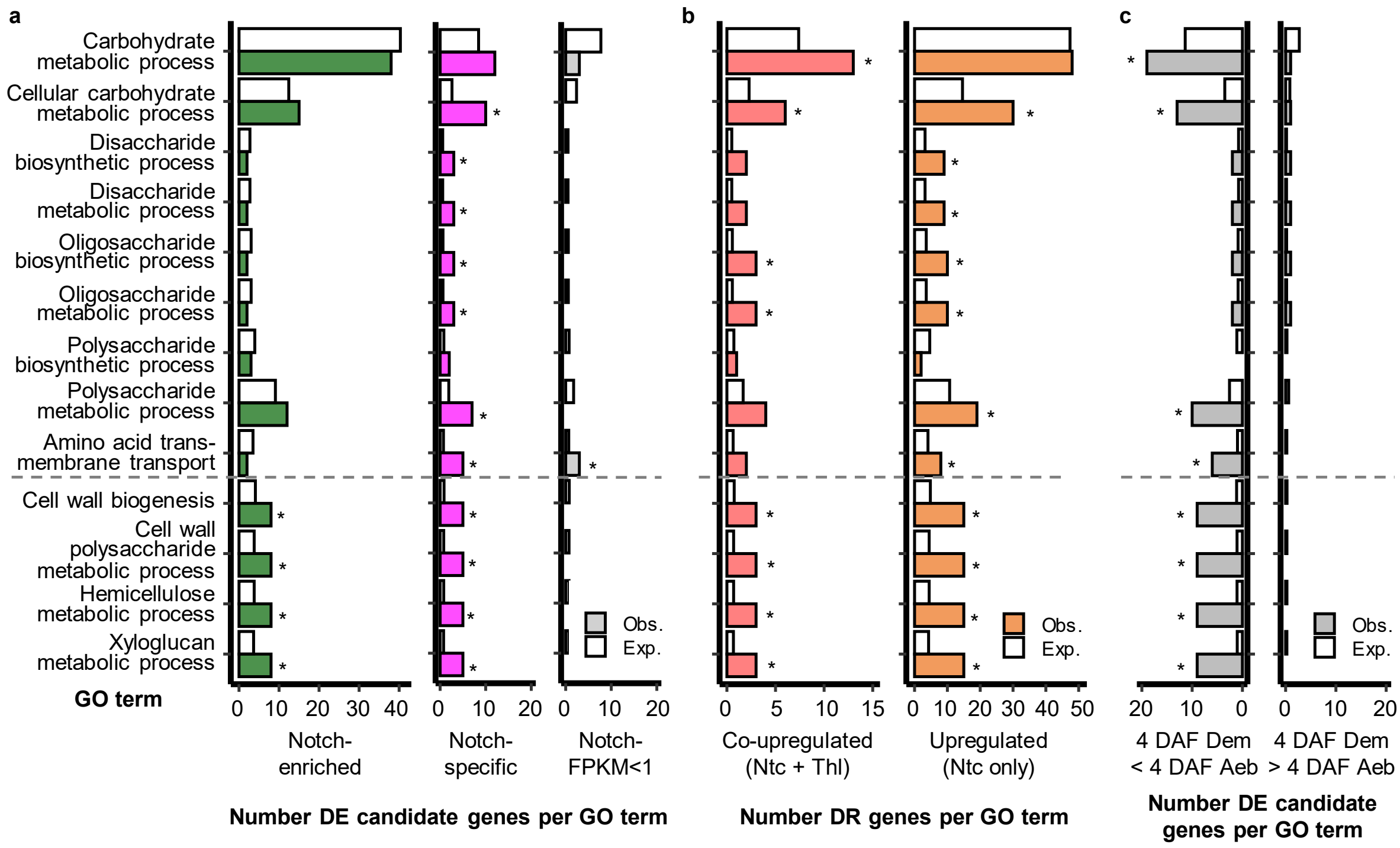

Supplementary Fig. 16. Additional enrichment testing of nutrient-related GO terms within notch reproductive candidates.

Enrichment of GO terms relating to nutrients (carbohydrate metabolism and amino acid transport) vs cell wall biosynthesis, comparing within each DE notch expression category (**a**), genes showing differential regulation across gametophyte development (**b**) and within genes showing significantly increased or decreased abundance in 4 DAF embryo-bearing archegonia vs 4 DAF dissected embryos (**c**). Barplots show the observed ('Obs.') and expected ('Exp.') gene frequencies per GO term (n = 1). Enrichment was tested using Fisher's Exact test (one-sided, greater than). Asterisks denote significant enrichment ($p < 0.05$) in genes with that GO term compared to that expected based on the whole genome. Exact P-values are given in **Supplementary Data 5**. Source data are provided as a **Source Data** file.

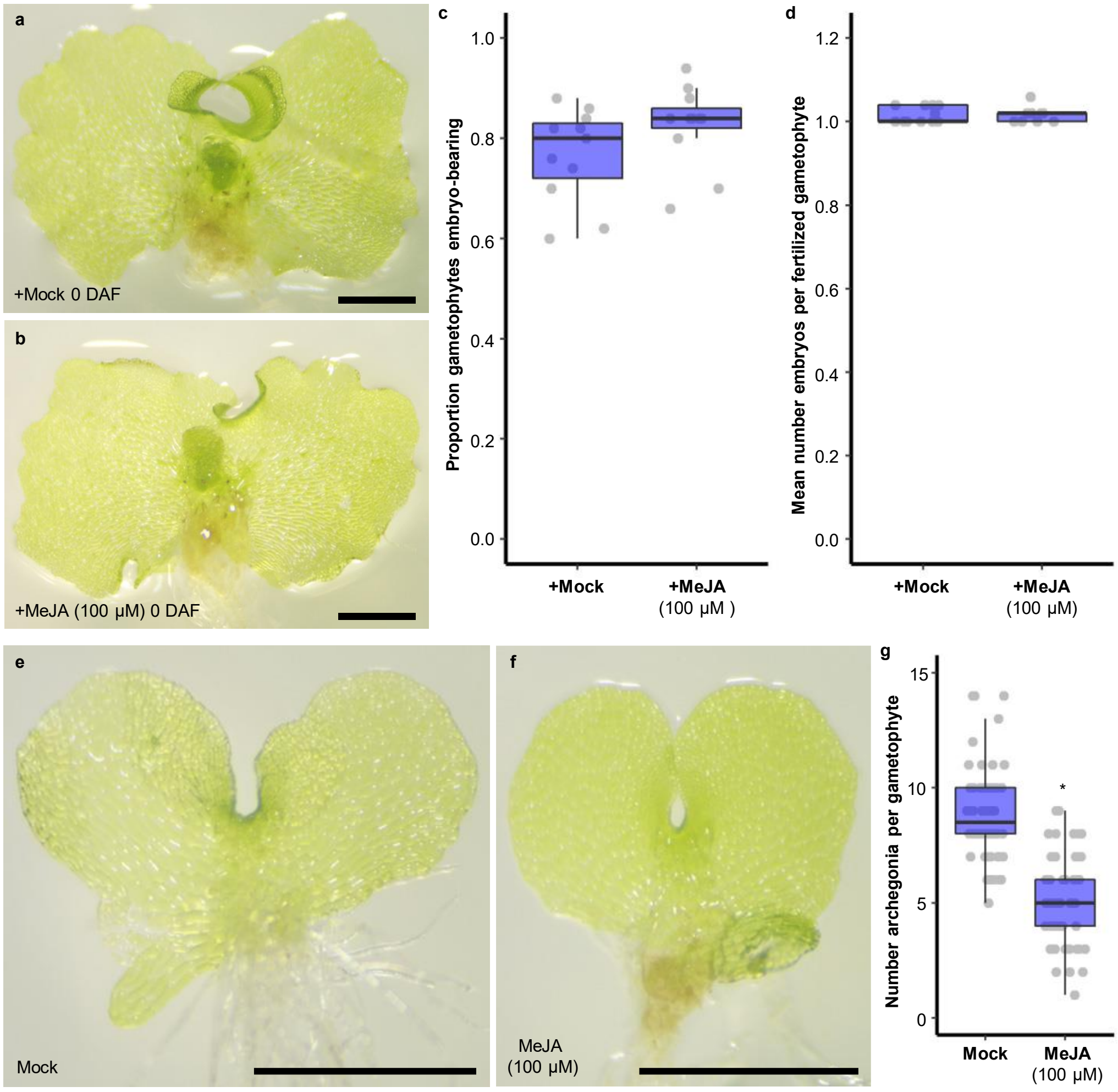

Supplementary Fig. 17. JA negatively regulates notch meristem development.

a-d, Effect of JA on post-fertilization development. 14 day-old gametophytes were flooded with mock solution (**a**) or 100 µM methyl jasmonate (MeJA) (**b**) and fertilization success scored at 6-7 DAF via proportion of gametophytes bearing at least one embryo per experimental population (50 gametophytes) (**c**) and mean embryo number per embryo-bearing population (50 gametophytes) (**d**). Boxplots denote the data median and interquartile range (box) ±1.5x the interquartile range (whiskers). Pairwise comparisons were performed using two-tailed Pairwise T tests (**c**,**d**). P = 0.11 (c), P = 0.9419 (d). *n* = 11 (**c**,**d**). Experiment repeated 4 times. **e-g**, Effect of JA on pre-fertilization gametophyte development. 7 day-old gametophytes were moved to media containing mock treatment (**e**) or 100 µM MeJA (f), and the number of visible archegonia counted at 14 days old (**g**). *n* = 60 (20 per experimental replicate, 3 replicates). Scale bars = 1mm. Boxplots (**c**, **d**, **g**) denote the data median and interquartile range (box) ±1.5x the interquartile range (whiskers). Pairwise comparisons were performed using two-tailed Pairwise T tests (**c**,**d**) and two-tailed Mann-Whitney tests (**g**). Asterisks denote a significant difference (P = 0.0000000000000015) between treatments. Experiment repeated 4 times. Statistical analyses are given in **Supplementary Data 6**. Source data are provided as a **Source Data** file.

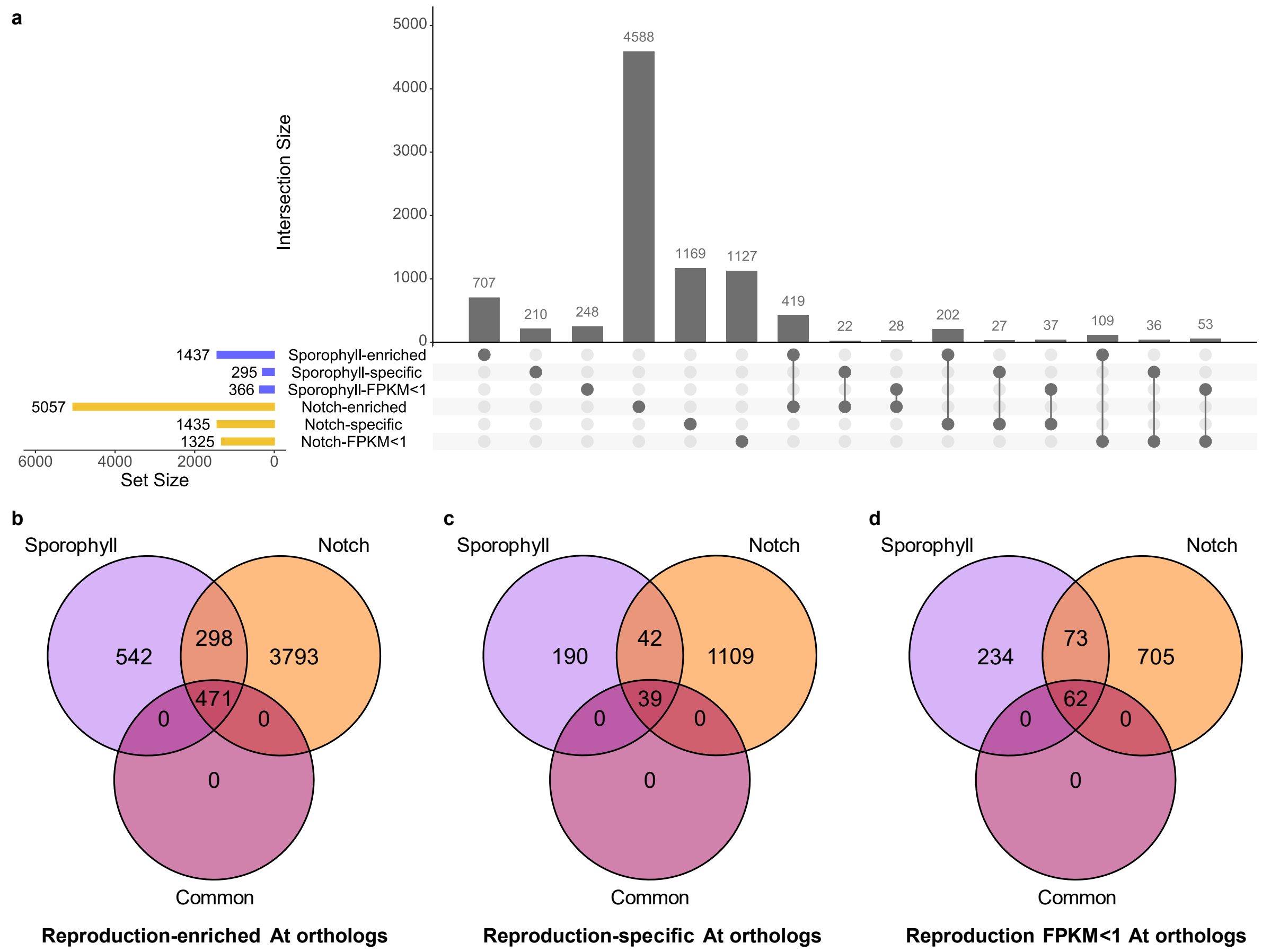

Supplementary Fig. 18. Gene orthology of *Ceratopteris* reproductive candidates separated by expression category.

a, Upset plot showing overlap between reproductive candidate genes in the *Ceratopteris* sporophyll (blue) and notch (yellow) by expression category.

e-g, Frequency and overlap of nonredundant *Arabidopsis* orthologs of sporophyll, notch and common candidates within reproduction-enriched (**e**), reproduction-specific (**f**) and FPKM<1 expression categories (**g**). Source data are provided as a **Source Data** file.

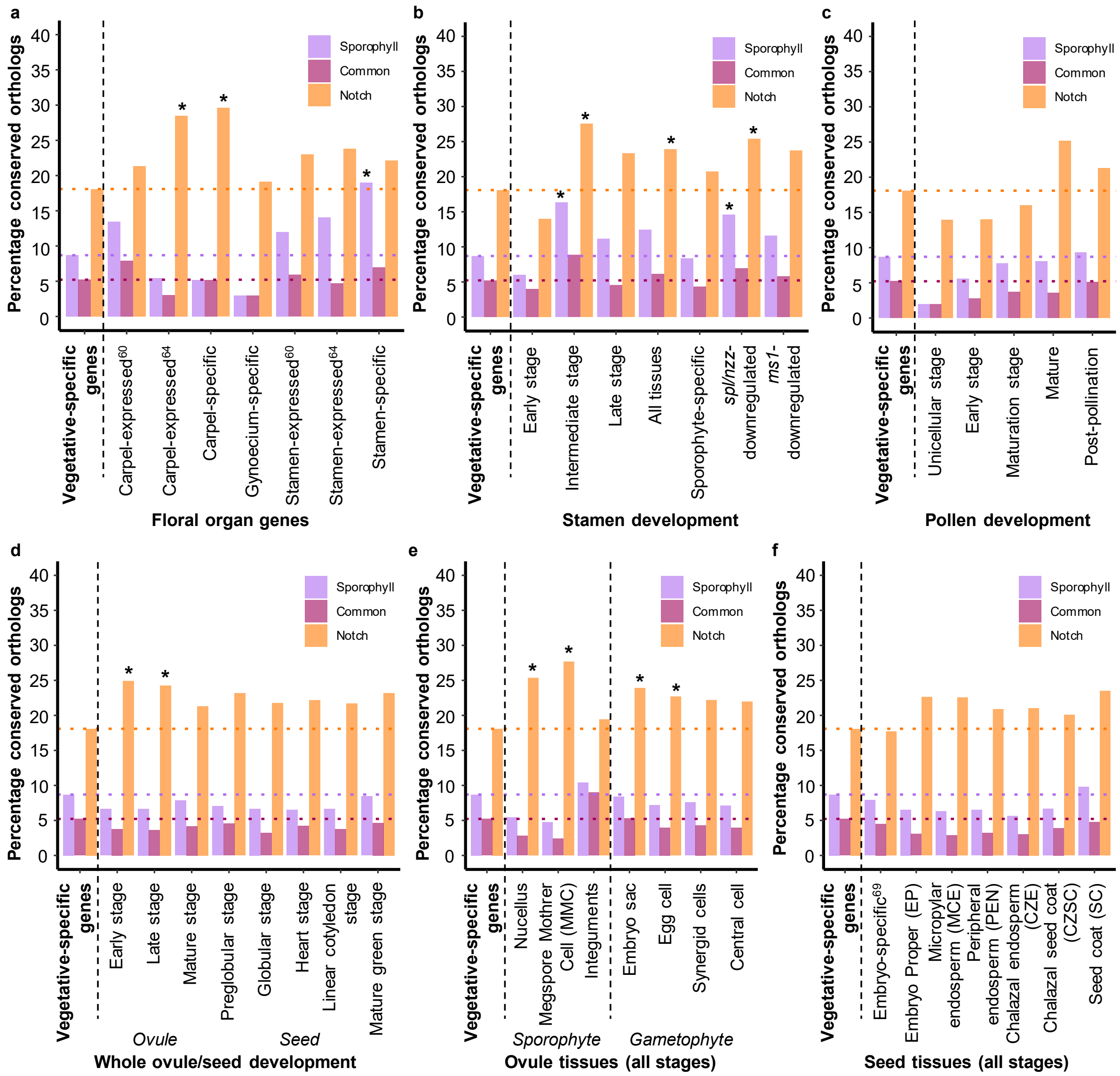

Supplementary Fig. 19. Ortholog enrichment testing of *Arabidopsis* reproductive gene networks using all notch and all sporophyll DE reproductive candidates.

Enrichment testing of *Arabidopsis* gene orthologs for all sporophyll, notch and common reproductive candidates in *Arabidopsis* floral organs (**a**), stamen tissues (**b**), pollen development (**c**), whole ovule/seed development (**d**), ovule tissues (**e**) and seed tissues (**f**) against published vegetative-specific genes (see **Fig. 7** for dataset sources). Barplots show the overlap of candidate orthologs against each target as a proportion of that dataset (n = 1). Dotted horizontal lines track the overlap of each candidate ortholog dataset with the vegetative control. Enrichment was tested using Fisher's Exact test (one-sided, greater than), adjusted for multiple comparisons (Benjamini-Hochberg method). Asterisks denote a significant increase ($P_{adj} < 0.05$) in the proportion of overlapping genes between candidate orthologs and the target dataset compared to the overlap with vegetative-specific genes. Adjusted P-values are given in **Supplementary Data 9**. Source data are provided as a **Source Data** file.

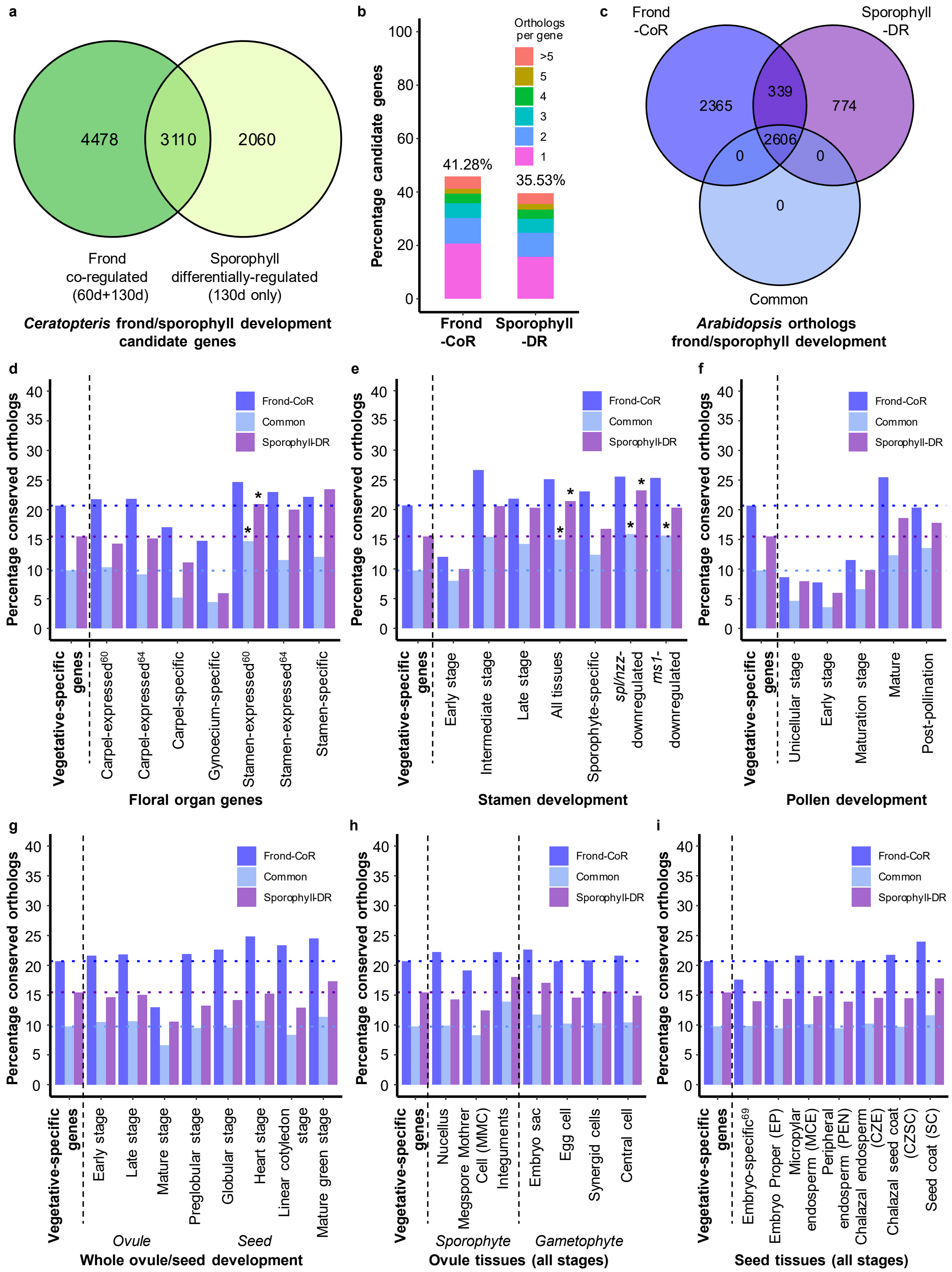

Supplementary Fig 20. Frond/sporophyll differentially-regulated candidate ortholog enrichment in *Arabidopsis* reproductive gene networks.

a, Venn comparison of *Ceratoperis* genes showing differential upregulation during sporophyll development vs genes co-upregulation during frond development (60d + 130d).

b, Barplot showing the proportion of candidate genes with associated *Arabidopsis* orthologs, with bars broken into classes denoting the number of orthologs per *Ceratopteris* gene (*n* = 1).
c, Venn comparison of the *Arabidopsis* orthologs of *Ceratopteris* genes in (**a**), including the separately-identified orthologs of *Ceratopteris* genes common to both candidate groups.
d-i, Enrichment testing of *Arabidopsis* gene orthologs for the frond co-upregulated, sporophyll differentially-upregulated and common candidates within published *Arabidopsis* reproductive gene datasets (see **Fig. 7** for dataset sources). Barplots show the overlap of candidate orthologs against each target as a proportion of that dataset (*n* = 1). Dotted horizontal lines track the overlap of each candidate ortholog dataset with the vegetative control. Enrichment was tested using Fisher's Exact test (one-sided, greater than), adjusted for multiple comparisons (Benjamini-Hochberg method). Asterisks denote a significant increase (P_{adj} < 0.05) in the proportion of overlapping genes between candidate orthologs and the target reproductive organ compared to the overlap with vegetative-specific genes. Adjusted P-values are given in **Supplementary Data 9**. Source data are provided as a **Source Data** file.

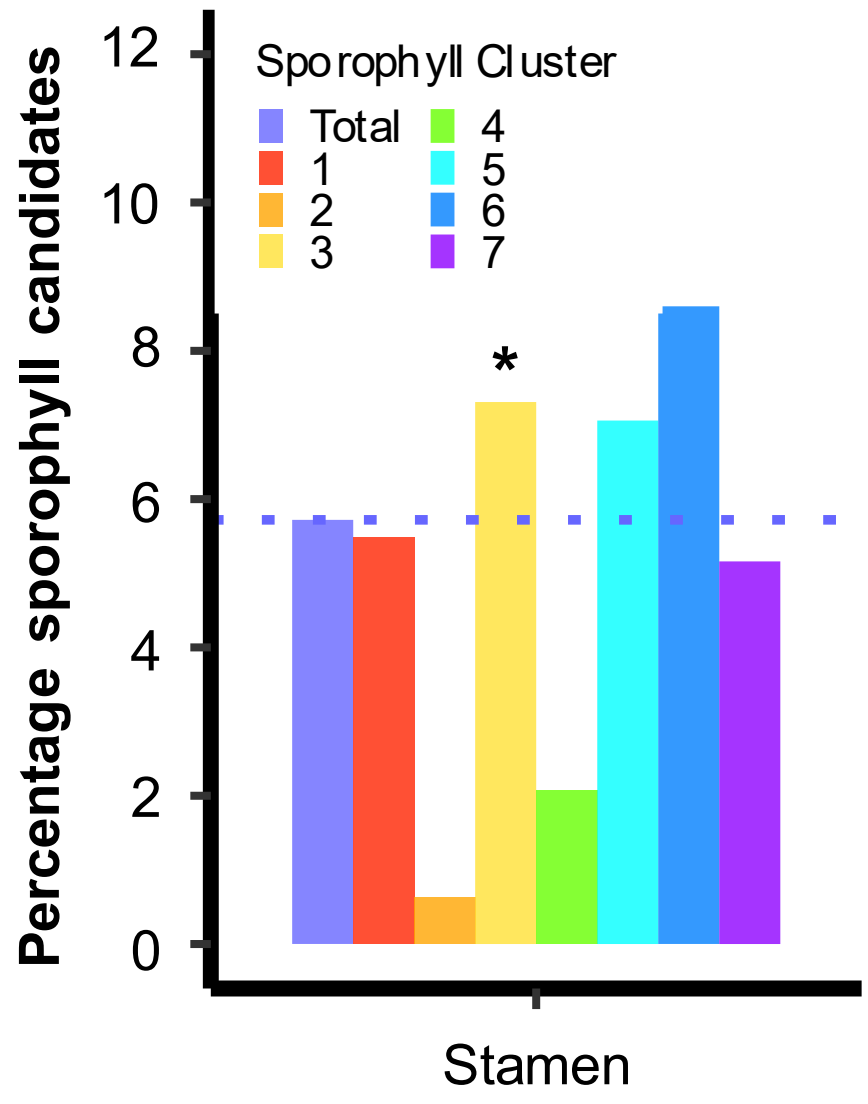

Supplementary Fig 21. Reciprocal enrichment testing of sporophyll-conserved *Arabidopsis* stamen genes against sporophyll pHeatmap clusters.

Enrichment testing of *Ceratopteris* gene orthologs of Arabidopsis stamen genes enriched in sporophyll DE reproductive candidates against sporophyll DE reproductive candidates.

Barplot shows the overlap of candidate orthologs against each sporophyll pHeatmap cluster as a proportion of that dataset (n = 1). Dotted horizontal line tracks the baseline overlap with all sporophyll reproductive candidates. Enrichment was tested using Fisher's Exact test (one-sided, greater than), adjusted for multiple comparisons (Benjamini-Hochberg method). Asterisks denote a significant increase (P_{adj} = 0.04305) in the proportion of overlapping genes between candidate orthologs and the target cluster compared to the overlap with all sporophyll candidates. Adjusted P-values are given in **Supplementary Data 10.** Source data are provided as a **Source Data** file.

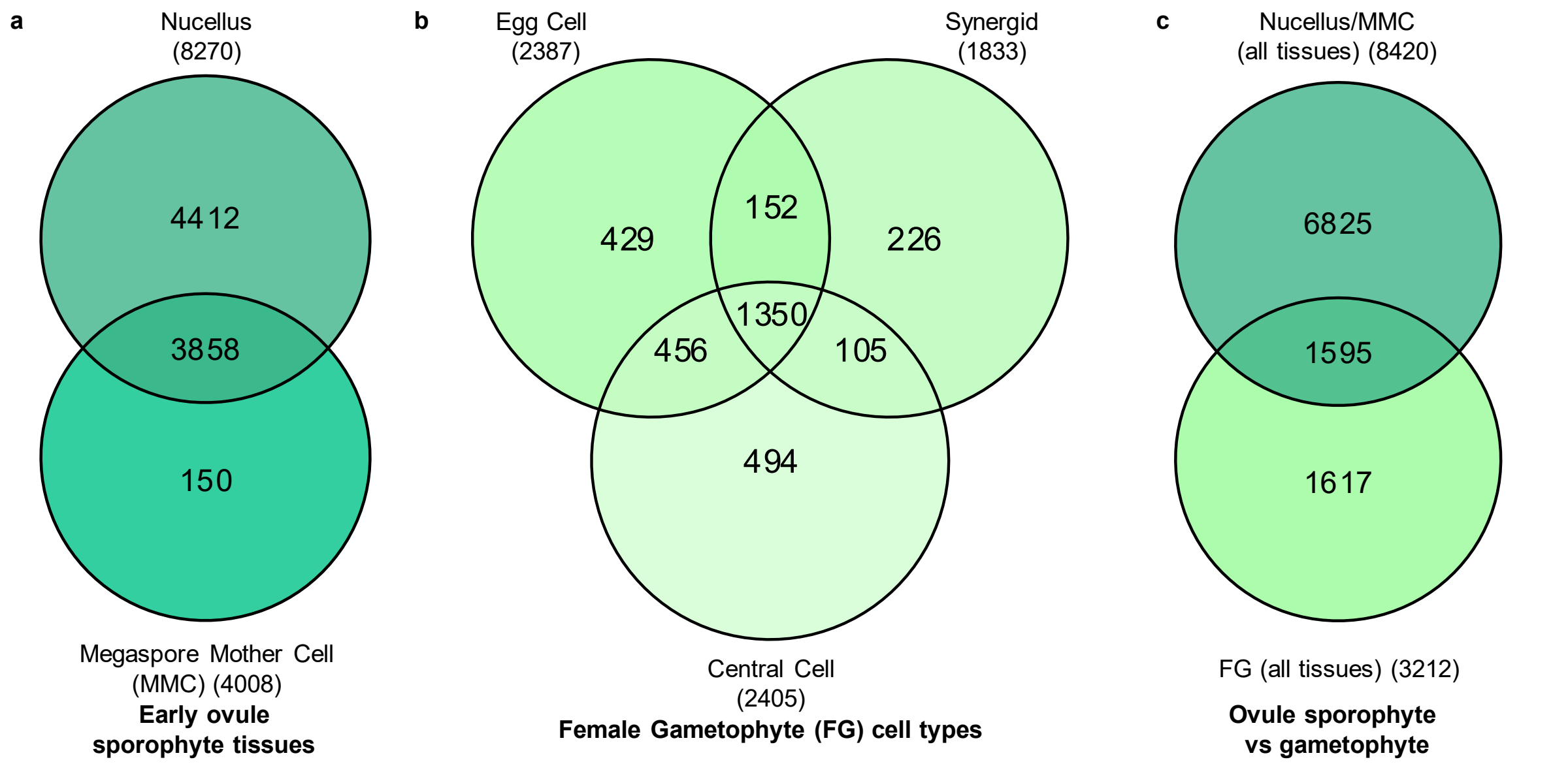

Supplementary Fig. 22. Specificity of *Arabidopsis* genes between ovule tissues.
a-c, Venn comparisons of published *Arabidopsis* reproductive genes (see **Supplementary Dataset 9**) between tissues of the premeiotic ovule (**a**), the mature female gametophyte (**b**), and between the totals of these two groups (**c**). Source data are provided as a **Source Data** file.

Table 1 *Ceratopteris* notch reproductive candidate genes expressed in the embryo-bearing archegonium conserved with *Arabidopsis* ovule/seed genes.

***Ceratopteris* transcript**	**Notch Cluster**	**Enriched GO term (process-specific)**	***Arabidopsis* ortholog**	**Ortholog name(s)**	***Arabidopsis* ovule function**	***Arabidopsis* molecular function**
Ceric.1Z277300	3	RNA processing	AT1G01040	ASU1, ATDCL1, CAF, DCL1, EMB60, EMB76, SIN1, SUS1	Integument development[143]	MicroRNA regulation
Ceric.02G091600; Ceric.36G057000	2	DNA methylation	AT5G14620; AT5G15380	DMT7, DRM2; DRM1	Megaspore Mother Cell specification[94]	RNA-dependent DNA Methylation (RdDM)
Ceric.01G010000	2	DNA repair	AT3G18524	MSH2	Meiotic recombination[53]	Chromosome mis-pairing repression
Ceric.32G069600	2	DNA repair	AT3G24495	MSH7	Meiotic recombination[54]	Crossover repression
Ceric.03G086200	3	Protein modification process	AT2G21470	SAE2	Meiotic recombination[55]	SUMO E1 ligase
Ceric.13G086400	2	DNA repair	AT2G45280	RAD51C	Meiosis[56]	Homologous chromosome pairing and recombination
Ceric.15G013100	2	Chromosome organization; ncRNA processing	AT5G63920	TOP3A	Meiotic recombination[57]	Crossover resolution
Ceric.22G002100	3	DNA replication	AT5G22010	RFC1	Meiosis[58]	Double-strand break repair
Ceric.04G002100	2	DNA replication	AT4G02060	MCM7/ PRL	FG development (cell division plane)[144]	DNA helicase
Ceric.14G005300	2	RNA processing	AT3G43920	DCL3	FG development[94]	RNA-dependent DNA Methylation (RdDM)

Ceric.22G046500	3	RNA processing	AT3G43920	DCL3	FG development[94]	RNA-dependent DNA Methylation (RdDM)
Ceric.15G036100	2	ncRNA processing	AT5G16750	TOZ	Embryogenesis (cell division plane)[145]	rRNA processing
Ceric.20G088700; Ceric.33G055700	3	Protein phosphory-lation	AT4G32830; AT2G25880	AUR1; AUR2	Embryogenesis (cell division plane)[146]	Protein kinase
Ceric.10G003400	3	DNA replication	AT1G08260; AT2G27120	ABO4, EMB142, EMB2284, EMB529, ESD7, POL2A, TIL1; POL2B, TIL2	Embryogenesis (cell division, patterning)[147]	DNA polymerase
Ceric.36G039200; Ceric.36G048900	2	Histone lysine methylation	AT5G13960	KYP, SDG33, SUVH4	Embryogenesis (imprinting)[148]	DNA methylation
Ceric.10G056200; Ceric.26G003300	3	Regulation of DNA-templated transcription	AT1G51190; AT3G20840; AT5G10510; AT5G17430; AT5G57390; AT5G65510	PLT2; PLT1; PLT3; BBM; EMK; PLT7	Embryogenesis; endosperm (proliferation)[149]	Transcription factor
Ceric.12G044400; Ceric.15G020500	3	Cell cycle; ncRNA processing	AT3G47460; AT5G62410	ATSMC2B; ATSMC4, SMC2, TTN3	Endosperm (proliferation/ cellularisation)[150]	DNA Condensin
Ceric.05G021600	2	Cell cycle; ncRNA processing	AT2G27170	SMC3, TTN7	Endosperm (proliferation/ cellularisation)[150]	DNA Cohesin
Ceric.28G053000	2	DNA repair; DNA recombination	AT1G08130; AT1G49250	LIG1	Endosperm (proliferation/ cellularisation)[151]	DNA ligase
Ceric.07G032800; Ceric.28G025600; Ceric.30G070100	2	Regulation of gene expression	AT5G05610; AT3G11200	AL1; AL2	Seed germination[152]	Histone methylation

143. Golden, T.A. *et al. Short Integuments1/suspensor1/carpel Factory*, a dicer homolog, is a maternal effect gene required for embryo development in Arabidopsis. *Plant Physiol.* **130**, 808-822 (2002).

144. Holding, D.R., Springer, P.S. The *Arabidopsis* gene *PROLIFERA* is required for proper cytokinesis during seed development. *Planta* **214**, 373-382 (2002).

145. Griffith, M.E. *et al.* The *TORMOZ* gene encodes a nuclear protein required for regulated division planes and embryo development in *Arabidopsis*. *The Plant Cell* **19**, 2246-2263 (2007).

146. Van Damme, D. *et al. Arabidopsis* α Aurora kinases function in formative cell division plane orientation. *Plant Cell* **23**, 4013-4024 (2011).

147. Jenik, P.D, Jurkata R.E.J. & Barton M.K. Interactions between the cell cycle and embryonic patterning in *Arabidopsis* uncovered by a mutation in DNA Polymerase ε. *Plant Cell* **17**, 3362-3377 (2005).

148. Autran, D. *et al.* Maternal epigenetic pathways control parental contributions to Arabidopsis early embryogenesis. *Cell* **145**, 707-719 (2011).

149. Chen, B. *et al.* BABY BOOM regulates early embryo and endosperm development. *PNAS* **119**, e2201761119 (2022).

150. Tzafrir, I. *et al.* Diversity of TITAN functions in Arabidopsis seed development. *Plant Physiol.* **128**, 38-51 (2002).

151. Andreuzza, S. *et al.* DNA LIGASE I exerts a maternal effect on seed development in *Arabidopsis thaliana*. Development 137, 73-81 (2010).

152. Molitor, A.M., Bu, Z., Yu, Y., Sen, W.H. *Arabidopsis* AL PHD-PRC1 complexes promote seed germination through H3K4me3-to-H3K27me3 chromatin state switch in repression of seed developmental genes. *PLoS Genet.* **10**, e1004091 (2014).
